# Publisher Correction: Conservative route to genome compaction in a miniature annelid

**DOI:** 10.1038/s41559-020-01366-z

**Published:** 2020-11-25

**Authors:** José M. Martín-Durán, Bruno C. Vellutini, Ferdinand Marlétaz, Viviana Cetrangolo, Nevena Cvetesic, Daniel Thiel, Simon Henriet, Xavier Grau-Bové, Allan M. Carrillo-Baltodano, Wenjia Gu, Alexandra Kerbl, Yamile Marquez, Nicolas Bekkouche, Daniel Chourrout, Jose Luis Gómez-Skarmeta, Manuel Irimia, Boris Lenhard, Katrine Worsaae, Andreas Hejnol

**Affiliations:** 1 Sars International Centre for Marine Molecular Biology, University of Bergen, Bergen, Norway; 2 School of Biological and Chemical Sciences, Queen Mary University of London, London, UK; 3 Max Planck Institute of Molecular Cell Biology and Genetics, Dresden, Germany; 4 Molecular Genetics Unit, Okinawa Institute of Science and Technology, Graduate University, Onna, Japan; 5 Department of Biological Sciences, University of Bergen, Bergen, Norway; 6 Institute for Clinical Sciences and MRC London Institute of Medical Sciences, Faculty of Medicine, Imperial College London, London, UK; 7 Department of Vector Biology, Liverpool School of Tropical Medicine, Liverpool, UK; 8 Department of Biology, University of Copenhagen, Copenhagen, Denmark; 9 Centre for Genomic Regulation, Barcelona Institute of Science and Technology, Barcelona, Spain; 10 Centro Andaluz de Biología del Desarrollo, Consejo Superior de Investigaciones Cientificas-Universidad Pablo de Olavide-Junta de Andalucía, Seville, Spain; 11 Universitat Pompeu Fabra, Barcelona, Spain; 12 ICREA, Barcelona, Spain; 13 Present Address: Centre for Life’s Origins and Evolution, Department of Genetics, Evolution and Environment, University College London, London, UK; 14 Present Address: Living Systems Institute, University of Exeter, Exeter, UK; 15 Present Address: Centrum für Naturkunde, Universität Hamburg, Hamburg, Germany

**Keywords:** Molecular evolution, Comparative genomics

Correction to: *Nature Ecology & Evolution* 10.1038/s41559-020-01327-6, published online 16 November 2020.

This Article was originally mistakenly published behind the paywall, but should have been open access; this has been amended and the Article is now freely available under a Creative Commons licence CC BY 4.0.

