# Supplementary material for: Conservative route to genome compaction in a miniature annelid
Source: Nat Ecol Evol. 2020 Nov 16;5(2):231–42. doi: 10.1038/s41559-020-01327-6 (PMC7854359; doi:10.1038/s41559-020-01327-6)
Supplement: Supplementary file 1 — Supplementary Figs. 1–8, Tables 1–11, Notes 1 and 2 and references. [file 41559_2020_1327_MOESM1_ESM.pdf]

nature research
https://doi.org/10.1038/s41559-020-01327-6

Supplementary information

Conservative route to genome compaction in a miniature annelid

In the format provided by the authors and unedited

Supplementary Information

Conservative route to extreme genome compaction in a miniature annelid

José M. Martín-Durán, Bruno C. Vellutini, Ferdinand Marlétaz, Viviana Cetrangolo, Nevena Cvetesic, Daniel Thiel, Simon Henriet, Xavier Grau-Bové, Allan Carrillo-Baltodano, Wenjia Gu, Alexandra Kerbl, Yamile Marquez, Nicolas Bekkouche, Daniel Chourrout, Jose Luis Gómez-Skarmeta, Manuel Irimia, Boris Lenhard, Katrine Worsaae, Andreas Hejnol

Index:

- **Supplementary Figure 1.** Spectra-cn plots for *D. gyrociliatus* and *T. axi*
- **Supplementary Figure 2.** GenomeScope2.0 genome profiling of *D. gyrociliatus* and *T. axi*.
- **Supplementary Figure 3.** Smudgeplot ploidy estimation for *D. gyrociliatus* and *T. axi*.
- **Supplementary Figure 4.** Wnt ligand orthology assignment
- **Supplementary Figure 5.** Frizzled receptor orthology assignment
- **Supplementary Figure 6.** TGF-β ligand orthology assignment
- **Supplementary Figure 7.** TGF-β receptor orthology assignment
- **Supplementary Figure 8.** CTCF orthology assignment
- **Supplementary Table 1.** Genome assembly statistics
- **Supplementary Table 2.** Transcriptome assembly statistics
- **Supplementary Table 3.** Automated annotation of repeats and transposable elements in annelid genomes

- **Supplementary Table 4.** Manually identified repeats and TEs in *D. gyrociliatus* genome
- **Supplementary Table 5.** Comparison of TE abundance and diversity in small animal genomes
- **Supplementary Table 6.** List of species and gene annotation files used in this study
- **Supplementary Table 7.** Gene family statistics in annelid proteomes
- **Supplementary Table 8.** Estimation of GPCRs in *D. gyrociliatus* and other annelid and bilaterian genomes
- **Supplementary Table 9.** DNA double-strand break machinery in *D. gyrociliatus*
- **Supplementary Table 10.** Hippo and PI3K/AKT/mTOR pathways in *D. gyrociliatus*
- **Supplementary Table 11.** Presence/absence of MNT/MAD in Metazoa
- **Supplementary Note 1.** Background information on *D. gyrociliatus*
- **Supplementary Note 2.** Phylogenetic position of *D. gyrociliatus*: background
- **Supplementary References**

Supplementary Figures

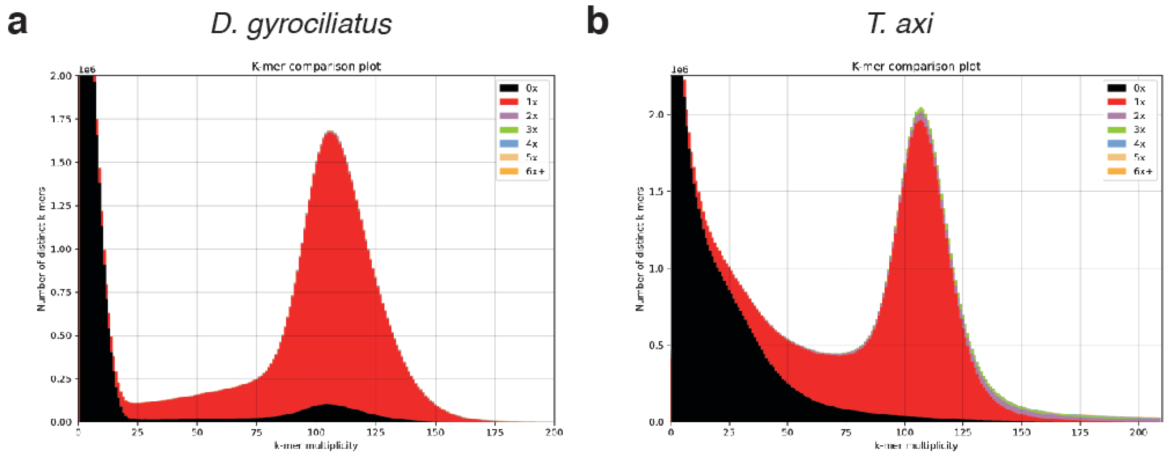

Supplementary Figure 1 | Spectra-cn plots for *D. gyrociliatus* and *T. axi*. (**a**) Spectra copy number for *D. gyrociliatus* based on the comparison between Illumina reads and the final PacBio assembly. The assembly contains 93.60% of the k-mers present in the reads and has a low level of k-mer duplications. (**b**) Spectra copy number for *T. axi* based on the comparison between Illumina reads and the final Illumina assembly. The assembly contains 98.23% of the k-mers present in the reads and also has low levels of duplicated k-mers.

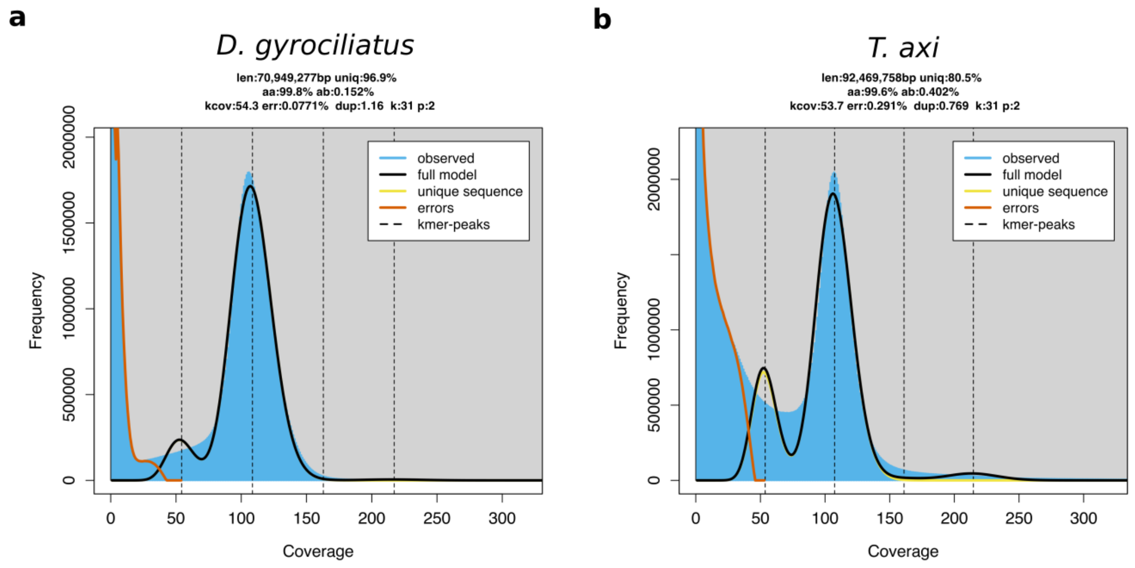

Supplementary Figure 2 | GenomeScope2.0 genome profiling of *D. gyrociliatus* and *T. axi.* (**a**) 31-mer spectrum and fitted model for the normalised Illumina reads of *D. gyrociliatus*. The estimated haploid length is 70,949,277 bp (68,718,074 bp unique and 2,231,203 bp of repeats) with 0.16% estimated heterozygosity and a model fit of 92%. (**b**) 31-mer spectrum and fitted model for the normalised Illumina reads of *T. axi*. The estimated haploid length is 92,469,758 bp (18,066,808 bp unique and 74,402,950 bp of repeats) with 0.42% estimated heterozygosity and a model fit of 84%.

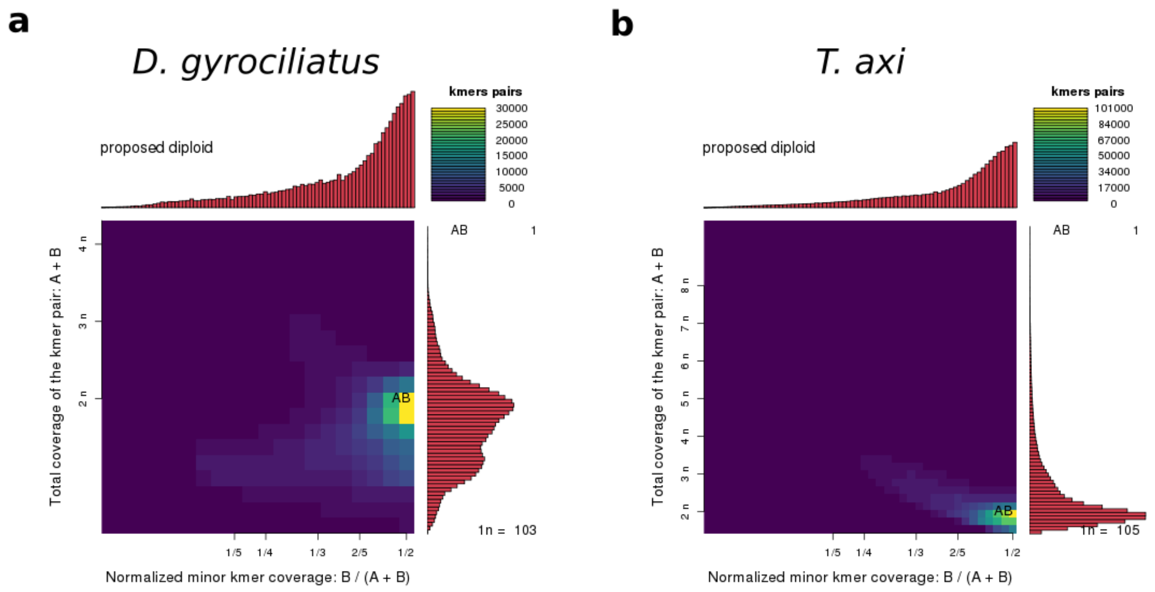

Supplementary Figure 3 | Smudgeplot ploidy estimation for *D. gyrociliatus* and *T. axi*. (**a**) Genome structure of *D. gyrociliatus* showing a predominance of "AB" (diploid) k-mer pairs. (**b**) Genome structure of *T. axi* also shows a predominance of "AB" (diploid) k-mer pairs and the absence of higher ploidy levels.

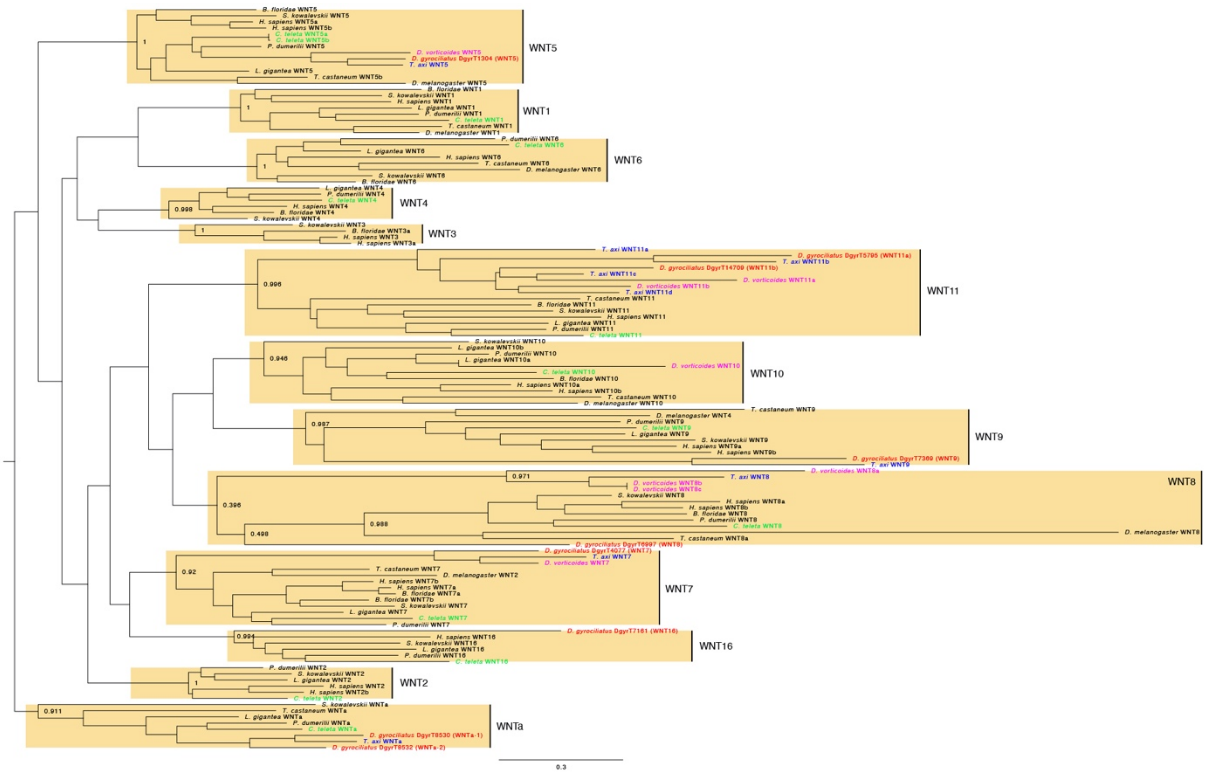

Supplementary Figure 4 | Wnt ligand orthology assignment. Maximum likelihood tree of Wnt ligands, including those putative sequences identified in *D. gyrociliatus* (in red), *T. axi* (in blue), *D. vorticoides* (in pink) and *C. teleta* (in green). Each major orthology group is highlighted with a light-yellow box, and only bootstrap values supporting these clades are depicted.

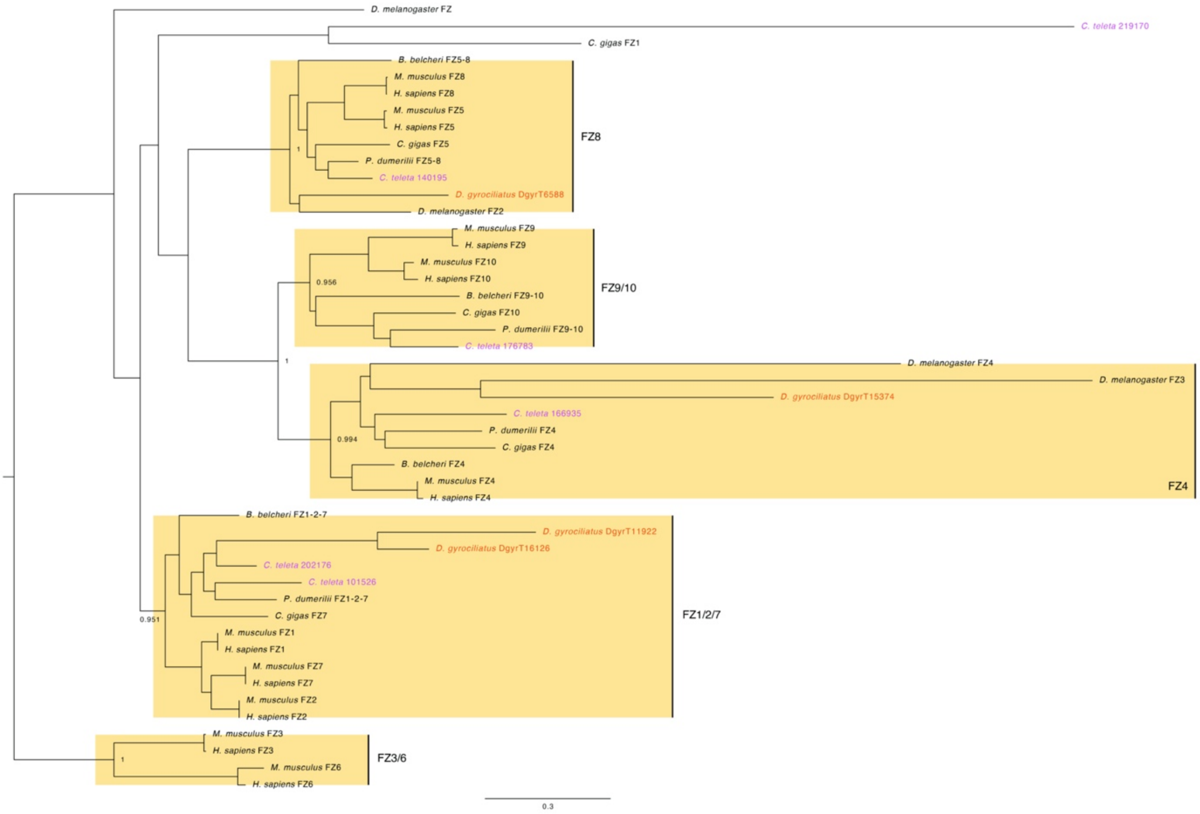

Supplementary Figure 5 | Frizzled receptor orthology assignment. Maximum likelihood tree of Frizzled receptors, including those putative sequences identified in *D. gyrociliatus* (in red) and *C. teleta* (in violet). Each major orthology group is highlighted with a light-yellow box, and only bootstrap values supporting these clades are depicted.

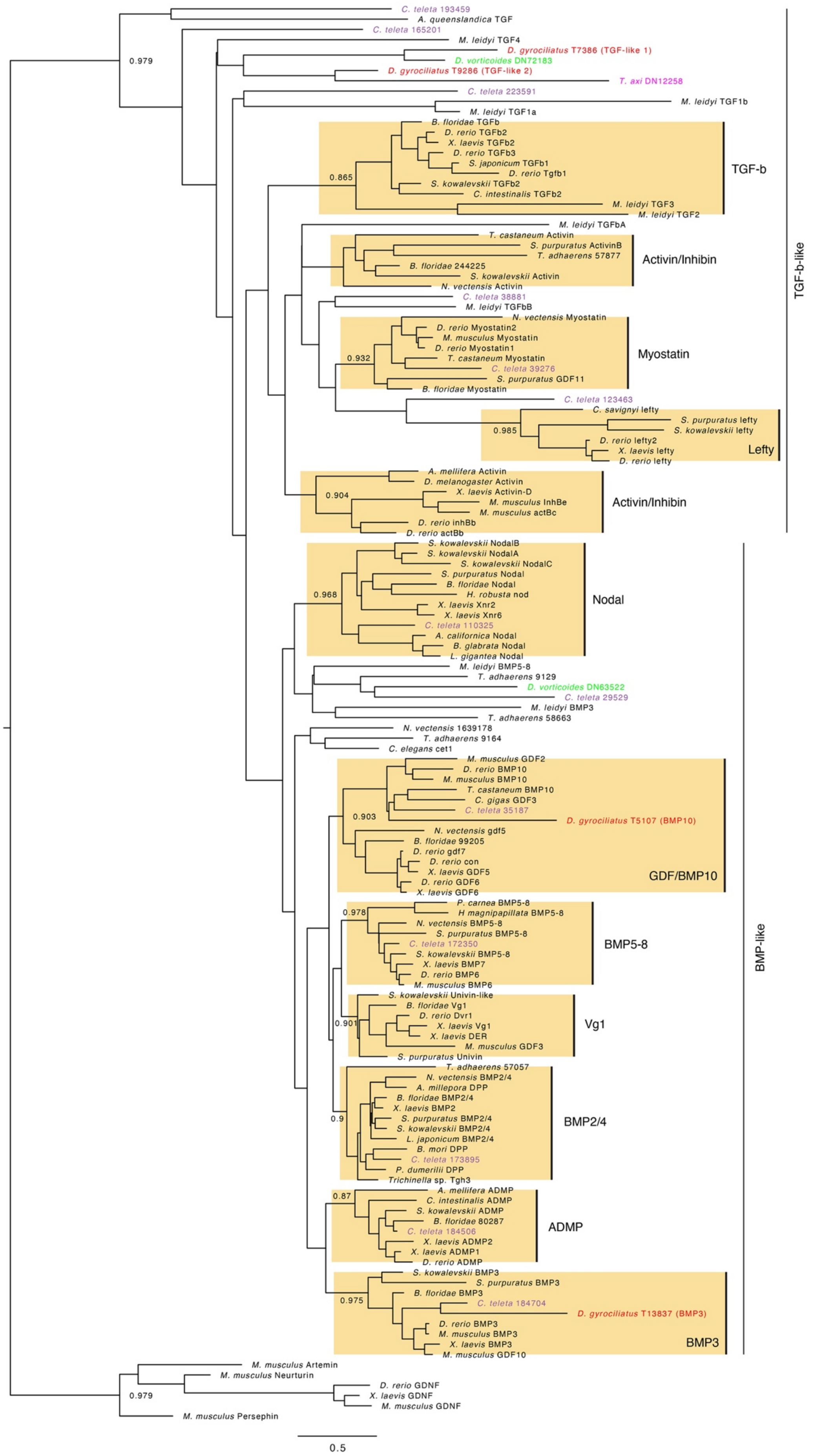

C. teleta 193459
A. queenslandica TGF
C. teleta 165201
M. leidyi TGF4
D. gyrociliatus T7386 (TGF-like 1)
D. vorticoides DN72183
D. gyrociliatus T9286 (TGF-like 2)
T. axi DN12258
C. teleta 223591
M. leidyi TGF1b
M. leidyi TGF1a
0.979
B. floridae TGFb
D. rerio TGFb2
X. laevis TGFb2
D. rerio TGFb3
S. japonicum TGFb1
D. rerio Tgfb1
S. kowalevskii TGFb2
C. intestinalis TGFb2
M. leidyi TGF3
M. leidyi TGF2
0.865
TGF-b
M. leidyi TGFbA
T. castaneum Activin
S. purpuratus ActivinB
T. adhaerens 57877
B. floridae 244225
S. kowalevskii Activin
N. vectensis Activin
Activin/Inhibin
C. teleta 38881
M. leidyi TGFbB
N. vectensis Myostatin
D. rerio Myostatin2
M. musculus Myostatin
D. rerio Myostatin1
T. castaneum Myostatin
C. teleta 39276
S. purpuratus GDF11
B. floridae Myostatin
0.932
Myostatin
C. teleta 123463
C. savignyi lefty
S. purpuratus lefty
S. kowalevskii lefty
D. rerio lefty2
X. laevis lefty
D. rerio lefty
0.985
Lefty
A. mellifera Activin
D. melanogaster Activin
X. laevis Activin-D
M. musculus InhBe
M. musculus actBc
D. rerio inhBb
D. rerio actBb
0.904
Activin/Inhibin
TGF-b-like
S. kowalevskii NodalB
S. kowalevskii NodalA
S. kowalevskii NodalC
S. purpuratus Nodal
B. floridae Nodal
H. robusta nod
X. laevis Xnr2
X. laevis Xnr6
C. teleta 110325
A. californica Nodal
B. glabrata Nodal
L. gigantea Nodal
0.968
Nodal
M. leidyi BMP5-8
T. adhaerens 9129
D. vorticoides DN63522
C. teleta 29529
M. leidyi BMP3
T. adhaerens 58663
N. vectensis 1639178
T. adhaerens 9164
C. elegans cet1
M. musculus GDF2
D. rerio BMP10
M. musculus BMP10
T. castaneum BMP10
C. gigas GDF3
C. teleta 35187
D. gyrociliatus T5107 (BMP10)
N. vectensis gdf5
B. floridae 99205
D. rerio gdf7
D. rerio con
X. laevis GDF5
D. rerio GDF6
X. laevis GDF6
0.903
GDF/BMP10
P. carnea BMP5-8
H magnipapillata BMP5-8
N. vectensis BMP5-8
S. purpuratus BMP5-8
C. teleta 172350
S. kowalevskii BMP5-8
X. laevis BMP7
D. rerio BMP6
M. musculus BMP6
0.978
BMP5-8
S. kowalevskii Univin-like
B. floridae Vg1
D. rerio Dvr1
X. laevis Vg1
X. laevis DER
M. musculus GDF3
S. purpuratus Univin
0.901
Vg1
T. adhaerens 57057
N. vectensis BMP2/4
A. millepora DPP
B. floridae BMP2/4
X. laevis BMP2
S. purpuratus BMP2/4
S. kowalevskii BMP2/4
L. japonicum BMP2/4
B. mori DPP
C. teleta 173895
P. dumerilii DPP
Trichinella sp. Tgh3
0.9
BMP2/4
A. mellifera ADMP
C. intestinalis ADMP
S. kowalevskii ADMP
B. floridae 80287
C. teleta 184506
X. laevis ADMP2
X. laevis ADMP1
D. rerio ADMP
0.87
ADMP
S. kowalevskii BMP3
S. purpuratus BMP3
B. floridae BMP3
C. teleta 184704
D. gyrociliatus T13837 (BMP3)
D. rerio BMP3
M. musculus BMP3
X. laevis BMP3
M. musculus GDF10
0.975
BMP3
BMP-like
M. musculus Artemin
M. musculus Neurturin
D. rerio GDNF
X. laevis GDNF
M. musculus GDNF
M. musculus Persephin
0.979
0.5

Supplementary Figure 6 | TGF-β ligand orthology assignment. Maximum likelihood tree of TGF-β ligands, including those putative sequences identified in *D. gyrociliatus* (in red), *T. axi* (in pink), *D. vorticoides* (in green) and *C. teleta* (in violet). Each major orthology group is highlighted with a light-yellow box, and only bootstrap values supporting these clades are shown.

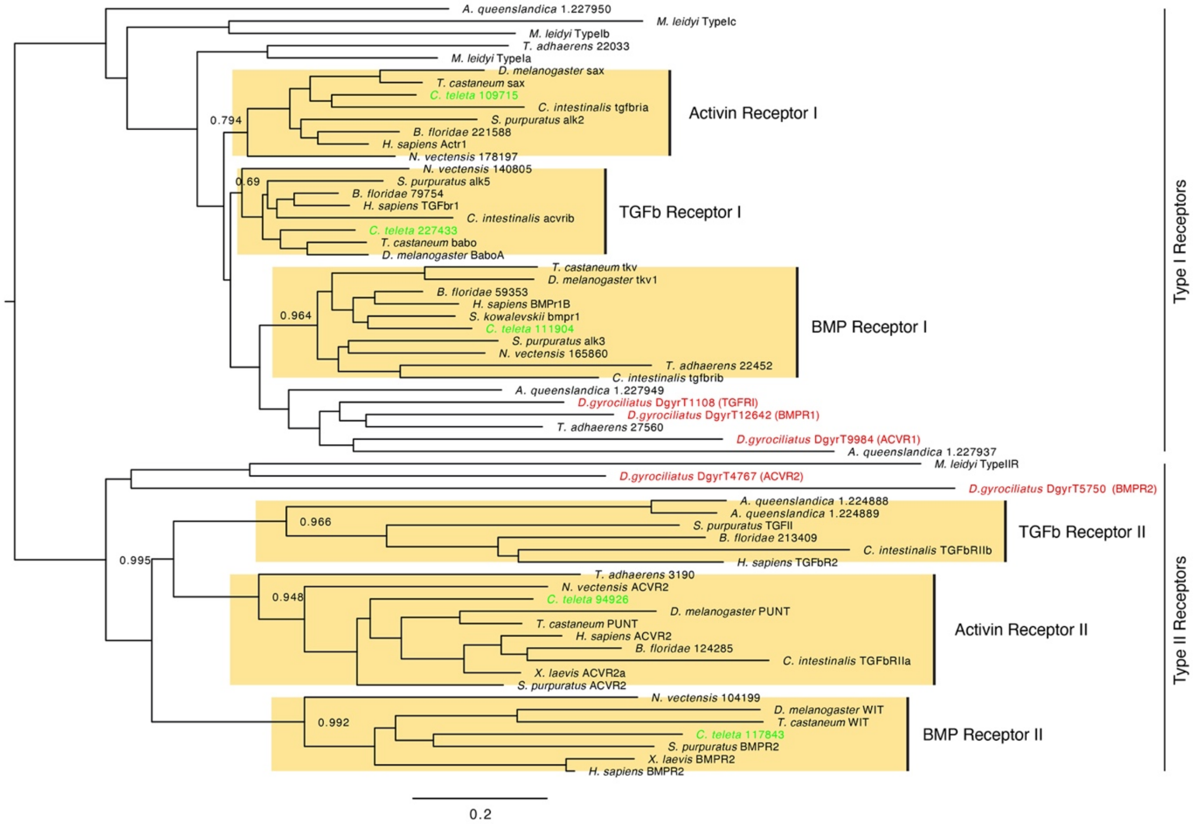

Supplementary Figure 7 | TGF-β receptor orthology assignment. Maximum likelihood tree of TGF-β receptors, including those putative sequences identified in *D. gyrociliatus* (in red) and *C. teleta* (in green). Each major orthology group is highlighted with a light-yellow box, and only bootstrap values supporting these clades are shown.

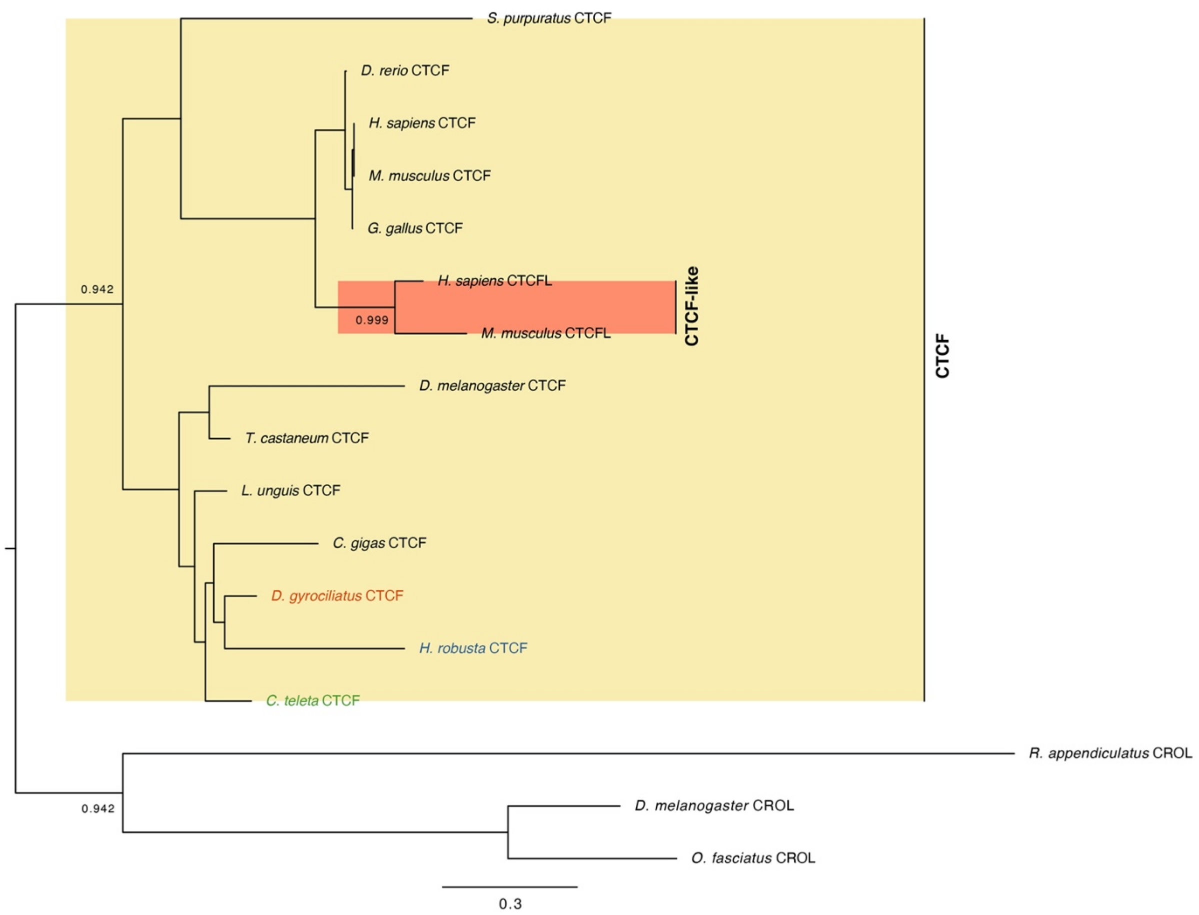

Supplementary Figure 8 | CTCF orthology assignment. Maximum likelihood tree of CTCF, including those putative sequences identified in *D. gyrociliatus* (in red), *C. teleta* (in green) and *H. robusta* (in blue). Crooked legs (CROL) zinc fingers are used as outgroup. CTCF orthology group is highlighted with a light-yellow box, and only bootstrap values supporting these clades are shown.

Supplementary Tables

Supplementary Table 1 | Genome assembly statistics.

	D. gyrociliatus	*T. axi*	*C. teleta*[a]	*H. robusta*[a]	*H. elegans*[b]	*E. fetida*[c]	*S. lamarcki*[d]	*L. luymesi*[e]
Genome size (Mbp)	77.9	120.2	333.72	235.34	1,026.05	1,052.63	1,501.63	687.7
GC (%)	31.36	26.77	40.39	32.82	35.43	40.87	34.78	40.16
# of scaffolds	349	169,751	21,042	1,993	188,407	1,659,527	2,474,717	11,871
# of scaffolds >1 kb	347	28,366	21,042	1,993	101,980	268,873	355,424	NA
Largest scaffold (Mb)	5.53	0.032	1.62	13.64	0.244	0.059	0.053	2.12
Scaffold N50 (Mb)	2.24	0.002	0.19	3.06	0.018	0.003	0.002	0.373
# N's per 100 kb	1,170.41	1,117.84	16,991.19	8,471.67	2,551.11	34,398.06	4,959.94	NA

[a]REF[50]; [b]REF[165]; [c]REF[166]; [d]REF[167]; [e]REF[168]

Supplementary Table 2 | Transcriptome assembly statistics.

	de novo	genome-guided
Total bases	200,773,339	196,572,502
Transcripts	87,192	74,131
Unique genes	49,945	39,432
Transcripts with ORF	44,889	42,041
Largest	34,371	30,551
Mean transcript length	2,303	2,652
Transcripts over 10kb	2,301	2,302
Transcripts over 1kb	47,905	45,625
N50	4,544	4,934
E90N50	4,017	4,229
GC	0.34	0.34
BUSCO (%)	96.4	95.8

Supplementary Table 3 | Automated annotation of repeats and transposable elements in annelid genomes.

	D. gyrociliatus	*Capitella teleta*[a]	*Helobdella robusta*[a]
Interspersed repeats[b]	7.95% (6.19 Mb)	22.87% (76.39 Mb)	18.46% (43.38 Mb)
Simple repeats	2.69% (2.09 Mb)	3.68% (12.3 Mb)	12.21% (28.69 Mb)
Low complexity	0.55% (0.43 Mb)	4.24% (14.15 Mb)	2.58% (6.07 Mb)
Total masked	11.18% (8.71 Mb)	31.06% (103 Mb)	33.39% (78 Mb)

[a]Percentages obtained from REF[50].

[b]Including unclassified

Supplementary Table 4 | Manually identified repeats and TEs in *Dimorphilus* genome.

Repeat class		Bases	% of assembly
	Gypsy-like	1298162	1,67
	Other LTR	178982	0,23
Total LTR		*1477144*	*1,90*
	Mariner	667512	0,85
	MULE	1167144	1,50
	Helitron	51842	0,07
Total DNA		*1886498*	*2,42*
	Penelope-like	340281	0,44
	non-LTR	29302	0,04
Total non-LTR		369583	0,47
	MITEs	60382	0,08
Total TEs		*3793607*	*4,87*
	rRNA	39378	0,05
	snRNA	5858	0,01
	tRNA	26258	0,03
	Low complexity	423366	0,54
	Simple repeats	2059306	2,64
	Protein Domain-derived	1034653	1,33
	Unknown	1365378	1,75

Supplementary Table 5 | Comparison of TE abundance and diversity in small animal genomes.

Genome	Assembly size, Mb	% of annotated TEs	TE superfamilies	Reference
Dimorphilus gyrociliatus	78	4.87	7	This study
Oikopleura dioica	72	14.10	9	REF[169]
Fritillaria borealis	91	14.40	9	REF[169]
Trichoplax adhaerens	98	0.13	16	REF[170]
Polypedilum nubifer	107	1.26	14	REF[171]
Polypedilum vanderplanki	104	0.26	6	REF[171]
Ramazzottius varieornatus	55	18.12	-	REF[35]
Belgica antarctica	99	0.12		REF[172]
Caenorhabditis elegans	100	12–16		REF[173]

Supplementary Table 6 | List of species and gene annotation files used in this study

Clade	Species	Source	Version
Non-bilaterian	*Nematostella vectensis*	EnsemblMetazoa	ASM20922v1
	Amphimedon queenslandica	EnsemblMetazoa	Aqu1
	Mnemiopsis leidyi	EnsemblMetazoa	MneLei_Aug2011
Deuterostomia	*Homo sapiens*	Ensembl	GRCh38
	Lepisosteus oculatus	Ensembl	LepOcu1
	Oikopleura dioica	OikoBase	reference v.1.0
	Ciona intestinalis	Ensembl	GCA_000224145.1
	Branchiostoma lanceolatum	Amphiencode	Bl71
	Saccoglossus kowalevskii	OIST	JGIv3.0
Ecdysozoa	*Ramazzottius varieornatus*	Kumamushi GP	Downloaded 04/09/18
	Strigamia maritima	EnsemblMetazoa	Smar1
	Drosophila melanogaster	EnsemblMetazoa	BDGP6
	Tribolium castaneum	EnsemblMetazoa	Tcas5.2
	Caenorhabditis elegans	EnsemblMetazoa	WBcel235
	Pristionchus pacificus	EnsemblMetazoa	WS220
Spiralia	*Capitella teleta*	EnsemblMetazoa	Capitella_teleta_v1.0
	Helobdella robusta	EnsemblMetazoa	Helro1
	Dimorphilus gyrociliatus	This study	Filtered gene models
	Hydroides elegans	NCBI	GCA_001703475.1
	Lottia gigantea	EnsemblMetazoa	Lotgi1
	Crassostrea gigas	EnsemblMetazoa	oyster_v9
	Octopus bimaculoides	EnsemblMetazoa	PRJNA270931
	Patinopecten yessoensis	PyBase	GCF_002113885.1
	Lingula anatina	OIST	v2
	Phoronis australis	OIST	ASM263300v1
	Notospermus geniculatus	OIST	ASM263302v1
	Macrostomum lignano	WormBase	ML2
	Schmidtea mediterranea	smedDB	sexual v4
	Adineta vaga	EnsemblMetazoa	AMS_PRJEB1171_v1

Supplementary Table 7 | Gene family statistics in annelid proteomes

	D. gyrociliatus	*C. teleta*	*H. robusta*
# genes	14204	32173	23432
# genes in orthogroups	12058	25271	16552
# genes in species-specific orthogroups	270	757	353
# shared orthogroups containing species	7277	9586	6388
# species-specific orthogroups	45	121	96
Average shared orthogroup size	1.63	2.59	2.57
% of genes in orthogroups with just one per-species gene	38.9	19	19.1

Supplementary Table 8 | Estimation of GPCRs in *D. gyrociliatus* and other annelid and bilaterian genomes[a].

	Class A (Rhodopsin)	Class B (Secretin & Adhesion)	Class C (Glutamate)	Class F (Frizzled)
D. gyrociliatus	211[b] (184)[c] [154][d]	16 (12) [12]	18 (18) [14]	5 (5) [4]
C. teleta	1058 (889) [343]	40 (34) [22]	51 (33) [23]	5 (3) [5]
H. robusta	229 (222) [165]	30 (28) [18]	11 (9) [10]	5 (5) [4]
L. anatina	492 (384) [197]	48 (33) [29]	44 (21) [20]	6 (5) [5]
L. gigantea	309 (296) [196]	59 (52) [32]	17 (12) [15]	5 (3) [5]
R. varieornatus	424 (363) [115]	24 (24) [17]	22 (22) [12]	6 (6) [5]
D. melanogaster	134 (73) [58]	33 (14) [8]	23 (9) [10]	8 (4) [5]
S. maritima	93 (88) [80]	37 (31) [22]	14 (11) [13]	5 (5) [5]
O. dioica	21 (21)	11 (11)	0	2 (2)

[a]The numbers shown in this table can generally differ between other publications as different thresholds are used to search for GPCRs and different thresholds are used to determine the different types.

[b]Number of GPCR sequences in protein database, including all splice variants.

[c](#): Number of different GPCR types using a similarity score of $p < 1e\text{-}150$, using the Network based cluster search of the CLANS software[174], with offset values and minimum sequences per cluster set to "1". The similarity value was chosen after testing the *Drosophila* class A GPCRs to distinguish between the two *D. melanogaster* CCHamide receptor paralogs as well as between the *D. melanogaster* octopamine beta 1, 2 and 3 receptors.

[d][#]: Number of different GPCR types based on monophyletic nodes. (Ignoring species-specific expansions and strongly dependent on taxon sampling)

Supplementary Table 9 | DNA double-strand break machinery in *D. gyrociliatus*

**Classical non-homologous end-joining**		**Homologous recombination**	
Mammalian gene	*D. gyrociliatus* ortholog	Mammalian gene	*D. gyrociliatus* ortholog
Ku70	DgyrG8781	Rad50	DgyrG4628
Ku80	DgyrG13008	Mre11	DgyrG7523
Artemis	DgyrG1840	Nbs1	DgyrG9305
DNAPKcs	DgyrG1700	RPA	DgyrG2509, DgyrG121
Polλ	DgyrG6710	Rad51	DgyrG9448
Polμ	DgyrG6571	Rad52	DgyrG9875
TdT	–	Rad54	DgyrG1112, DgyrG6388
Lig4	DgyrG11361	Polδ	DgyrG11456, DgyrG3541
XRCC4	DgyrG7932	BLM	Dgyr_TRINITY_GG_2260_c3_g1
XLF	DgyrG4118	Mus81	DgyrG7297
		TOP3	DgyrG5027, DgyrG10546
**Microhomology-mediated end joining**		Eme1	DgyrG6301
FEN1	DgyrG4375	ATM	DgyrG10839
MRE11	DgyrG7523	RBBP8	DgyrG9775
NBS1	DgyrG9305	BARD1	DgyrG6393
PARP1	DgyrG7212	BRCA1	Absent (present in *C. teleta*)
XRCC1	DgyrG10492	BRIP1	DgyrG8613
		TOPBP1	DgyrG3868, DgyrG9395
		Abraxas	DgyrG9679
		RAP80	Absent (also in *C. teleta*)
		BABAM1	DgyrG1474
		BRE	DgyrG1708
		BRCC36	DgyrG7090
		PALB2	Absent (also in *C. teleta*)
		BRCA2	DgyrG9400
		DSS1	DgyrG2531
		SYCP3	Absent (also in *C. teleta*)
		EXO1	DgyrG4997

Supplementary Table 10 | Hippo and PI3K/AKT/mTOR pathways in *D. gyrociliatus*

Hippo signalling pathway

Mammalian gene	*D. gyrociliatus* ortholog
DCHS1, DCHS2	DgyrG13180, DgyrG13181, DgyrG3311
FAT1, FAT2, FAT3, FAT4	DgyrG994, DgyrG1941, DgyrG2849, DgyrG3502, DgyrG3719, DgyrG3720, DgyrG3721, DgyrG3004, DgyrG5566, DgyrG9358, DgyrG12126, DgyrG12900, DgyrG13111
FRMD6	DgyrG10654
WWC1	DgyrG8366
NF2	DgyrG10287
MST1, MST2	DgyrG3859
SAV1	DgyrG7095
LATS1, LATS2	DgyrG2233
MOBKL1A, MOBKL1B	DgyrG13030, DgyrG5479
YAP, TAZ	DgyrG2050, DgyrG6616
TEAD1, TEAD2, TEAD3, TEAD4	DgyrG11620, DgyrG1665

Cyclins & CDKs

Cyclin A	DgyrG4878
Cyclin B	DgyrG3932, DgyrG5507, DgyrG8783, DgyrG11444
Cyclin D	DgyrG4062
Cyclin E	DgyrG5820
Cyclin H	DgyrG3506
CDK1	DgyrG10281, DgyrG12798, DgyrG3962, DgyrG5223
CDK2	DgyrG4938
CDK4/6	DgyrG4633
CDK7	DgyrG5300

PI3K/AKT (core elements, regulators, and main downstream targets)

Mammalian gene	*D. gyrociliatus* ortholog
PI3K	DgyrG10786
PDK1	DgyrG9699
AKT	DgyrG7512
PTEN	DgyrG11376
CREB	DgyrG7210
NFκB	DgyrG8989
p53/p63/p73	DgyrG10881, DgyrG6940
BCL-2	DgyrG571
FOXO	DgyrG22, DgyrG23
MYC	DgyrG1164
p21/p27	Absent (present in *C. teleta*)

mTOR complex 1 and 2 components

TSC1	DgyrG2631
TSC2	DgyrG671, DgyrG673
RHEB	DgyrG7989
RPTOR	DgyrG9654
PRAS40	Absent (also in *C. teleta*)
mTOR	DgyrG7813
DPTOR	DgyrG873
mLST8	DgyrG6166, DgyrG6534
TEL2	DgyrG11878
TTI1	DgyrG932
RICTR	DgyrG2479
PRR5, PRR5L	Absent (present in *C. teleta*)

	SIN1	DgyrG10819

Supplementary Table 11 | Presence/absence of MNT/MAD in Metazoa

Species	Genome Size (Gb)	MAD	MNT
A. queenslandica	0.167	Y	N
M. leidyi	0.15	Y	N
N. vectensis	0.34	Y	Y
S. kowalevskii	0.758	Y	Y
B. lanceolatum	0.59	Y	Y
C. intestinalis	0.2	Y	N
O. dioica	0.07	N	N
L. oculatus	1.4	Y	Y
H. sapiens	3.1	Y	Y
C. elegans	0.103	Y	N
P. pacificus	0.17	Y	N
R. varieornatus	0.055	N	Y
S. maritima	0.29	Y	Y
D. melanogaster	0.17	N	Y
T. castaneum	0.21	Y	Y
A. vaga	0.244	N	Y
M. lignano	0.742	Y	N
S. mediterranea	0.72	Y	N
O. bimaculoides	2.93	N	Y
L. gigantea	0.43	Y	Y
C. gigas	0.637	Y	Y
M. yessoensis	0.988	Y	Y
D. gyrociliatus	0.072	N	N
C. teleta	0.324	Y	Y
H. robusta	0.24	Y	N
N. geniculatus	0.859	Y	N
P. australis	0.498	Y	Y
L. anatina	0.41	Y	Y

Supplementary Notes

Supplementary Note 1: Background information on *Dimorphilus gyrociliatus*

The meiofaunal annelid family Dinophilidae Macalister, 1876, contains 18 species in three genera, which differ in their morphology and life cycle[44]. They are found worldwide in marine intertidal to subtidal waters, living interstitially in coarse sediments as well as epibenthically on biofilm and filamentous algae growing on macroalgae. All dinophilids are microscopic, 1–3 mm long worms, characterised by six body segments and dense midventral locomotory ciliation used for gliding. Their segments are not always externally discernible, and all species lack common annelid traits such as chaetae (chitinous bristles), parapodia (paired lateral segmental outgrows for locomotion), a blood vascular system and a (trochophore) larval stage. Furthermore, they possess protonephridia (not metanephridia) and are devoid of sensory, feeding, and respiratory appendages such as palps, antennae or cirri on both head and trunk[175].

Whereas the monophyly of the family is highly supported by both morphological and molecular data[16,176,177] *Dinophilus* O. Schmidt, 1848 was recently recovered paraphyletic with respect to *Trilobodrilus* Remane, 1925, causing the erection of a third monophyletic dinophilid genus *Dimorphilus* Worsaae, Kerbl, Vang & Gonzalez, 2019 (sister to *Dinophilus sensu stricto* and *Trilobodrilus*). This genus so far includes two dimorphic species, *D. gyrociliatus* (O. Schmidt, 1857) and *D. kincaidi* (Jones & Ferguson, 1957), which are set apart from other dinophilids by having dwarf males and a short life cycle[44]. *Dimorphilus gyrociliatus* was originally described from tidal zone macroalgae in Naples, Italy, but has since then been reported from public aquaria and tidal habitats around the world, though these records most likely represent several cryptic species[44]. The present study is based on laboratory cultures originally established by B. Åkesson (University of Gothenburg) from

collections of *D*. cf. *gyrociliatus*[178] in the 1980s in Xiamen, China and currently kept in the lab of K. Worsaae (University of Copenhagen, since 2006), A. Hejnol (University of Bergen, since 2014) and J. M. Martín-Durán (Queen Mary University of London, since 2018).

Dimorphilus gyrociliatus male and female morphology diverges vastly, starting with egg size: male eggs are approximately 40 µm and female eggs 80 µm in diameter[179]. The roughly 1 mm long adult females possess six segments and have a well-developed digestive, neural and muscular systems as well as segmentally arranged protonephridia, whereas the males are only 0.05 mm in length and lack a digestive tract. The nervous system is probably the best-studied organ system in both sexes: Females show an annelid-like nervous system with anterodorsal brain (with central neuropil and a surrounding layer of densely packed somata) and ventrolateral nerve cords, which are formed as one pair of ventrolateral nerve cords, two pairs of paramedian nerves and an unpaired ventromedian nerve, all connected by two to three transverse commissures per segment. Despite the male nervous system being reduced to only 68 neurons, it still comprises an anterior brain, two pairs of ventro-lateral nerves extending posteriorly, and a pair of posterior neuron clusters (penis ganglia) connected by a ventro-posterior commissure. No major differences are detected in size and overall shape of somata in male and female brains[48,180], although the neurons may differ slightly in morphology, e.g., in the branching topology of the axons (Kerbl *et al.*, unpublished). Patterns of genes characterising and demarcating the brain/anterior neural region of adult females reflect the arrangement and expression of orthologous genes in larvae of macroscopic annelids or other Spiralians, though the different stages and lifestyles included in the analyses complicate exact comparisons[47]. Remarkably, the (morphologically apparently similar) neurons differ in the specific neurotransmitters they synthetize, with neurons in the brains of adult females seemingly not producing two or more of the (so far tested) neurotransmitters

but being specific to one of them[48]. Surprisingly, there is a much higher overlap of individual neurotransmitter patterns in the nerves around the copulatory organ and penial ganglia of dwarf males[180], thereby suggesting that neurotransmitter profiles of individual cells might be related to size and complexity of animals.

Dimorphilus gyrociliatus has direct development and a fast life cycle with females laying eggs within three weeks of their life span and living only for a few months. The fertilised eggs are deposited in cocoons containing female and male eggs in an approximate ratio of 3:1. Female embryos hatch with five body segments and add the last segment in the days after hatching. Males have a life span of little more than a week, hatching short before their female siblings and fertilising primarily their sisters within the cocoon before this is ruptured. This also infers a putative high degree of inbreeding in *D. gyrociliatus*. The early development of females has been studied in detail[49]: it follows the general annelid pattern of spiral cleavage and formation of germ layers, and furthermore proposed homology of the second prostomial ciliary band to the prototroch in trochophore larvae of indirectly developing annelids.

Supplementary Note 2: Phylogenetic position of *D. gyrociliatus*: background

Since the discovery of *Dinophilus vorticoides* O. Schmidt, 1848, and thereby members of the family Dinophilidae, its relationship to and within Annelida has been debated. Whereas its initial ascription to Platyhelminthes (O. Schmidt 1848) was soon rejected and the relationship to annelids suggested by, among others, their embryonic development[49], the small and relatively simple body design and ciliated larval appearance of dinophilids have led to several speculations on their origin. Dinophilidae were originally regarded as ancestrally small "Archiannelida"[181-183], but were later considered to be secondarily miniaturised forms, possibly originating through progenesis (in Gould's sense[184], involving accelerated maturation and early offset of somatic growth in a larval/juvenile stage of a macrofaunal ancestor)[52,185,186]. The latter hypothesis, though hard to test, is founded not only by the small size and lack of adult annelid features, but also by the resemblance of adult dinophilids to larval or juvenile stages of macrofaunal annelids (especially within Eunicida). These morphological comparisons focused on the configuration of ciliary patterns, reduced coelomic cavities and protonephridial systems, low segment number as well as the uncondensed architecture of the ventrolateral nerve cords[45,176,186,187].

Larvae of some eunicidan families such as Dorvilleidae show strong resemblance to dinophilids in the overall shape of prostomium, body segments and pygidium as well as in the presence of segmental transverse ciliary bands. With the discovery of several heavily reduced meiofaunal genera of Dorvilleidae (e.g., *Apodotrocha* Westheide & Riser, 1983), this resemblance was strengthened and Dinophilidae was for a while depicted as highly derived dorvilleid, putatively having originated via a series of reduction events throughout the diversification of Dorvilleidae[188]. However, as also argued in several studies, the severe

reduction in dinophilid morphology could also be the outcome of only one or few progenetic events, thereby indicating the family derived from other macrofaunal Eunicida[187,189-191].

In contrast to these morphological considerations, early molecular phylogenetic studies[177,192] already defied the close relationship of Dinophilidae to Dorvilleidae and Eunicida, which was also not found when incorporating more data and taxa in later phylogenomic studies[52,53]. Andrade *et al.* (REF[53]) observed Dinophilidae to either nest within Sedentaria or as sister to Pleistoannelida. Since this equivocality seemed to reflect confounding long branch effects, the authors did not regard the family's position within Annelida as resolved. Struck *et al.* (REF[52]) addressed a different data set and suggested Dinophilidae to be placed with Orbiniida within Sedentaria (as sister group to Nerillidae), but again within a clade containing several long branches. David & Halanych (REF[193]) placed Dinophilidae in Sedentaria based on mitogenomic analyses of seven pleistoannelids, but remarkably, the dinophilid mitochondrial gene order (otherwise regarded to be a highly conserved gene order) differed from that of the other analysed species.

Herein, we find Dinophilidae to group with Lobatocerebridae, the two of them (=Dinophiliformia) constituting the sister group to Pleistoannelida in all analyses, except when using a simpler site-homogenous model (LG4X + R), which is instead inferring a relationship to Orbiniida within Sedentaria. Either position still supports Dinophilidae as secondarily reduced in size and complexity, yet this reduction may have occurred already in the origin of the common ancestor of Dinophiliformia to some degree, since the meiofaunal family Lobatocerebridae also exhibits reduced morphological complexity and size[194]. None of the former analyses of Dinophilidae's position included Lobatocerebridae, which was only recently phylogenomically established as an annelid family[195]. This sister-family relationship

was here found consistently and independent of the inclusion or exclusion of some of the other long branched taxa which Dinophilidae had previously shown affinity to, such as *Osedax*[53]. One may still argue that Lobatocerebridae also constitutes a long branch and their close relationship to Dinophilidae as well as the entire clade's position next to Pleistoannelida are somehow affected by confounding long branch attraction effects. However, Dinophilidae is consequently placed outside Pleistoannelida throughout all analyses employing site-heterogenous models and with different taxon sampling. Interestingly, Dinophilidae do show some morphological resemblance to Lobatocerebridae besides them both being meiofaunal, e.g., in the common presence of highly separated ventral nerve cords and an unpaired median nerve as well as in a diverse set of mainly epidermal glands[194]. Still, Lobatocerebridae's elongated, entirely ciliated body, multilobed brain, lack of apparent segmentation and different reproductive system differs starkly from Dinophilidae.

Supplementary References

165 Shikuma, N. J., Antoshechkin, I., Medeiros, J. M., Pilhofer, M. & Newman, D. K. Stepwise metamorphosis of the tubeworm *Hydroides elegans* is mediated by a bacterial inducer and MAPK signaling. *Proc Natl Acad Sci U S A* **113**, 10097-10102 (2016).

166 Zwarycz, A. S., Nossa, C. W., Putnam, N. H. & Ryan, J. F. Timing and Scope of Genomic Expansion within Annelida: Evidence from Homeoboxes in the Genome of the Earthworm Eisenia fetida. *Genome Biology and Evolution* **8**, 271-281 (2015).

167 Kenny, N. J., Namigai, E. K., Marletaz, F., Hui, J. H. & Shimeld, S. M. Draft genome assemblies and predicted microRNA complements of the intertidal lophotrochozoans *Patella vulgata* (Mollusca, Patellogastropoda) and *Spirobranchus* (*Pomatoceros*) *lamarcki* (Annelida, Serpulida). *Mar Genomics* **24 Pt 2**, 139-146 (2015).

168 Li, Y. *et al.* Genomic adaptations to chemosymbiosis in the deep-sea seep-dwelling tubeworm *Lamellibrachia luymesi*. *BMC Biol* **17**, 91 (2019).

169 Naville, M. *et al.* Massive Changes of Genome Size Driven by Expansions of Non-autonomous Transposable Elements. *Curr Biol* **29**, 1161-1168 e1166 (2019).

170 Wang, S., Zhang, L., Meyer, E. & Bao, Z. Genome-wide analysis of transposable elements and tandem repeats in the compact placozoan genome. *Biol Direct* **5**, 18 (2010).

171 Gusev, O. *et al.* Comparative genome sequencing reveals genomic signature of extreme desiccation tolerance in the anhydrobiotic midge. *Nat Commun* **5**, 4784 (2014).

172 Kelley, J. L. *et al.* Compact genome of the Antarctic midge is likely an adaptation to an extreme environment. *Nat Commun* **5**, 4611 (2014).

173 Laricchia, K. M., Zdraljevic, S., Cook, D. E. & Andersen, E. C. Natural Variation in the Distribution and Abundance of Transposable Elements Across the *Caenorhabditis elegans* Species. *Mol Biol Evol* **34**, 2187-2202 (2017).

174 Frickey, T. & Lupas, A. CLANS: a Java application for visualizing protein families based on pairwise similarity. *Bioinformatics* **20**, 3702-3704, doi:10.1093/bioinformatics/bth444 (2004).

175 Westheide, W. & Riser, N. W. Morphology and phylogenetic relationships of the neotenic interstitial polychaete *Apodotrocha progenerans* n.gen., n.sp. (Annelida). *Zoomorphology* **103**, 67-87 (1983).

176 Müller, M. C. M. & Westheide, W. Comparative analysis of the nervous systems in presumptive progenetic dinophilid and dorvilleid polychaetes (Annelida) by immunohistochemistry and cLSM. *Acta Zoologica* **83**, 33-48 (2002).

177 Struck, T. H., Westheide, W. & Purschke, G. Progenesis in Eunicida ("Polychaeta," Annelida)--separate evolutionary events? Evidence from molecular data. *Mol Phylogenet Evol* **25**, 190-199 (2002).

178 Wu, B. L. & Chen, M. On archiannelids of the Yellow Sea II: Dinophilidae and Nerillidae. *Oceanol Sin* **2**, 90-97 (1980).

179 Traut, W. Eine Mutante mit vergrößerten Männchen-Eiern bei Dinophilus gyrociliatus (Archiannelida). *Experientia* **22**, 237-238 (1966).

180 Kerbl, A., Winther Tolstrup, E. & Worsaae, K. Nerves innervating copulatory organs show common FMRFamide, FVRIamide, MIP and serotonin immunoreactivity patterns across Dinophilidae (Annelida) indicating their conserved role in copulatory behaviour. *BMC Zoology* **4**, 8 (2019).

181 Hatschek, B. Studie über die Entwicklungsgeschichte der Anneliden. Ein Beitrag zur Morphologie der Bilaterien. *Arb Zool Inst Univ Wien Zool Stat Triest* **1**, 277-404 (1878).

182 Hatschek, B. System der Anneliden, ein vorläufiger Bericht. *Lotos* **13**, 123-126 (1893).

183 Bubko, O. V. On systematic position of Oweniidae and Archiannelida (Annelida). *Zool Zhur* **52**, 1286-1296 (1973).

184 Gould, S. J. *Ontogeny and phylogeny*. (The Belknap Press of Harvard University Press, 1977).

185 Westheide, W. Progenesis as a principle in meiofauna evolution. *J. Nat. Hist.* **21**, 843-854 (1987).

186 Worsaae, K. & Kristensen, R. M. Evolution of interstitial Polychaeta (Annelida). *Hydrobiol* **535-536**, 319-340 (2005).

187 Westheide, W. in *The origin and relationships of lower invertebrates* (eds S. Conway Morris, J. D. George, R. Gibson, & H. M. Platt) 310-326 (Oxford University Press, 1985).

188 Eibye-Jacobsen, D. & Kristensen, R. M. A new genus and species of Dorvilleidae (Annelida, Polychaeta) from Bermuda, with a phylogenetic analysis of Dorvilleidae, Iphitimidae and Dinophilidae. *Zoologica Scripta* **23**, 107-131 (1994).

189 Westheide, W. *Ikosipodus carolensis* gen. et sp. n., an interstitial neotenic polychaete from North Carolina, U.S.A., and its phylogenetic relationship within Dorvilleidae. *Zool Scripta* **11**, 117-126 (1982).

190 Struck, T. H. Progenetic species in polychaetes (Annelida) and problems assessing their phylogenetic affiliation. *Integr Comp Biol* **46**, 558-568 (2006).

191 Purschke, G. in *Reproductive Biology and Phylogeny of Annelida* (eds G. Rouse & F. Pleijel) 639-667 (Science Publishers, 2006).

192 Struck, T. H., Halanych, K. M. & Purschke, G. Dinophilidae (Annelida) is most likely not a progenetic Eunicida: evidence from 18S and 28S rDNA. *Mol Phylogenet Evol* **37**, 619-623 (2005).

193 David, K. T. & Halanych, K. M. Mitochondrial genome of *Dinophilus gyrociliatus* (Annelida: Dinophilidae). *Mitochondrial DNA Part B* **2**, 831-832 (2017).

194 Kerbl, A., Bekkouche, N., Sterrer, W. & Worsaae, K. Detailed reconstruction of the nervous and muscular system of Lobatocerebridae with an evaluation of its annelid affinity. *BMC Evol Biol* **15**, 277 (2015).

195 Laumer, C. E. *et al.* Spiralian phylogeny informs the evolution of microscopic lineages. *Curr Biol* **25**, 2000-2006 (2015).
