# Supplementary material for: Conservative route to genome compaction in a miniature annelid
Source: Nat Ecol Evol. 2020 Nov 16;5(2):231–42. doi: 10.1038/s41559-020-01327-6 (PMC7854359; doi:10.1038/s41559-020-01327-6)
Supplement: Supplementary file 2 — Reporting Summary [file 41559_2020_1327_MOESM2_ESM.pdf]

nature research

Corresponding author(s): Jose M Martin-Duran
Andreas Hejnol

Last updated by author(s): Apr 27, 2020

Reporting Summary

Nature Research wishes to improve the reproducibility of the work that we publish. This form provides structure for consistency and transparency in reporting. For further information on Nature Research policies, see Authors & Referees and the Editorial Policy Checklist.

Statistics

For all statistical analyses, confirm that the following items are present in the figure legend, table legend, main text, or Methods section.

n/a	Confirmed	
☐	☒	The exact sample size (*n*) for each experimental group/condition, given as a discrete number and unit of measurement
☐	☒	A statement on whether measurements were taken from distinct samples or whether the same sample was measured repeatedly
☐	☒	The statistical test(s) used AND whether they are one- or two-sided<br>*Only common tests should be described solely by name; describe more complex techniques in the Methods section.*
☐	☒	A description of all covariates tested
☐	☒	A description of any assumptions or corrections, such as tests of normality and adjustment for multiple comparisons
☐	☒	A full description of the statistical parameters including central tendency (e.g. means) or other basic estimates (e.g. regression coefficient) AND variation (e.g. standard deviation) or associated estimates of uncertainty (e.g. confidence intervals)
☐	☒	For null hypothesis testing, the test statistic (e.g. *F*, *t*, *r*) with confidence intervals, effect sizes, degrees of freedom and *P* value noted<br>*Give P values as exact values whenever suitable.*
☐	☒	For Bayesian analysis, information on the choice of priors and Markov chain Monte Carlo settings
☒	☐	For hierarchical and complex designs, identification of the appropriate level for tests and full reporting of outcomes
☐	☒	Estimates of effect sizes (e.g. Cohen's *d*, Pearson's *r*), indicating how they were calculated

Our web collection on statistics for biologists contains articles on many of the points above.

Software and code

Policy information about availability of computer code

Data collection	No software was used to collect data for this study
Data analysis	We used the following open source software in this study: SMRTAnalysis (v.2.3.0.140936), PBcR (v.8.3rc2), Blobtools (v.0.9.16), Pilon (v.1.16), cutadapt (v.1.4.2), HaploMerger2 (v.20151124), SSPACE-LongRead (v.1.1), trimmomatic (v.0.35), SPAdes (v.3.6.2), Super_Deduper (v.2.0), Platanus (v.1.2.4), Quast (v.3.1), BWA-MEM (v.0.7.12-r1044), SAMtools (v.1.3.1), BUSCO (v2), BLAT (v.36x2), Isoblat (v.0.3), Picard tools (v.2.0.1), Jellyfish (v.2.2.3), Trinity (v2.1.1), Bowtie2 (v.2.1.0), CD-HIT (v.4.6), CAP3 (v.02/10/15), Kallisto (v0.44.0), DESeq2, Transdecoder (v5.0.2), HMMER (v.2.3.2), MSAProbs (v0.9.7), BGME, IQTREE, RepeatModeler (v.1.0.4), MITE Digger, MODELLER, CEGMA (v.2.4), AUGUSTUS (v.3.2.1), EXONERATE (v.2.2.0), PASA (v.2.0.2), GMAP (v.2015-12-31), EvidenceModeler (v.1.1.1), ORFik, BLAST (v.2.2.31+), Trinotate (v.3.0), signalP (v.4.1), tmHMM (v.2.0c), MAFFT (v7.310), Malin, OrthoFinder (v.2.2.7), FastTree (v.2), Clustal X, trimAl, MacVector (v.11.0.4), Adobe Photoshop CS6, Adobe Photoshop CC (v.14.0), Adobe Illustrator CC (v.17.0.0), MACS2 (v.2.1.1.20160309), IDRCode, HOMER (v2), CAGEr (v.1.20.0), KAT, GenomeScope2.0, BBTools

For manuscripts utilizing custom algorithms or software that are central to the research but not yet described in published literature, software must be made available to editors/reviewers. We strongly encourage code deposition in a community repository (e.g. GitHub). See the Nature Research guidelines for submitting code & software for further information.

Data

Policy information about availability of data

All manuscripts must include a data availability statement. This statement should provide the following information, where applicable:

- Accession codes, unique identifiers, or web links for publicly available datasets
- A list of figures that have associated raw data
- A description of any restrictions on data availability

All new raw sequence data associated to this project are available under project with primary accession PRJEB37657 in the European Nucleotide Archive (ENA). Genome annotation files and additional datasets are available in https://github.com/ChemaMD/DimorphilusGenome.

Field-specific reporting

Please select the one below that is the best fit for your research. If you are not sure, read the appropriate sections before making your selection.

☒ Life sciences ☐ Behavioural & social sciences ☐ Ecological, evolutionary & environmental sciences

For a reference copy of the document with all sections, see nature.com/documents/nr-reporting-summary-flat.pdf

Life sciences study design

All studies must disclose on these points even when the disclosure is negative.

Sample size	Sample sizes for genomic, transcriptomic and CAGE-seq analyses were estimated based on the amount of genomic DNA and total RNA obtained per individual. For ATAC-seq analyses, sample size was estimated in order to obtain a final number of 50,000 nuclei for subsequent tagmentation.
Data exclusions	No data was excluded from the analyses.
Replication	All RNA-seq, ATAC-seq and CAGE-seq analyses were conducted in replicates.
Randomization	All animal collections were performed randomly.
Blinding	All animal collections were allocated blindly to any of the replicates of study.

Reporting for specific materials, systems and methods

We require information from authors about some types of materials, experimental systems and methods used in many studies. Here, indicate whether each material, system or method listed is relevant to your study. If you are not sure if a list item applies to your research, read the appropriate section before selecting a response.

Materials & experimental systems

n/a	Involved in the study	
☒	☐	Antibodies
☒	☐	Eukaryotic cell lines
☒	☐	Palaeontology
☐	☒	Animals and other organisms
☒	☐	Human research participants
☒	☐	Clinical data

Methods

n/a	Involved in the study	
☒	☐	ChIP-seq
☐	☒	Flow cytometry
☒	☐	MRI-based neuroimaging

Animals and other organisms

Policy information about studies involving animals; ARRIVE guidelines recommended for reporting animal research

Laboratory animals	This manuscript uses the laboratory strains of the annelid species Dimorphilus gyrociliatus and Capitella teleta. For D. gyrociliatus, we used adult females and stage-specific embryonic samples. For C. teleta, we studied larval stages.
Wild animals	This study does not involve wild animals.
Field-collected samples	This manuscript studies the annelid species Trilobodrilus axi, which was collected from the wild by the lab of Katrine Worsaae. Adult specimens were kept in filtered seawater (31 ppm) at 15 ºC in the dark prior collection for genomic DNA extraction.
Ethics oversight	Work on annelid embryos are not subject of ethical approvals or restrictions.

Note that full information on the approval of the study protocol must also be provided in the manuscript.

Flow Cytometry

Plots

Confirm that:

☒ The axis labels state the marker and fluorochrome used (e.g. CD4-FITC).

☒ The axis scales are clearly visible. Include numbers along axes only for bottom left plot of group (a 'group' is an analysis of identical markers).

☒ All plots are contour plots with outliers or pseudocolor plots.

☒ A numerical value for number of cells or percentage (with statistics) is provided.

Methodology

Sample preparation	Hundreds of adult females of D. gyrociliatus were collected and starved for 3–4 days before flow cytometry analysis. Worms were transferred into a petri dish, washed well in seawater to remove any contaminant, and finely chopped with a razor blade in 2 ml of General-Purpose Buffer to generate a suspension of nuclei. This suspension was filtered through a 30 µm nylon mesh and stained with propidium iodide (Sigma; 1 mg/mL) on ice.
Instrument	We used a flow cytometer Partec CyFlow Space fitted with a Cobalt Samba green laser (532nm, 100mW)
Software	We used the built-in instrument software FloMax
Cell population abundance	We used flow cytometry to estimate genome size using C. elegans as reference and thus we did not sort any cell populations. To estimate genome size from propidium iodide staining, we did three independent runs for each species analysing at least 1,000 nuclei per run.
Gating strategy	We considered all cell populations for genome size estimation, and thus no gating strategy was implemented.

☒ Tick this box to confirm that a figure exemplifying the gating strategy is provided in the Supplementary Information.
