# Supplementary material for: Conservative route to genome compaction in a miniature annelid
Source: Nat Ecol Evol. 2020 Nov 16;5(2):231–42. doi: 10.1038/s41559-020-01327-6 (PMC7854359; doi:10.1038/s41559-020-01327-6)
Supplement: Supplementary file 5 — List of D. gyrociliatus neuropeptide precursors in fasta format. [file 41559_2020_1327_MOESM5_ESM.docx]

**#7B2**

>DgyrP13078 TRINITY_GG_5148_c0_g1_i2 Locus_18214.4_Transcript_1 7B2/Secregtogranin 5 isoform 1(*ppa 7B2)

MRFFNIFLVLVSLCLHQVLGDYSDLFYDDDLPSYSFNLPSELREEALKQYLEMVSRHNEDDIRVGSRDSTAGTEQRDEEHMEEGALGVQHPQFTSGGAGEGDQRLKPNGSIKNKNEVKSDAGLPFYCHPPNPCPKGFDPKKHNCDAHVRDVDKDQMAYIQKEMKDEKCTCDTDHMFTCPQNKNENAATDLASLLGENRHEGLIAKKSPSMQLHNQFFAETMNDA*

>DgyrP13079 TRINITY_GG_5148_c0_g1_i4 7B2/Secretogranin 5 isoform 2

MRFFNIFLVLVSLCLHQVLGDYSDLFYDDDLPSYSFNLPSELREEALKQYLEMVSRHNEDDIRVGSRDSTAGTEQRDEEHMEEGALGVQHPQFTSGGAGEGDQRLKPNGSIKNKNEVKSDAGLPFYCHPPNPCPKGFDPKKHNCDAHVRDVDKDQMAYIQKEMKDEKCTCDTDHMFTCPQSQFNPLMDFLYIKCISISCR*

**#Bursicon alpha**

>TRINITY_GG_14280_c4_g4_i6 Locus_15557.0_Transcript_1_extended Bursicon alpha (*ppa Bursicon alpha)

MFSKKILFYAFYILLYASLLDGQRQSSNSCRPKRIIYTIKVSKCQIKKTLLTACSGKCRSYTELNMANKTYDSYCNCCVPTKIETKKLMIRCYDEELKEIRIKWAKIKWPKSCSCRPCF*

**#Bursicon beta**

>Locus_9061.0_Transcript_1 TRINITY_GG_1435_c0_g1_i1_extended Bursicon beta (*ppa Bursicon beta)

MKAVNLYLFIAIAGYMINTVTSDCLITPSERTFKKEVTIRLPDNRWKRITCAASAKVNKCEGYCFSQATPSIIYPDFKKTCKCCKTLKSRERKIELDVCDDFELSIFEKKPIINFYEPVECQCIQCF*

**#Glycoprotein hormone alpha related**

>TRINITY_GG_3098_c0_g1_i1 Glycoprotein hormone alpha

MMKVLYIVFILLPFTFSQKAWQKVGCHRLGHTRLIEIGGCVRFRVQLNACRGHCESYTLPIPFNSPNSKTEKLESKASCCTALQTEDVYMTTWCHNGLRKFRFTSATVCQCSMCKGELW*

**#Glycoprotein hormone beta related**

>DgyrP1624 TRINITY_GG_3098_c1_g1_i1 4 Locus_13018.0_Transcript_1 Glycoprotein hormone beta

MLTPPQIEFIGIEDTFVEAMTLLWTKLFLLLAIFYMVKADEKSRKGTEFGCFNSSHKMNLNKDLKINATSVLKCRHTIQVPICWGRCDTWDIPDFQWNHSVCSYGRKTLKKVKLLDCDPNHPDPFMYLYVADTCECKLCKGIGCNYLHT*

**#ILP 1**

>CAP3_Contig2364 Locus_11072.0_Transcript_1 Insulin like peptide 1 (*ppa Insulin related peptide 3/5)

MKTKQQALVLTTALLLLLPFYIDAGYEHSCTFHQKLTNQNYICGDNLKTMLQTVCRMVGRKKRSVERSEKVFHPKKRALAMLNKRQTAGNQGIVCECCVNKCSLNEMAQYC*

**#ILP 2**

>TRINITY_GG_18782_c0_g1_i1 Locus_16843.0_Transcript_1 Insulin like peptide 2 (*ppa Insulin related peptide 2/4)

MITKNIVLILLLPIITLASEHICKIHEIHSMRQQKDCFGMIDKTIRTICGPGNVNGKRSDGSFIKKRTALAMLNTKRGRGDSIACECCVHQCDVKEWLSYCADPSRALVHRN*

**#ILP 3**

>CAP3_Contig1615 Locus_15736.1_Transcript_1 Insulin like peptide 3

MIGIKLFLLFLPMIALAYEQKCTVADILSVEQRVDCSKMIASTLNTICGPNNVSKRSNVHFIKKSNAFALLKAERKGSITCECCVHKCNTKEWLTYCKNPNEANISFLK*

**#ILP 4**

>TRINITY_GG_15450_c0_g1_i1 Locus_21619.0_Transcript_1 Insulin like peptide 4

MLFLYIILLSSYSTLSLRIRSQEEWRTSWHTECHRKCGRSLVQYVKMACANDIYKLPFKRSQTDNYALPRTQALSFLSKRKKRGITAECCRNKGCTWEELGEVCYSHPRSTPSIKATADCTFNNIK*

**#ILP 5**

>TRINITY_GG_10739_c0_g1_i1 Insulin like peptide 5

MNHNKILLVILSVLLMHSQSVTISPRLYQQIRSCGDELASHLEEVCLIAQREHFAYQKEKRPLIKSYFHIIDKREIRDDIISRCCRQSCYKFELMKFCH*

**#Prohormone_3**

>TRINITY_GG_289_c5_g1_i1_m3704 DgyrP12820_extended Locus_14783.1_Transcript_11 Prohormone 3 (ppa* Prohormone3)

MEKLQLYIFYFLVVLSCVGASSDRRRRGRCGGAGDKCYATRRCCSGYVCAAFDEFIDNTEDDSHPESPGNCMKEKDLNPCSDSGECGPGVRCVPLGPNREKYCLARYPSQHHSHVPETGRKSGDIGADCETDDDCKDTTDENDRLCCQVVTFFRRKPRKMCDRIHYDTKCIL*

**#Achatin**

>TRINITY_GG_250_c0_g1_i1 DgyrP12689 Achatin

MFHHLLASIVLLLCLSTPVFSAAVKRGFGEKRSFSDFGGDVFNKKWSATDFGSKMFNKRWSAQDFGNGMFNKKWSAEDFGSPIFQKREWADYLNQIYNKRGFGEKRSLQDFGSNFFNKRKDG*

**#Allatostatin_A**

>TRINITY_GG_19145_c3_g1_i10 DgyrP4866_extended Locus_13673.0_Transcript_1 Allatostatin A (*ppa Allatostatin A)

MCSAMRIAFISFCLTVSAVLAEEKKSDLESPFKDTEKRMDSAMRFMAFGKRLDPAMKLSGFGKKMDPAMKFAGFGKRMDPAMKFAGFGKRMDAAMKFAGFGKRMDAAMKFAGFGKRRDRRSVVEEDGYLELIKRKILEDLEDNDLAKRAYKRPYPRYIHFQRRYSIDPAYALLGLGK*

**#Allatostatin B/MIP**

>Locus_11964.0_Transcript_1 Allatostatin B/MIP (*ppa MIP)

MAYQTIICLPALLLLLLSATFAEKRQWSRDKYHITWGKRSVDGADDKREWSSNNMAMWGKRGWGGNKGMSMWGKRNDEAKADADKREWSDKTMALWGKRGVKDEEEKRSWNSKQMAMWGKRGWAAKNPALWGKRGWGQGTMWGKRDAEKV*

**#Allatostatin_C 1**

>Locus_18845.0_Transcript_1 Allatostatin C 1 (*ppa Allatostatin C)

EFEKLFMRARERETSTTTHIEISLLCVGCIYKTIGLYKGSSTSLRVHCFSFSDTCTMKFELAIFIVSSLLINCLISGESRLGEENYKVASLLQKALKADLERQYMHALDLERSLNTHLDRLREHKRQLEVRKRNPIQCLVNIVSCWKK*

**#Allatostatin_C 2**

>Locus_20442.0_Transcript_1 Allatostatin C 2

MRFGAFLIAFCLLYNVAFTVPIGGTKQMKTRDILENILRSYEESIAERNDLKSPQLIRKRDPYPCLVNIVACWQRRK*

**#Allatotropin**

>CAP3_Contig1154 Locus_19106.0_Transcript_1 Allatotropin (*ppa Allatotropin)

MKLTLIVTLMVITIATAYGLQFKERRGFRLGASDRFSHGFGKRADLFEQEIVLSVNKLSEKLANNPQFARLFLDRVIDRNGDGLITKSELLRDDSEKK*

**#Calcitonin-like 1**

>Locus_9944.0_Transcript_1 Calcitonin-like 1(*ppa Calcitonin)

MKSILFIFAALLIGVLFADKTKEEALGALKQMDESRRGIGALRMVMDEIETDISRQNEKISTVKRKQKALGLIRKLLSEMDNDLISEQKRTCVVNLGGHCSTEHAAAVAQQWHYLNSAMSPGRKRRDTRMFKKLVDGSLIREVD*

**#Calcitonin-like 2**

>DgyrP10954 Calcitonin-like 2

MKSILFIFAALLIGVLFADKTKEEALGALKQMDESRRGIGALRMVMDEIETDISRQKRNPCHIGLGNTYQCALATLENEPGVNVNGPNSPGKRRFLNNSQNP*

**#CCWamide/Agatoxin-like**

>Locus_18800.0_Transcript_1 CCWamide-like (*ppa CCWamide)

MLKTIIFILAASYLLSDAASSADTKRDSEDLGRLLKRIRTAKRQNWRDLFCGSANEWCDDEGTSCCSGYSCKCNIWNTNCRCVEKLFG*

**#Cerebrin/PDF 1**

>Locus_18742.0_Transcript_1 Cerebrin/PDF 1

MMPLKLLVVLLGFLLLADTTPIKRDTSVSDFAEELSSVGRKLISLAEKIKQNGGEKAIKRNYGTLDALIGMPDFFHQGKKR*

**#Cerebrin/PDF 2**

>Locus_18681.0_Transcript_1 DgyrP16258 DgyrP16259 Cerebrin/PDF 2

MAYVIRSLFLIVLLVNGLFVNAENTQSKMVDGFSPKTALQLAAIGRRLLRIAAEMRGDVDAKKRNSGMLDALINIPDFFNSGRR*

**#DH31-like 1**

>DgyrP15681 TRINITY_GG_3353_c0_g1_i2 Locus_16870.0_Transcript_1 DH31-like 1

MNRKLSMKALWLLAACAVLFTIVIETRAFDRNANDYNDVVDALRKIYLNRAMTKRRLDMGYGNRFDLAASIGSKLMALKHANDLSGPGRK*

**#DH31-like 2**

>Locus_13284.0_Transcript_1 DH31-like 2 (C-terminus identical to P.dumerilii DH31, but strongly extended N-terminus)

MNGRLLIYLYGCWLVILIRPSTMVESLKKDGTNTILKDIVKEIEMHYMKLRSPSSVGAKLFALKQAADWNGPGKRSWNNPEEIYYEPECRCKK*

**#DLamide**

>DgyrP4122 TRINITY_GG_17063_c0_g1_i1 Locus_9360.0_Transcript_2 (*ppa DLamide)

MAKDYSACLLIILLWSSSLADAVKTEFDDVWDPSYHRDQPALAQYKRFMFNPDLGKRMFNMDLGKRMFNNDLGKRMFNMDLGKKKKRENN*

**#Excitatory peptide**

>TRINITY_GG_10972_c1_g1_i3 Locus_19066.0_Transcript_1 Excitatory peptide (*ppa Excitatory peptide)

MERLRLLWIFISVLGTLSFLAEGGKCPVWALHACAGGNGKRSEATNERESGLRQIINQLRSLQRENFEKPRKFTSDFPFFEKFLRKSEKGDSIWDEKRNVFRNF*

**#FMRFamide-like 1**

>TRINITY_GG_12864_c0_g3_i1 Locus_5034.0_Transcript_1 FMRFamide-like 1 (*ppa FMRFamide)

MKFLLLLLCLTFAAANLFELNDCGAAEGSILKRLCAIYQQGEDDDVPNLLRMRQRRDQGYIRFGRSMPISYMRFGRKKREAVDEKRYMRFGRGNEKDDNDAIDVSKKYMRFGKRFQDDLQKKYMRFGKRYMRFGRGGSEDEAEKRYMRFGRNQLKPNEEGIENIDKKYMRFGKRSSSTTN*

**#FMRFamide-like 2**

> DgyrP9978 TRINITY_GG_11498_c0_g1_i1 Locus_3893.0_Transcript_1 FMRFamide-like 2

MKILFLICITCAAINYVHLEELCNIPDGLLKYLCTIHNEEENIAPRVREKREGGYIRFGRSPIDWKLRQKRDINEEKSLPRNQESMNQREQQHIEENKQLDPELESAIEILRLYEVLGSLNKKRLIKFGKRFMRFGKRDDAMYMRFGKDHEGGETKKTYIRFGRNNKEKKQYIRFGRSQ*

**#FLamide-like/FMLamide**

>TRINITY_GG_4470_c0_g2_i1 DgyrP3420 Locus_15189.1_Transcript_1 FLamide-like/FMLamide (*ppa FLamide)

MMTSLFFVLACLLSLGSLQEQENCNRALCGACHLFLSVPQNECCTQPETAQLCGRCLNGAENCDIDKRSSSFWDKRAKFMLGKRPSEFWEKRAKFMLGKRGPAFWDDEEKRAKFMLGKRAKFMLGKRSQFWDKRSDSSAFWEDEKEKKSKRDASAASRMLMDKKQKFMLGKRADV*

**#EFLG-like**

>TRINITY_GG_9906_c2_g1_i7 Locus_17328.3_Transcript_1_6 EFLG-like/ (*ppa EFLG) (on the same transcript as FVamide, similar to *P. dumerilii FV/EFGL*)

MKSNYALFVTLICCCVIVEQFAQEYSPDGYKFDDEENRLYRPILNRFNKRFDEFLGKRFSEFMGKKKRFGDLMGGKKRFSEFMGR*

**#FVamide**

>TRINITY_GG_9906_c2_g1_i1 Locus_17328.3_Transcript_4 FVamide (*ppa FVamide) (on the same transcript as EFLG-like, similar to *P. dumerilii FV/EFGL*)

MKSNYALFVTLICCCVIVEQFAQEYSPDGYKFDDGAQLERQTRKVIYLTPRQLRQLIIRLQSRGKIVRTIGKRSFAVDKRRLFVGKRPDDYAEDWRKEEKRARQRLFVGRRRQLFVGRRSAADDIQ*

**#FVRIamide**

>TRINITY_GG_12815_c0_g1_i1 DgyrP15620 Locus_4594.0_Transcript_1 FVRIamide (*ppa FVRIamide)

MLKFTLSILLVLLASTHTNCETLCSDICEANFPTAEAAACIKECDLKETEILKHLQSEDLNERKRALSSFVRIGRALSSFVRIGRNPEKRLSSFVRIGKSQPVKEMNNFVGIDDGLNEKAKRMSSFVRIGKNVNLDDEKEKRMSSFVRIGKRPMSSFVRIGRSTK*

**#GnRH/Corazonin 1**

>DgyrP11867 TRINITY_GG_15149_c0_g1_i3 Locus_18773.0_Transcript_1 GnRH/AKH/Corazonin 1 (*ppa AKH)

MNRSSVFFIAFACLLLAFQINEIEGQFSFSLPGQWGSGKRSGSNQCLKWDREAFAALQQAMMLQALHYHKCMAAVESKH*

**#GnRH/Corazonin 2**

>TRINITY_GG_15182_c0_g1_i1 Locus_5007.0_Transcript_1 GnRH/AKH/Corazonin 2

MKPIKFAILSILVLLLSNAEGQLTQTLGWGSAGSPGKRNGLSSFCKDSFHKVQFLISAYQKEVWKLKSCRWTNDMK*

**#GnRH/Corazonin 3**

>TRINITY_GG_7207_c0_g1_i1 Locus_6438.0_Transcript_1 GnRH/AKH/Corazonin 3

MNSIQLISVLLLLVTVYQIEQANSQNYHFSNGWQAGKRSTFFTPTKLNDGENSEFPCKVRPSLTKLISKLIEMETRRIMRECYKTSYYNVMSSLKDK*

**#GNxQN-like**

>DgyrP7163 Locus_4061.0_Transcript_1 GNXQN-like (similar to P. dumerilii GNXQN)

MKAAVTLFIFFVFLALNQAAPQAKVRVKRALEQLEYGNHQNRPRVKKADPNVPQKLMPPNPIATKRLEETKSPAPVTKVESDKAAKSDVAIVPKEKTVEEDKELSDIYDWLMKSMKPEMRTNWRKKRSSATAEDMSGAPVQARSKRSLYYDLPMDVYEDEIPFDDDETYPSLYDWKDPYEYYENSEYPTSDSLIPEDEEDISELYGYKSEPTWKRAAPYGLYDTVSEKRSREEAMNRLYALAYRLGKKK*

**#HFAamide**

>Locus_20055.0_Transcript_1 HFAamide

MDRAQLAFMFIGFLLILQPANSFDKASYCVMACQRGTGGNFCQCRASHFAGKRKRNEAEEMEKVKDLRLIKRFLIEEIRRQERQK*

**#HIGA-like**

>Locus_7179.0_Transcript_1 DgyrP47_extended HIGA-like (similar to P. dumerilii HIGA)

MAISVWSISALLLIFQVSDAFEKRHIGAAFGAGFKPSGKRQAPMDLGNWRYADETELPGKRHLGATLNSWISDKRHIGASLNHFSPLLNNKRLYGLRLNGFGKSNGKK*

**#Leucokinin**

>DgyrP6640 CAP3_Contig975 Locus_16399.0_Transcript_1 Leucokinin (*ppa Leucokinin)

MLNYIQLFALLSAVLLCSCLSLPESEEKRAFQSWAGKRAFNAWAGKRAMNSISDQLPQKRQPFQAWAGKRGVGLQSLDALDEERKRAFSSWAGK*

**#Myomodulin 1**

>DgyrP15625 TRINITY_GG_12828_c2_g1_i1 Locus_9170.0_Transcript_1 Myomodulin 1 (*ppa Myomodulin)

MIFAFIKFFLLLVAINSIRSDDGENLMAEKRDLQMLRMGKRYIGDLNDEQKRTLHILRQRSPLPRLGERAPLPRLGERAPLPRLGLLRAAPLPRLGLEDKRVSELDWNEFQEDSRAALPRLGYRAAPLPRLGKEKRDVKLLRMGKRGSKMLQLGKKDHGMLQLGKRQMLRMG*

**#Myomodulin 2**

>DgyrP3885 TRINITY_GG_17083_c0_g1_i1 Locus_19071.0_Transcript_1 Myomodulin 2

MNMKLFICFLLITVSTCEKIAKKDLAMLRMGKRDAVSSFADESSSNFLPPLPRIGKYLEYLDRREEENSNNKRAAMLRMGKRETDFTDDDDEKNIMLRMGRGMSDLRMGKRSMLRMGKRDSPELEKRMMSMLRMGRNVPNKRFSSRDDSDNKRHMSMLRMGKRGAMSMLRMG*

**#MNC-like**

>Locus_19685.0_Transcript_1 MNC-like (similar to P. dumerilii MNC)

MANYKGYFLAFVLLLSVTDHVLSMERESFMDTDISFPKRNPLTIKLLRSLQGARRKRTNCFDLSDMCCLWNVCPNNKR*

**#NKY-like**

>DgyrP13172 Locus_19364.0_Transcript_1_shortened_5prime_end NKY-like

MNRVLIFFALAVACIVVVDSMPRYRAAELFELYPELYEAVYGPALNNVKRRLHGGPLPVATRLAGGFGNKIEGDRNQAPDFKGMRFGRK*

**#NpY/F 1**

>Locus_19137.0_Transcript_1 Neuropeptide Y/F 1 (*ppa NpY1)

MRNVYNILVLLILLGCVDCYRRQFLKNFSRPNSKHTNRVPPPPTRPDKFKSMDEITDYFEQLREYYSVIGRPRYGKRSYTRPSESLLSKPLSISEY*

**#NpY/F 2**

>Locus_3671.0_Transcript_1 Neuropeptide Y/F 2 (*ppa NpY2)

MFRLGSLLILVLIMASVTSDGVPFRSKERRGKTLLHNFNDAKPPVRPAEFKSVADFNDFFMKLREYYSIIGRPRFGRSVKT*

**#NpY/F 3**

>Locus_11302.0_Transcript_1 Neuropeptide Y/F 3 (*ppa NpY3)

MNSTHVYKNNVNFRYINHPQQRNLAEKPIVKMVKTCFIALLAILVVCQVLNAYQTEVDYPVKPEKFDNGEQLKDYLNKMHEYLAIIGRPRWGRDIARVSRILKSQSQN*

**#NpY/F 4**

>Locus_16635.0_Transcript_1 Neuropeptide Y/F 4 (NpY like)

MRMMIMKKNPYLLFVLLFFILLNFKKDTACISLKQPEKFENTQQLYEYLIRLNRYLSVVSRVRWGRDTSRLLRPDAAFHRSIK*

**#NpY/F 5**

>Locus_19301.0_Transcript_1 Neuropeptide Y/F 5 (NpY like)

MLRFGIVFLLFAIGYSAVLAIQQVEQRAPQRPSSFSSPNDIEDFLTKVRAYMKAIGRPRYGG*

**#Pedal peptide 1**

>Locus_11404.0_Transcript_1 Pedal peptide 1 isoform 1 (*ppa Pedal Peptide1)

MRRLALFICLLSVSAASLEKNKNLDELRENYVGSNDIDKKSFDSIGTSAFGGLKKRTFDSIGTSAFGGLKKRTFDSIGTSAFGGLKKRNFDSIGRNSAFSGFKRSFDSIGGSSSFGALRKRPFDSIGSSAFGGLKRSFDAIGSSAFGGMKKKSFDSIGANAFAGFKKRQPIDRTNSPSLWQHRLKYERDITKRSNKDD*

>DgyrP2310 Pedal peptide 1 isoform 2

MRRLALFICLLSVSAASLEKNKKNLDELRENYVGSNDIDKKSFDSIGTSAFGGLKKRTFDSIGTSAFGGLKKRTFDSIGTSAFGGLKKRTFDSIGTSAFGGLKKRNFDSIGRNSAFSGFKRSFDSIGGSSSFGALRKRPFDSIGSSAFGGLKRSFDAIGSSAFGGMKKKSFDSIGANAFAGFKKRQPIDRTNSPSLWQHRLKYERK*

**#Pedal peptide 2**

>DgyrP945 TRINITY_GG_1424_c0_g1_i1 Locus_18751.0_Transcript_1 Pedal peptide 2 (*ppa Pedal Peptide2/MDL)

MKSLAVICSLSVLFVVVLSEDTEKRMIDSIGSSLLKKRMIDPIGSTLLKKRMIDTIGSSLLKKRMIDTIGSSLLKKRMIDTIGSSLLKKRYLDSIGSGLLKRQLDRISDSIFKKDEHERQLDFVPDGHIQFEG*

**#PxFVamide**

>DgyrP9531 TRINITY_GG_8047_c0_g1_i1 Locus_7911.0_Transcript_1 PxFVamide

MRRSVVVFLFLFCFLIAGLSADYYEEFNGKRAPWFVGKRDGRLFIGKKSEGIDERIQRLTELAKEMKRRLRSRPFYIGKRNRMFIDKRDSNDDLTL*

**#QERAS**

>Locus_18907.0_Transcript_1 QERAS (*ppa QERAS)

MATAYISSCSVKGLKYRQRDRVKRKGARVSERERERQSDTMRPTFLILLFVVIVVCQTSAFYLNDKIAKVAQLRKISQESDKMGNEADENTIRPARTKRYTQFQERASSFCTGLCMYHEMRTYSDCFDQCSYLF*

**#RGWamide-like**

>DgyrP11866 TRINITY_GG_15155_c2_g1_i1 Locus_18839.0_Transcript_1 RGWamide-like (*ppa RGWamide)

MKLAVVAFLLIAVTIYVTADESPAEEKRRGWGKRSWGKRSDDEDRFDALEEKRRSWGKRAMGWGKRAMGWGKRGSEEEACERLQQNAMFYTFKAIELENQRQKMCA*

**#RYamide**

> DgyrP9439 TRINITY_GG_7931_c0_g1_i1 Locus_18693.0_Transcript_1 RYamide

MNTLKAVLIACLFLAALTAPISGTEEKQLPGYFDRLRRGIMRYGKRDSSLDEEIFKRIFRYGKRADDGFERQDRNAHIPFRFGEKEE*

**#sCAP**

>Locus_4026.0_Transcript_1 sCAP/small cardioactive peptide like (*ppa sCAP)

MNEKVKIVLLIVSLLQISAALPPEYFRNGRSSGLWPRNLITEICEHCAIYGPNLYNECVQNIGSKRLQCLLIYNADRRDI*

**#SIFamide/FFamide**

>TRINITY_GG_3970_c0_g2_i1 Locus_18810.0_Transcript_1 (*ppa SIFamide)

MKSFVSLVLLGILALVALAHQMEDHPPADSGLFFGKRANPNMNNLLFGRRSSLDPRKVVQATDEFCRNAVTACSNWADKMNEYRH*

**#Sulfakinin-like/GGRFamide**

>DgyrP5008 Locus_13823.0_Transcript_2 Sulfakinin-like/GGRFamide

MLSSKLITVLGAILVCSLVFSAATETANENTDLEQCKIFFNKALEIIRSGRDKRQKTYADYGWGGGRFGKKRSSDADFKKRIAHDLLQVGGRFGRDVKNV*

**#Tachykinin**

>Locus_4282.0_Transcript_1 Tachykinin

MQFSASIFLLLVVPFVYLEAYRLTGYNKELYDATEKNVERLSSLENLKREISKVQDVLFKRIYAAEQLGISSDGEEKRSGFVPSRGRRNWSRDRRSPSPFTAMRGKRKFYGTRGKKSELDYLSKFSDTELKRNSFAAMRG*

**#Vasotocin**

>TRINITY_GG_14248_c0_g1_i1 Locus_20304.0_Transcript_1 Vasotocin (*ppa Vasotocin)

MNKLRAKALFLVLLQASITSTCFIRDCPPGGKRNFNIPQENNQCSRCGPRDEGQCIARNICCGFSIGCLVHNDYEEVCSTTNENCEIKGETCEAVNGGKCVTDGICCKHGRCMFDIKCKERNLKMARNNKNLILNHLTRALLPEKELESNLG*

**#Whitnin**

>DgyrP7735 CAP3_Contig1261 Locus_18565.0_Transcript_1 Whitnin (*ppa Whitnin)

MYVKALLLLVFVVAVSSMSLQTADEGQQFKRTQTEDDESRATWLDTREDLLSNFKNFVYSSVVELVNENKLDGSVLSAPSKDKRGRWQGFCFRRNKQGKFLPYICWKGGK*

**#WI**

>DgyrP1680 Locus_4054.0_Transcript_1 WI peptide isoform 1 (*ppa WI)

MEVYFESVKRRKSPTPKRRPSVPMTYFRLLLLLLIQTGVEASDCKVSERIDLTKKDLPECSGKKEIGGLIRNEDCFPIKNSVMDIFTDTCRVTVITTDQGMFKAKVDGRVRAISVRLGDNGEEFYSIPMGNEKIDIVVETTPSSSNGRVKRSLDRRMWQGAGFEWLKRNPWRYSNSNWLKKRNWADINSEWLKKRSWDEAGMDWLRRRRRSADKKAWSEAGIGWIKRSDDSDKRAWNDAGFGWVKKNAADKKAWEDAGIGWVKKNSPDKKAWEDAGIGWVKKDLNKKAWSDAGFGWVKKNASDKKAWHDAGIGWIKRNSDTENKRAWSDAGINWVKRQNEHKDAGEQRRD*

>TRINITY_GG_9060_c1_g2 WI peptide isoform 2

MNMEVYFESVKRRKSPTPKRRPSVPMTYFRLLLLLLIQTGVEASDCKVSERIDLTKKDLPECSGKKEIGGLIRNEDCFPIKNSVMDIFTDTCRVTVITTDQGMFKAKVDGRVRAISVRLGDNGEEFYSIPMGNEKIDIVVETTPSSSNGRVKRSLDRRMWQGAGFEWLKRNPWRYSNSNWLKKRNWADINSEWLKKRSWDEAGMDWLRRRRRSADKKAWSEAGIGWIKRSDDSDKRAWNDAGFGWVKKNAADKKAWEDAGIGWVKKKFSR*

> CAP3 = genome guided transcriptome

> TRINITY = genome guided transcriptome

> Dgyr = gene models transcriptome

> Locus = old transcriptome

*ppa = “previously published as” (in: Kerbl, A. Conzelmann, M., Jékely, G., Worsaae, K. (2017): High diversity in neuropeptide immunoreactivity patterns among three closely related species of Dinophilidae (Annelida). Journal of Comparative Neurology, 525: 3596-3635.)

**Color code:**

Signal peptide

(di-)basic cleavage site

Amidation site

Cysteine residue
