# Supplementary material for: Conservative route to genome compaction in a miniature annelid
Source: Nat Ecol Evol. 2020 Nov 16;5(2):231–42. doi: 10.1038/s41559-020-01327-6 (PMC7854359; doi:10.1038/s41559-020-01327-6)
Supplement: Supplementary file 3 — Peer Review Information [file 41559_2020_1327_MOESM3_ESM.pdf]

natureresearch

Peer Review Information

Journal: Nature Ecology & Evolution
Manuscript Title: Conservative route to extreme genome compaction in a miniature annelid
Corresponding author name(s): José M. Martín-Durán, Andreas Hejnol

Editorial Notes:

Reviewer Comments & Decisions:

Decision Letter, initial version:

2nd June 2020

*Please ensure you delete the link to your author homepage in this e-mail if you wish to forward it to your co-authors.

Dear Andreas,

Your manuscript entitled "Conservative route to extreme genome compaction in a miniature annelid" has now been seen by three reviewers, whose comments are attached. The reviewers have raised a number of concerns which will need to be addressed before we can offer publication in Nature Ecology & Evolution. We will therefore need to see your responses to the criticisms raised and to some editorial concerns, along with a revised manuscript, before we can reach a final decision regarding publication.

We therefore invite you to revise your manuscript taking into account all reviewer and editor comments. Please highlight all changes in the manuscript text file in Microsoft Word format.

We are committed to providing a fair and constructive peer-review process. Do not hesitate to contact us if there are specific requests from the reviewers that you believe are technically impossible or unlikely to yield a meaningful outcome.

When revising your manuscript:

* Include a “Response to reviewers” document detailing, point-by-point, how you addressed each reviewer comment. If no action was taken to address a point, you must provide a compelling argument. This response will be sent back to the reviewers along with the revised manuscript.

* If you have not done so already please begin to revise your manuscript so that it conforms to our Article format instructions at http://www.nature.com/natecolevol/info/final-submission. Refer also to any guidelines provided in this letter.

* Include a revised version of any required reporting checklist. It will be available to referees (and, potentially, statisticians) to aid in their evaluation if the manuscript goes back for peer review. A revised checklist is essential for re-review of the paper.

Please use the link below to submit your revised manuscript and related files:

[REDACTED]

Note: This URL links to your confidential home page and associated information about manuscripts you may have submitted, or that you are reviewing for us. If you wish to forward this email to co-authors, please delete the link to your homepage.

We hope to receive your revised manuscript within four to eight weeks. If you cannot send it within this time, please let us know. We will be happy to consider your revision so long as nothing similar has been accepted for publication at Nature Ecology & Evolution or published elsewhere.

Nature Ecology & Evolution is committed to improving transparency in authorship. As part of our efforts in this direction, we are now requesting that all authors identified as 'corresponding author' on published papers create and link their Open Researcher and Contributor Identifier (ORCID) with their account on the Manuscript Tracking System (MTS), prior to acceptance. ORCID helps the scientific community achieve unambiguous attribution of all scholarly contributions. You can create and link your ORCID from the home page of the MTS by clicking on 'Modify my Springer Nature account'. For more information please visit please visit www.springernature.com/orcid.

Please do not hesitate to contact me if you have any questions or would like to discuss these revisions further.

We look forward to seeing the revised manuscript and thank you for the opportunity to review your work.

[REDACTED]

Reviewer expertise:

Reviewer #1: invertebrate genome sequencing and evolution

Reviewer #2: invertebrate genome sequencing and evolution

Reviewer #3: invertebrate genome sequencing and evolution

Reviewers' comments:

Reviewer #1 (Remarks to the Author):

Martín-Durán

Martín-Durán and colleagues present the genome of the annelid Dimorphilus and analyse its genome structure and content in the context of other annelids and other Metazoa.
One claim, emphasised in the abstract is that the genome is small and unusually so. Comparisons and claims are made to parasites, and a contrast made concerning the non-parasitic lifestyle of Dimorphilus. However it is clear that other freeliving metazoan species also have small genomes, and that genome compaction (like genome expansion) can occur through a number of processes.

"With a ~68 Mb size, Dimorphilus genome is the second smallest ever decoded for a free-living animal."
I am not sure why this claim is made. Many free-living nematodes have genomes of the order of 60 Mb: which other species is being compared to? C. sulstoni? O. dioica?
I also note that the size of the genome is given as 78 Mb by assembly in the results section and in Supp Table 5, so I am unclear of the origin of this 68 Mb claim. In figure 1 the genome size is given as 0.072 Gb, yet another value.
In passing the reassembly and reannotation of R. varieornatus by Yoshida (doi.org/10.1371/journal.pbio.2002266) is the current reference version.

"genome expansions might also increase the evolvability of a lineage by providing new genetic material that can stimulate species radiation and the evolution of new genome regulatory contexts and gene architectures"
The authors acknowledge the neutral models for genome size change as largely driven by Ne but (as do most biologists) seek adaptive explanations for the apparent success of some changes: I do not think there is evidence for increased "evolvability" of lineages through expansion compared to lineages that do not show expansion. Reference 27 (and Lynch's book) are key. In passing, the connection between parasitism and genome size in animals is unclear - in nematodes, the parasitic Meloidogyne (the Abad M. hapla cited) range from ~55 to ~275 Mb, freeliving C. elegans is 100 Mb, parasitic Ascaris is 275 Mb and parasitic Heligmosomoides is 700 Mb. Of sequenced genomes, the parasites are actually larger... but this is likely sampling bias. For analysis of genome size correlations with genome architecture elements in freeliving taxa see https://doi.org/10.1002/evl3.110.

"a highly contiguous (N50, 2.24Mb) and complete (95.8% BUSCO genes) ~78Mb-long haploid assembly", "Flow cytometry measurements and k-mer based analyses estimated Dimorphilus genome size in ~72Mb and ~65Mb genome, respectively"
The data in Figure 1 elicit concern, particularly panel D.
(a) Panel c: The C. elegans genome is 103 Mb (see wormbase for the latest information re genome size from long read assemblies generated in the last 4 or so years that correct the earlier 100.2 Mb estimates)
(b) Panel d: The trace for T axi looks as would be expected for a diploid with very very little heterozygosity - is this an inbred individual? The trace for Dimorphilus is very odd indeed. They take an estimate of 65 Mb from this but I am quite sceptical that these data can support such a claim. The pattern does not have the expected haploid and diploid peaks, a pattern that should be very evident at the coverage claimed (139x).

"To calculate the depth, we identified the peak coverage of unique 31-mers (139x; Figure 1d), which corresponds to the homozygous peak, and estimated the haploid genome size of D. gyrociliatus in 64,794,005 bases or 64.8 Mbp following the formula described elsewhere89."
The assembly methodology involved extensive cleaning of the data of contaminants, but we are not told what these were. The final assembly was used to select Illumna reads. In the methods these were then corrected before being assessed for kmer content. This is all quite obscure to me. A tool like KAT or Genomescope should be used for kmer analysis (the 2003 Li paper has been superseded), and this would also identify / estimate heterozygosity, uncollapsed haploid segments and genomesize. The smudgeplot toolkit also permits estimates of effective ploidy.
The authors provide a heterozygosity estimate of 0.001. Given this (one base difference in 1000, ie near homozygosity in general), one would expect a clear single homozygous diploid peak in their k=31 frequency plot in (d). Instead we have a very poorly differentiated, possibly double peaked distribution, with the lower frequency peak claimed to be "homozygous". These data do not add up: either the animals were heterozygous, and the two peaks are haploid (lower freq) and diploid (higher), or the animals are homozygous (from SNP calling) in which case the distribution of k-mer frequencies needs some detailed explanation.

"The most abundant TE class is a Ty3-gypsy-like LTR retrotransposon, whose envelope (env) protein shows similarities with that of animal Herpesviridae"
I presume this is the "Dingle-1" element referred to later? I can find no definition of this element in the text or supp text, and would ask the authors to make the origin of the nomenclature clear.
The match to a herpesvirus is very odd, as Herpesviridae are DNA viruses, and their envelope proteins are multiple (32 or so are involved in envelope function) and very different... Which protein is matched (no data are given)?
The supp figure panel c has a taxon "Annelidae" a likely typo.

"Therefore, gene and intron loss, rather than short intron size – which was likely a pre-existing condition – correlates with genome compaction in Dimorphilus. ... Therefore, our data suggests that gene repertoire streamlining outweighs complete gene family loss"
I refer the authors to the work of Stevens on similar analyses across Caenorhabditis (https://doi.org/10.1002/evl3.110 https://doi.org/10.1186/s40850-017-0013-2).

Extended data Figure 4 panel g illustrates the lack of conserved kmers in the 5' UTRs of the transcripts, not genome-derived genes but this is not made clear (other panels are derived from the genome annotation).

In Fig 2 the meaning of several sets of numbers is not explained, especially evident (after much magnification) in panel d. The legends need to describe these numbers explicitly.

The synteny analysis is fascinating and nice to see. Do the authors know the karyotype of Dimorphilus?

The analysis of conserved genetic repertoire in the face of gene loss and genome compaction is indeed striking, and the Hox cluster analyses are particularly interesting. Can the authors rule out rapid temporal collinearity in a rapidly developing embryo (ie the possibility that the temporal cadence has been compressed to span a short period not sampled by their analysis)?

"our data suggest a link between gene loss, genome compaction and morphological miniaturisation in

Dimorphilus, previously unrecognised in any other free-living animal with a miniature genome"
were these not also the conclusions from the Oikopleura analyses?

General comment
The figures as constituted I presume are expected to be on an "A4" page. If this is the case, some of the text will be impossible to read without a magnifying glass (and comparing data on mapped figures near impossible). The authors should decide which panels are essential and move the others to Supplemental information, perhaps.

Reviewer #2 (Remarks to the Author):

Comments to Authors
In this manuscript, Martín-Durán et al. sequenced, assembled, and analyzed the genome of Dimorphilus gyrociliatus, a meiobenthic, miniaturised annelid that lays claim to having the second-smallest genome encoded yet for a free-living animal. The effort expended in this manuscript represents a tour-de-force that examines this genome using state of the art sequencing technologies (e.g. PacBio long reads, ATAC-seq, CAGE-seq) in addition to applying a large suite of standard analyses to the genome assembly. The Dimorphilus genome shows high levels of conservation in terms of gene content and synteny, including a well-preserved and ordered gene Hox cluster. This is striking, and deviates from what we have come to expect from highly compacted genomes. ATAC-seq, and CAGE-seq provided a view of the regulatory landscape of the Dimorphilus genome revealing elements similar and divergent relative to other bilaterian genomes.

The striking conservation of gene content and synteny despite reduced genome size is an important finding that will be of interest to a wide audience. This genome also adds to a paucity of genome data from a highly speciose but understudied phylum. The many datasets generated for this study will also be a tremendous resource for other researchers in the fields of phylogenomics and evolutionary developmental biology. The analyses and writing are of high quality, and are extensive. Most of my comments relate to some premature conclusions the authors make based on the data presented. These conclusions are highly speculative and the authors should be deliberate in making it clear that these ideas are hypotheses that need to be tested (much of which is not possible in the near term). Further, there were instances of very dense figures with several analyses that ended up being summarized in one sentence. These would benefit from a little more elaboration.

Major Comments

Hox cluster analysis and expression
The claim in Figure 3a legend that “Whole mount in situ hybridisation during embryogenesis reveals that Hox genes exhibit spatial, but not temporal collinear expression domains” or line 196 “Hox genes show only spatial collinearity” is not clear from the figure. The panel is extremely small, and not enough information is provided to the reader about this poorly-studied species’ embryos to be able to interpret the data. To assess if the domains are collinear, one would need double in situ hybridizations or need very clear landmarks observable in the embryo that show that the gene order corresponds to the localization of expression patterns along the AP axis. The statement in the text “Hox genes are expressed in staggered anteroposterior domains along the developing trunk,” is more conservative, and is well-supported by the figure as presented.

Growth/Proliferation pathway analysis
Why were the kynurenine and Myc pathways chosen as a way to assess growth/proliferation pathways? Was there a systematic/unbiased analysis that showed that proliferation/growth-associated pathways were more likely to show divergence or gene loss relative to other pathways? To assess how meaningful the loss of genes or changes in amino acid residues in these pathways is, it is important to know whether Dimorphilus has done away with all pathways that regulate these processes. For example, was the Hippo pathway considered? What about Akt, mTOR? Without a full understanding of other growth/proliferation pathways, the statement in the Discussion “Dimorphilus exhibits losses and genetic modifications of metabolic (kynurenine pathway) and cellular (Myc) pathways involved in growth, proliferation, and senescence, and that when deregulated cause genomic instability, but also hypoplasia, dwarfism, and premature aging.” currently reads as though these pathways have been cherry-picked for analysis.

Further, the authors should temper their statement “these genomic changes indicate the first known mechanistic link between genome size reduction and organism miniaturisation, suggesting Myc and sirtuins as potential candidates connecting genome size with metabolism30, developmental rate31, and body/cell size1,32.” Since the identification of a mechanistic link would require functional studies, this is better written as “these pathways provide a substrate for studying whether there is a mechanistic link between genome size reduction and organism miniaturisation…”

CTCF and regulatory biology
The finding that CTCF binding sites are highly enriched in promoter regions is interesting. However, is an ortholog of CTCF encoded in the genome of Dimorphilus? I could not find this information easily in the manuscript. Further, the reduced intergenic space in this genome, together with the enrichment of CTCF sites in promoters actually suggests that the role of CTCF in regulating the genome is likely quite different from its role in vertebrates, where it tends to be present far from transcriptional start sites and marks TAD boundaries. Therefore, the authors should re-write statements such as this: “The CTCF DNA-binding motif was the most abundant in active regulatory regions, located mostly in promoters and as single motifs (Fig. 4e; Extended Data Fig. 9e–h), supporting that, unlike nematodes with compact genomes63, CTCF plays a major regulatory role in Dimorphilus genome, as observed in vertebrates64”. The data presented show that in contrast to nematodes, which don’t have a CTCF ortholog, the Dimorphilus CTCF (if present) could be playing a role in regulating gene expression. But this role is likely quite different from its function in vertebrates. The sentence, as written, can be interpreted as suggesting that regulatory roles between vertebrates and Dimorphilus are shared.

Absence of long-range regulation and GRNs
In lines 278-282, the authors suggest that the absence of long-distance gene regulation would imply simplification or dismantling of gene regulatory networks. I don’t understand the logic behind this. GRNs describe regulatory relationships of transcription factors and their target genes, which could be mediated via proximal regulatory elements. The promoter regions of Dimorphilus genes could be chock full of binding sites and the animal could have many GRNs, be they conserved or lineage-specific. If the genome compaction was so good at retaining gene families by trimming our paralogs, it could also have been great at keeping essential regulatory relationships intact, just bringing the regulatory information closer together. Since the authors don’t have data that can assess GRNs, they should reconsider this inference/suggestion.

Extended data figure 8 (life cycle transcriptome sequencing)
This figure represents analysis of a large amount of RNA sequencing data from different life history

stages. Extensive analysis is described in the supplementary info (section 3.3). However, the only reference to this figure in the main text was in the context of Hox gene expression (line 189: Stage-specific transcriptomics (Extended Data Fig. 8) and gene expression analyses during Dimorphilus life cycle indicate that all Hox genes butHox5 and Antp become simultaneously transcribed in early development, after gastrulation). The Figure was too low resolution to be able to see what the volcano plots were showing, and I did not find a supplemental file (e.g., spreadsheet of differentially expressed genes) associated with this. The authors should assess whether releasing these data as part of this manuscript is necessary, or they should explain the figures shown. Without that, this impressive-seeming work fails to be meaningful for the community.

Minor Comments
- line 246: “our data indicates that Dimorphilus has retained a conserved genomic regulatory profile”. The authors should clarify in this statement what they mean by “genomic regulatory profile”, particularly because the subsequent phrases point out how the Dimorphilus genome deviates from other bilaterian genomes.

- It would be helpful to make the index at the beginning of the supplementary info file more detailed. e.g, listing what is contained in the supplementary tables, and a bit more detail for the data files.

Reviewer #3 (Remarks to the Author):

This is a solid paper reporting the genome of a miniature annelid, Dimorphilus gyrociliatus. The animal is interesting, the genome quality is good, the analysis is, for the most part, sound and the paper is reasonably clear. Of particular note, the work goes beyond a gene inventory and includes study of regulatory structure via ATAC-seq analysis.

My reservations are centred around some points that the I think the authors' have stretched too far.

Major points:

1. As a general issue, the authors need to take more care not to conflate the 'miniature annelid' and the 'small genome' stuff, particularly when talking about mechanism, unless they are explicitly meaning to link the two things. I am sure they are clear in their minds about this, but as an uninitiated reader I kept having to stop to think about it. An explicit evidence based statement in the introduction about whether the authors think that a link between genome size and body size is tenable might help clarify later sections (they appear to be chasing this hypothesis). Lines 212 to 231 are poor in this regard. In essence what are the hypotheses? Do they think there is a direct connection, with genome size causing body size? Are these things manifestations of a single phenomenon, or two different ones?

2. One of the abstract headlines, the kynurenine pathway has some issues. How was this pathway identified? Given that this is a global analysis, was this the only such pathway identified (notwithstanding the other genes mentioned)? What were the criteria for choosing? In essence, can we be confident that this is one of the only pathways affected?

3. The kynurenine pathway is problematic in that several of these genes appear to be absent in Drosophila (searching with human KYNU, HAAO, QPRT). Drosophila is fairly small, and has a fairly small genome - do the authors think this is a data point that is consistent with their broader hypothesis? Why don't they mention it? What about other animals (small and large genomes and bodies)? What about the status in other animals of the other genes and pathways that they mention?

Other points:

L.51 - 'unlike animals with small genomes' - probably should be 'unlike other animals...
'
L.140 - not clear what the authors mean by 'very short introns' when in the same sentence these introns are not shorter "than in Capitella and even the centipede".

L.141 - I don't see how a metric that measures % intron retention shows that one species or another had 'adapted' its splicing to deal with short introns. The authors seem to be arguing that the human splicing machinery is not adapted to removing human introns, or that Dimorphilus isn't adapted to removing its own long introns.

L.151 "we first reconstructed gene families". It really isn't clear what this means. Were other species included? "80% of Dimorphilus genes are assigned to shared gene families" - what does 'shared' mean? The main text should at least have a basic outline of this.

L.157 "Capitella which is the most conservative sequenced annelid genome" ref 48 can't support that statement as most annelids have been sequenced since that was published. I'm not aware of any published work on this.

L.160 'gene family streamlining' - as an observation, genes don't know what family they're in, so in itself this doesn't make sense from a mechanistic point of view.

L.205, Macrosynteny - legend to Figure 3b/S7f should explain what is being shown and why it supports the argument of the main text. For example, what does the color scheme represent? The resolution of these figures should be improved.

L.293 'the first known mechanistic link between genome size reduction and organism miniaturisation'. I don't understand what the authors are claiming here. 'known' implies some sort of consensus or acceptance. Wouldn't 'a possible mechanistic link' be more appropriate? They are far away from being able to state that one single mechanism causes both genome size reduction and body size reduction. This needs to be rewritten making clearer what the results are and what is the authors' interpretation.

L.683 "1980'ies" -> "1980s"

********************END********************

Author Rebuttal to Initial comments

Response to Reviewers

We would like to thank the reviewers for their positive appraisal of our manuscript, and for providing a range of comments and suggestions that we believe have significantly improved our work. Following reviewer #1 suggestions, we revised our k-mer analyses and used KAT and GenomeScope2.0 tools to calculate the size of *Dimorphilus* genome, obtaining a genome size of 70.95 Mb, consistent with previous estimations and our assembly size. Given these new analyses and the size obtained from flow cytometry, we now report a genome size of 73.82 Mb as a reasonable and conservative estimation, which we consistently use throughout the manuscript. Following reviewers #2 and #3 comments, we now provide a more thorough characterisation of growth/proliferation signalling pathways employing genome wide annotation data, demonstrating that major mechanisms to control cell and body size/growth are intact in *Dimorphilus*, except the MYC transduction pathway. Moreover, the analysis of the presence/absence of the kynurenine pathway in Metazoa (reviewer #3 suggestion) has shown that this pathway has diverged in lineages with both large and small genomes, and large and small body sizes, thus suggesting that divergences in this metabolic pathway might not be related to miniaturisation and genome compaction. Therefore, we have removed these findings from the manuscript. Finally, we have incorporated all text changes and suggestions, and distributed all main findings into six display items to increase the size of the panels and fonts in figures.

Below we provide a detailed point-by-point response to each of the reviewers' concerns.

Reviewer #1 (Remarks to the Author):

Martín-Durán and colleagues present the genome of the annelid Dimorphilus and analyse its genome structure and content in the context of other annelids and other Metazoa.

One claim emphasised in the abstract is that the genome is small and unusually so. Comparisons and claims are made to parasites, and a contrast made concerning the non-parasitic lifestyle of Dimorphilus. However, it is clear that other freeliving metazoan species also have small genomes, and that genome compaction (like genome expansion) can occur through a number of processes.

ACTION: We have removed the superlative "extreme" from the expression "extreme genome compaction" and "extreme compact genome" in the title, abstract and throughout the text. In addition, we have modified the first sentences of the abstract to remove direct comparisons with parasites and other free-living animals and also reduce the length of this introductory paragraph to 150 words, following editorial guidelines (lines 41-43).

"With a ~68 Mb size, Dimorphilus genome is the second smallest ever decoded for a free-living animal." I am not sure why this claim is made. Many free-living nematodes have genomes of the order of 60 Mb: which other species is being compared to? C. sulstoni? O. dioica?

ACTION: We used Genome Size database (http://www.genomesize.com) as reference to make that claim. However, and as pointed out by the reviewer in a comment below, there are recent studies that have uncovered free living species of the genus *Caenorhabditis* (e.g. *C. sulstoni* and *C. bovis*) with genomes of similar size to *Dimorphilus*, which are not included in Genome Size database. We have thus deleted this statement from the introductory paragraph.

I also note that the size of the genome is given as 78 Mb by assembly in the results section and in Supp Table 5, so I am unclear of the origin of this 68 Mb claim. In figure 1 the genome size is given as 0.072 Gb, yet another value.

ACTION: We now provide a consistent value of 73.82 Mb (or 74 Mb when rounding up), based on flow cytometry. The revised k-mer based genome size estimations (see points below) give a size of 70.95 Mb, which are consistent with previous size calculations and our assembly. However, the flow cytometry estimation fits well with the size of the assembly when only gene-containing scaffolds are considered (~73 Mb). To be more conservative, we thus decided to adopt that estimation throughout the text.

In passing the reassembly and reannotation of R. varieornatus by Yoshida (doi.org/10.1371/journal.pbio.2002266) is the current reference version.

ACTION: We included the suggested reference when referring to the small size of *R. varieornatus* (e.g. lines 75 and 78).

"genome expansions might also increase the evolvability of a lineage by providing new genetic material that can stimulate species radiation and the evolution of new genome regulatory contexts and gene architectures" The authors acknowledge the neutral models for genome size change as largely driven by Ne but (as do most biologists) seek adaptive explanations for the apparent success of some changes: I do not think there is evidence for increased "evolvability" of lineages through expansion compared to lineages that do not show expansion. Reference 27 (and Lynch's book) are key.

ACTION: We thank the reviewer for highlighting the importance of Michael Lynch's work, and we now include a reference to this book. We have also removed the expression "increase the evolvability of a lineage" (lines 62-65). We have also removed the mention to the "adaptive value" in the following sentence (line 66).

In passing, the connection between parasitism and genome size in animals is unclear - in nematodes, the parasitic Meloidogyne (the Abad M. hapla cited) range from ~55 to ~275 Mb, freeliving C. elegans is 100 Mb, parasitic Ascaris is 275 Mb and parasitic Heligmosomoides is 700 Mb. Of sequenced genomes, the parasites are actually larger... but this is likely sampling bias. For analysis of genome size correlations with genome architecture elements in freeliving taxa see https://doi.org/10.1002/evl3.110.

ACTION: We thank the reviewer for pointing this issue out and suggesting new references. We now indicate that the connection between genome size and parasitism is variable (lines 70-73)

"a highly contiguous (N50, 2.24Mb) and complete (95.8% BUSCO genes) ~78Mb-long haploid assembly", "Flow cytometry measurements and k-mer based analyses estimated Dimorphilus genome size in ~72Mb and ~65Mb genome, respectively"

The data in Figure 1 elicit concern, particularly panel D.

(a) Panel c: The C. elegans genome is 103 Mb (see wormbase for the latest information re genome size from long read assemblies generated in the last 4 or so years that correct the earlier 100.2 Mb estimates)

ACTION: We have updated *D. gyrociliatus* genome size estimation employing the most updated size for C. elegans. *D. gyrociliatus* genome size is now at 73.82 Mb and we have amended the main text (line 104) and Figure 1c accordingly.

(b) Panel d: The trace for T axi looks as would be expected for a diploid with very very little heterozygosity - is this an inbred individual?

ACTION: *Trilobodrilus* adults were collected from the wild and kept in the Worsaae's lab. We now clarify this issue in Supplementary Materials (line 123-124). We have re-calculated *Trilobodrilus* genome size employing the pipelines the reviewer suggests below and obtained a haploid length of 92.47 Mb and heterozygosity estimation of 0.42% (Supp. Materials, section 2.2), almost three times higher than for *D. gyrociliatus*, which is thus consistent with an input sample of pooled wild specimens.

The trace for Dimorphilus is very odd indeed. They take an estimate of 65 Mb from this but I am quite sceptical that these data can support such a claim. The pattern does not have the expected haploid and diploid peaks, a pattern that should be very evident at the coverage claimed (139x).

RESPONSE: We agree the k-mer profile for *Dimorphilus* is unusual, and thus prevents an accurate genome size estimation. To investigate the origin of this odd pattern, we re-analysed and processed the corresponding Illumina dataset from scratch and found no issues with the overall quality of the data. The raw reads pass all FastQC tests and the only warnings – over-represented sequences and duplication levels – are gone after adapter trimming and read deduplication without impacting the total number of sequences (1.8% and 5.5% of reads removed, respectively). The odd k-mer spectrum was also unaffected by the removal of contaminant reads matching algae and bacteria (3.9%). The remaining 126M read pairs have a high mapping rate and alignment quality to the assembly, suggesting these k-mers correspond to real genomic data. We finally identified the issue using the suggested tool KAT to plot the k-mer coverage by GC counts. The plot reveals a linear positive correlation between the GC content and the coverage of genomic k-mers (Figure 1a below), suggesting there is a GC content bias in the sequencing data. This bias often leads to the uneven coverage between AT-rich and GC-rich regions of the genome (see Benjamini and Speed 2012, DOI: 10.1093/nar/gks001). We found that to be the case for this *Dimorphilus* Illumina dataset. AT-rich regions can have 50% less coverage than the average read coverage across the genome, while GC-rich regions can have 50% more coverage than the average, as reported by the tool CollectGcBiasMetrics from Picard. This uneven read coverage skews the k-mer spectrum and is the origin of the odd pattern in the initial *Dimorphilus* trace. Fortunately, we can mitigate the bias by normalising the read coverage to a value lower than 100x, which comprises most of the genomic k-mers. After normalisation, we obtain a clean k-mer spectrum that allows a more accurate genome size and heterozygosity estimations (Figure 1b below).

ACTION: We have updated the k-mer plot in the manuscript (Fig 1d) now using the coverage-normalised data to estimate the genome size of *Dimorphilus*. We have incorporated the suggested k-mer analysis tools and have updated the estimates accordingly. We obtain a haploid genome length of 70.95 Mb using GenomeScope2.0, 70.35 Mb using KAT and 73.10 Mb using the canonical formula of k-mer abundance divided by peak coverage. We have updated the manuscript to reflect these changes using the estimates from GenomeScope2.0, which we find to be more robust, in particular for the heterozygosity estimates (Supplementary Figure 2).

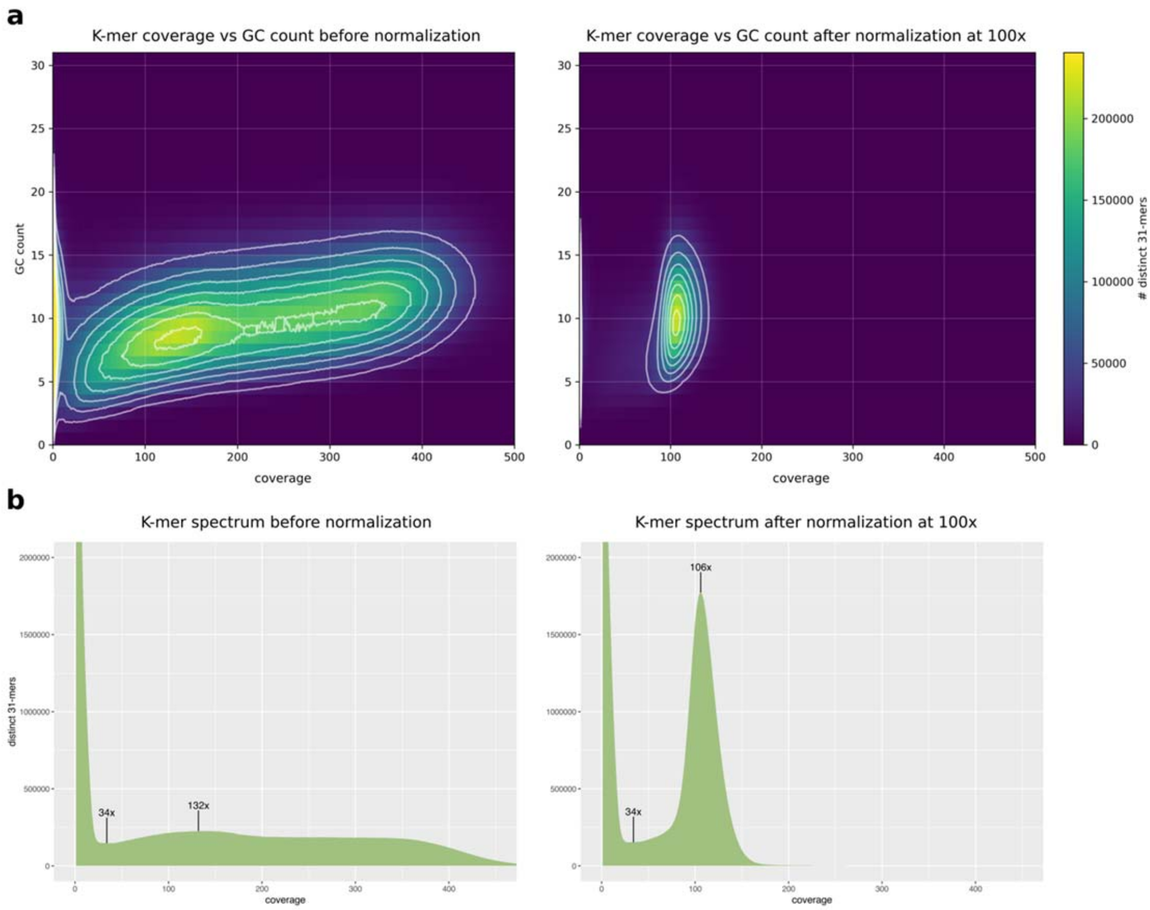

Figure 1: Effect of coverage normalization in the k-mer spectrum of *D. gyrociliatus* Illumina dataset. (a) GC content bias evidenced by a positive correlation between k-mer coverage and GC count (left) is mitigated by normalizing the read coverage (right). (b) The odd pattern from the original data (left) resolves into a clear homozygous peak after read coverage normalization (right).

"To calculate the depth, we identified the peak coverage of unique 31-mers (139x; Figure 1d), which corresponds to the homozygous peak, and estimated the haploid genome size of D. gyrociliatus in 64,794,005 bases or 64.8 Mbp following the formula described elsewhere89." The assembly methodology involved extensive cleaning of the data of contaminants, but we are not told what these were.

RESPONSE: The main contaminants we found in the Illumina and PacBio reads were Proteobacteria and Streptophyta, and to a lower extent other bacteria, fungi, and unicellular eukaryotes.

ACTION: We included a sentence to describe the contaminants removed from the assembly in Supplementary Materials (line 112-113).

The final assembly was used to select Illumina reads. In the methods these were then corrected before being assessed for kmer content. This is all quite obscure to me. A tool like KAT or Genomescope should be used for kmer analysis (the 2003 Li paper has been superseded), and this would also identify / estimate heterozygosity, uncollapsed haploid segments and genomesize. The smudgeplot toolkit also permits estimates of effective ploidy.

ACTION: We thank the reviewer for pointing this out and for the suggested tools. We have revised the paragraph in the Supplementary Material and now describe in greater detail the pre-processing of the Illumina reads for the k-mer analysis, including the coverage normalisation as described above (section 2.2, line 170). As mentioned above, we now use GenomeScope2.0 to estimate the genome sizes and heterozygosity and generate Spectra-CN plots using KAT to visualize the copy number spectra between the sequencing reads and assemblies (Supp Figure 1). For both species, the reported completeness based on the proportion of shared k-mers between the sequencing reads and the assembly is high, and the fraction of un-collapsed haploid segments is low. We now report this finding in the Supplementary Material (line 151-156). Finally, we used Smudgeplot to verify the effective ploidy for *D. gyrociliatus* and *T. axi* and both show a diploid pattern without evidence for higher ploidy levels (Supplementary Figure 3).

The authors provide a heterozygosity estimate of 0.001. Given this (one base difference in 1000, ie near homozygosity in general), one would expect a clear single homozygous diploid peak in their k=31 frequency plot in (d). Instead we have a very poorly differentiated, possibly double peaked distribution, with the lower frequency peak claimed to be "homozygous". These data do not add up: either the animals were heterozygous, and the two peaks are haploid (lower freq) and diploid (higher), or the animals are homozygous (from SNP calling) in which case the distribution of k-mer frequencies needs some detailed explanation.

RESPONSE: We thank the reviewer for rightly spotting this incongruency in our results. As described above, we found the poorly differentiated k-mer spectrum of *D. gyrociliatus* was an artefact due to uneven coverage of the Illumina data. Our revised coverage-normalized k-mer analysis reveals a clear single homozygous peak and 0.17% heterozygosity as estimated by GenomeScope2.0. We also find a similar pattern for *T. axi* with a single homozygous peak, but higher heterozygosity (0.42%). The difference is in accordance with *Dimorphilus* being sampled from an inbred laboratory culture while *Trilobodrilus* from animals collected in the wild. We have removed the SNP calling analysis from the manuscript because it might be affected by the GC content bias in *D. gyrociliatus* Illumina data.

"The most abundant TE class is a Ty3-gypsy-like LTR retrotransposon, whose envelope (env) protein shows similarities with that of animal Herpesviridae" I presume this is the "Dingle-1" element referred to later? I can find no definition of this element in the text or supp text, and would ask the authors to make the origin of the nomenclature clear.

ACTION: The name comes from Dinophilidae *Gypsy*-like elements (Dingle). We now clarify this point in the main text (line 123).

The match to a herpesvirus is very odd, as Herpesviridae are DNA viruses, and their envelope proteins are multiple (32 or so are involved in envelope function) and very different... Which protein is matched (no data are given)?

RESPONSE: We thank the reviewer for pointing this issue out for further clarification. Similarity of ENV proteins of Gypsy retrotransposons to DNA viruses is not unique to Dingle, since ENV proteins of Gypsy elements of lepidopterans and dipterans show similarities to the dsDNA virus family Baculoviridae.

ACTION: We now clarify that the best match from structural prediction is against CMV and HSV-1 envelope glycoprotein B precursor and that similarity of ENV protein with DNA viruses is shared with insect and nematode lineages (lines 123-127). This information is also included in Extended Data Figure 3e.

The supp figure panel c has a taxon "Annelidae" a likely typo.

RESPONSE: Typo corrected.

"Therefore, gene and intron loss, rather than short intron size – which was likely a pre-existing condition – correlates with genome compaction in Dimorphilus. ... Therefore, our data suggests that gene repertoire streamlining outweighs complete gene family loss" I refer the authors to the work of Stevens on similar analyses across Caenorhabditis (https://doi.org/10.1002/evl3.110 https://doi.org/10.1186/s40850-017-0013-2).

ACTION: We thank the reviewer for highlighting these two interesting manuscripts on comparative genomics of the genus *Caenorhabditis*, which support two of the main observations of our manuscript. We now cite these two in the text (lines 152-153 and lines 166-167).

Extended data Figure 4 panel g illustrates the lack of conserved kmers in the 5' UTRs of the transcripts, not genome-derived genes but this is not made clear (other panels are derived from the genome annotation).

ACTION: We now clarify this in Extended Data Figure 4 legend (line 712).

In Fig 2 the meaning of several sets of numbers is not explained, especially evident (after much magnification) in panel d. The legends need to describe these numbers explicitly.

ACTION: We have increased the size of the panels in Figure 2 after moving the data on gene gain/loss to the new Figure 3. We have also amended its figure legend to describe all sets of numbers displayed in the figure (lines 593-607).

The synteny analysis is fascinating and nice to see. Do the authors know the karyotype of Dimorphilus?

ACTION: We thank the reviewer for his positive evaluation of the synteny analysis. *Dimorphilus gyrociliatus* karyotype is indeed known and it consists of 12 chromosomes (n = 12). Sex determination is chromosomal and syngamic of the XX (female) – Xo (male) type. We now include this information in the main text (line 84).

The analysis of conserved genetic repertoire in the face of gene loss and genome compaction is indeed striking, and the Hox cluster analyses are particularly interesting. Can the authors rule out rapid

temporal collinearity in a rapidly developing embryo (ie the possibility that the temporal cadence has been compressed to span a short period not sampled by their analysis)?

ACTION: We thank the reviewer for raising this interesting point. *Dimorphilus* embryonic development lasts 6-7 days, which is relatively slow when compared to other annelid species. For instance, *Dimorphilus* takes around 3 days from zygote to gastrula, while this happens in just seven hours in *Owenia fusiformis* (a member of the Palaeoannelida), in ~12 hours in *Platynereis dumerilii* and in ~24 hours in *Capitella teleta,* at comparable temperatures. Given the slow pace of *Dimorphilus gyrociliatus* development, it is thus unlikely that temporal collinearity has been compressed and occurs rapidly at an early gastrula stage. We now include this point in the main text (lines 210-213).

"our data suggest a link between gene loss, genome compaction and morphological miniaturisation in Dimorphilus, previously unrecognised in any other free-living animal with a miniature genome". Were these not also the conclusions from the Oikopleura analyses?

ACTION: We agree that as phrased, this conclusion is indeed similar to what occurs in *Oikopleura*, yet as our data shows, genome compaction and perhaps morphological miniaturisation has followed very different evolutionary trajectories in *Dimorphilus* and *Oikopleura*. To avoid any misunderstanding with the *Oikopleura* data, we have removed the last part of the sentence.

General comment

The figures as constituted I presume are expected to be on an "A4" page. If this is the case, some of the text will be impossible to read without a magnifying glass (and comparing data on mapped figures near impossible). The authors should decide which panels are essential and move the others to Supplemental information, perhaps.

ACTION: Benefitting from the extended article format allowed by the journal, we have now created two main new figures (Figure 5 and Figure 6), which allow us to present the main essential data in a larger format, thus addressing the reviewer's concerns with small font sizes in some of the panels. New Figure 3 focuses on patterns of gene gain/loss, Hox gene expression and macrosyntenty, while the data on the MYC pathway are now presented in Figure 4. ATAC-seq and CAGE-seq data are in Figure 5 and conclusion/summary panels are in Figure 6.

Reviewer #2 (Remarks to the Author):

Comments to Authors

In this manuscript, Martín-Durán et al. sequenced, assembled, and analyzed the genome of Dimorphilus gyrociliatus, a meiobenthic, miniaturised annelid that lays claim to having the second-smallest genome encoded yet for a free-living animal. The effort expended in this manuscript represents a tour-de-force that examines this genome using state of the art sequencing technologies (e.g. PacBio long reads, ATAC-seq, CAGE-seq) in addition to applying a large suite of standard analyses to the genome assembly. The Dimorphilus genome shows high levels of conservation in terms of gene content and synteny, including a well-preserved and ordered gene Hox cluster. This is striking, and deviates from what we have come to expect from highly compacted genomes. ATAC-seq, and CAGE-seq provided a view of the regulatory

landscape of the Dimorphilus genome revealing elements similar and divergent relative to other bilaterian genomes.

The striking conservation of gene content and synteny despite reduced genome size is an important finding that will be of interest to a wide audience. This genome also adds to a paucity of genome data from a highly speciose but understudied phylum. The many datasets generated for this study will also be a tremendous resource for other researchers in the fields of phylogenomics and evolutionary developmental biology. The analyses and writing are of high quality, and are extensive. Most of my comments relate to some premature conclusions the authors make based on the data presented. These conclusions are highly speculative and the authors should be deliberate in making it clear that these ideas are hypotheses that need to be tested (much of which is not possible in the near term). Further, there were instances of very dense figures with several analyses that ended up being summarized in one sentence. These would benefit from a little more elaboration.

RESPONSE: We thank the reviewer for the positive appraisal of our manuscript. We have followed the suggestions that this and the other reviewers put forward to make the text less speculative and more focused on particular findings.

Major Comments

Hox cluster analysis and expression

The claim in Figure 3a legend that "Whole mount in situ hybridisation during embryogenesis reveals that Hox genes exhibit spatial, but not temporal collinear expression domains" or line 196 "Hox genes show only spatial collinearity" is not clear from the figure. The panel is extremely small, and not enough information is provided to the reader about this poorly-studied species' embryos to be able to interpret the data. To assess if the domains are collinear, one would need double in situ hybridizations or need very clear landmarks observable in the embryo that show that the gene order corresponds to the localization of expression patterns along the AP axis. The statement in the text "Hox genes are expressed in staggered anteroposterior domains along the developing trunk," is more conservative, and is well-supported by the figure as presented.

ACTION: We agree with the reviewer that double *in situ* hybridization would help to dissect the exact spatial relationships between Hox gene expression. Unfortunately, this protocol is not established in *Dimorphilus* embryos yet. We have thus followed the reviewer's recommendation and toned down the text, referring to staggered anteroposterior domains of Hox gene expression instead of spatial collinearity (lines 217 and line 617). In addition, and following reviewer #1 comments, we have increased the size of the Hox *in situ* hybridisation panel in the newly formatted Figure 3 and added dotted lines to demarcate the head/trunk boundary in elongating embryos, which aids in comparing spatial domains of expression along the anteroposterior axis.

Growth/Proliferation pathway analysis

Why were the kynurenine and Myc pathways chosen as a way to assess growth/proliferation pathways? Was there a systematic/unbiased analysis that showed that proliferation/growth-associated pathways were more likely to show divergence or gene loss relative to other pathways? To assess how meaningful the loss of genes or changes in amino acid residues in these pathways is, it is important to know whether

Dimorphilus has done away with all pathways that regulate these processes. For example, was the Hippo pathway considered? What about Akt, mTOR? Without a full understanding of other growth/proliferation pathways, the statement in the Discussion "Dimorphilus exhibits losses and genetic modifications of metabolic (kynurenine pathway) and cellular (Myc) pathways involved in growth, proliferation, and senescence, and that when deregulated cause genomic instability, but also hypoplasia, dwarfism, and premature aging." currently reads as though these pathways have been cherry-picked for analysis.

RESPONSE: We thank the reviewer for highlight this point. Using genome wide KEGG and PFAM annotations, we indeed checked other pathways involved in growth and proliferation (Hippo, mTOR, AKT, cyclins, etc), but they are all apparently intact, at least with regards to presence/absence of core components. It was with this approach that we identified the MYC_N transactivating domain (PF01056) as being missing or heavily modified in *Dimorphilus* compared to *Capitella*, which led us to analyse the MYC pathway in these organisms in more detail.

ACTION: We have made a new Supplementary Table 10 reporting presence/absence of core components of the Hippo, PI3K/AKT/mTOR pathway and cyclins/CDKs and a new Figure panel showing the conservation of these elements in *Dimorphilus* and their connection to MYC. In addition, we have re-written the paragraph on growth/proliferation to clarify how we identified MYC and toned down our interpretations of these findings (lines 234-244). Given the new findings on the phylogenetic distribution of the kynurenine pathway (see point below to reviewer #3) we have excluded this data from the revised version of the manuscript.

Further, the authors should temper their statement "these genomic changes indicate the first known mechanistic link between genome size reduction and organism miniaturisation, suggesting Myc and sirtuins as potential candidates connecting genome size with metabolism30, developmental rate31, and body/cell size1,32." Since the identification of a mechanistic link would require functional studies, this is better written as "these pathways provide a substrate for studying whether there is a mechanistic link between genome size reduction and organism miniaturisation…"

ACTION: We have modified the sentence as suggested by the reviewer (lines 322-323).

CTCF and regulatory biology

The finding that CTCF binding sites are highly enriched in promoter regions is interesting. However, is an ortholog of CTCF encoded in the genome of Dimorphilus? I could not find this information easily in the manuscript.

ACTION: *Dimorphilus* genome encodes for a CTCF ortholog. To make this point clearer, we have confirmed the orthology of the *Dimorphilus* CTCF (Supplementary Figure 5) and amended the text accordingly (lines 268-272).

Further, the reduced intergenic space in this genome, together with the enrichment of CTCF sites in promoters actually suggests that the role of CTCF in regulating the genome is likely quite different from its role in vertebrates, where it tends to be present far from transcriptional start sites and marks TAD boundaries. Therefore, the authors should re-write statements such as this: "The CTCF DNA-binding motif was the most abundant in active regulatory regions, located mostly in promoters and as single motifs (Fig. 4e; Extended Data Fig. 9e–h), supporting that, unlike nematodes with compact genomes63,

CTCF plays a major regulatory role in Dimorphilus genome, as observed in vertebrates64". The data presented show that in contrast to nematodes, which don't have a CTCF ortholog, the Dimorphilus CTCF (if present) could be playing a role in regulating gene expression. But this role is likely quite different from its function in vertebrates. The sentence, as written, can be interpreted as suggesting that regulatory roles between vertebrates and Dimorphilus are shared.

ACTION: We now clarify this section as suggested by the reviewer (lines 268-272).

Absence of long-range regulation and GRNs

In lines 278-282, the authors suggest that the absence of long-distance gene regulation would imply simplification or dismantling of gene regulatory networks. I don't understand the logic behind this. GRNs describe regulatory relationships of transcription factors and their target genes, which could be mediated via proximal regulatory elements. The promoter regions of Dimorphilus genes could be chock full of binding sites and the animal could have many GRNs, be they conserved or lineage-specific. If the genome compaction was so good at retaining gene families by trimming out paralogs, it could also have been great at keeping essential regulatory relationships intact, just bringing the regulatory information closer together. Since the authors don't have data that can assess GRNs, they should reconsider this inference/suggestion.

ACTION: We agree with the reviewer's concern and we now clarify, and tone done this inference (lines 305-312).

Extended data figure 8 (life cycle transcriptome sequencing)

This figure represents analysis of a large amount of RNA sequencing data from different life history stages. Extensive analysis is described in the supplementary info (section 3.3). However, the only reference to this figure in the main text was in the context of Hox gene expression (line 189: Stage-specific transcriptomics (Extended Data Fig. 8) and gene expression analyses during Dimorphilus life cycle indicate that all Hox genes butHox5 and Antp become simultaneously transcribed in early development, after gastrulation). The Figure was too low resolution to be able to see what the volcano plots were showing, and I did not find a supplemental file (e.g., spreadsheet of differentially expressed genes) associated with this. The authors should assess whether releasing these data as part of this manuscript is necessary, or they should explain the figures shown. Without that, this impressive-seeming work fails to be meaningful for the community.

ACTION: We agree with the reviewer that there was limited mention to the stage-specific RNA-seq data in the original version of the manuscript. We believe however that releasing this data with this manuscript will benefit the broader community, and it contributes to support key messages of our work, such as the lack of temporal collinearity in Hox genes. Therefore, we now include Supplementary Files 1–3 with a list of differentially expressed genes and provide an explanation of Extended Data Figure 8 in the main text (lines 197-205).

Minor Comments

- line 246: "our data indicates that Dimorphilus has retained a conserved genomic regulatory profile". The authors should clarify in this statement what they mean by "genomic regulatory profile", particularly

because the subsequent phrases point out how the Dimorphilus genome deviates from other bilaterian genomes.

ACTION: We have amended the sentence accordingly (lines 272-275).

- It would be helpful to make the index at the beginning of the supplementary info file more detailed. e.g, listing what is contained in the supplementary tables, and a bit more detail for the data files.

ACTION: We now include a detailed index for Supplementary Information.

Reviewer #3 (Remarks to the Author):

This is a solid paper reporting the genome of a miniature annelid, Dimorphilus gyrociliatus. The animal is interesting, the genome quality is good, the analysis is, for the most part, sound and the paper is reasonably clear. Of particular note, the work goes beyond a gene inventory and includes study of regulatory structure via ATAC-seq analysis. My reservations are centred around some points that I think the authors' have stretched too far.

RESPONSE: We thank the reviewer for the positive evaluation of our manuscript. We have now addressed all the points raised below (some of them shared with reviewer #2) which we believe tone down the points of concern.

Major points:

1. As a general issue, the authors need to take more care not to conflate the 'miniature annelid' and the 'small genome' stuff, particularly when talking about mechanism, unless they are explicitly meaning to link the two things. I am sure they are clear in their minds about this, but as an uninitiated reader I kept having to stop to think about it. An explicit evidence-based statement in the introduction about whether the authors think that a link between genome size and body size is tenable might help clarify later sections (they appear to be chasing this hypothesis).

ACTION: We agree with the reviewer that it is important to differentiate body size evolution from changes in genome size. Correlative studies linking both parameters generally suffered from limited taxon sampling (hence multiple exceptions have thereafter been discovered) and a lack of mechanistic propositions. In this regard, evidences suggest there is not a link between genome size and body size applying to Metazoa broadly, yet it might be that these two parameters co-evolve in certain lineages of the animal tree of life (e.g. some flatworms, copepods, aphids, etc). We now clarify this issue in the introduction (line 65-73).

Lines 212 to 231 are poor in this regard. In essence what are the hypotheses? Do they think there is a direct connection, with genome size causing body size? Are these things manifestations of a single phenomenon, or two different ones?

ACTION: We have rewritten this paragraph and we now state more clearly our hypothesis that if body size and genome size are connected, we would expect to see divergences in the molecular machinery controlling cell/body size and genome stability in *Dimorphilus* (lines 234-256).

2. One of the abstract headlines, the kynurenine pathway has some issues. How was this pathway identified? Given that this is a global analysis, was this the only such pathway identified (notwithstanding

the other genes mentioned)? What were the criteria for choosing? In essence, can we be confident that this is one of the only pathways affected?

ACTION: As indicated to reviewer #2 (see point above), we used genome wide KEGG and PFAM annotations to identify missing genes and protein domains in *Dimorphilus*, which led us to identify the divergences in MYC and kynurenine pathway. We also screened other pathways involved in cell proliferation, cell size and body size control, but they appear intact. Due to text length limitations, we originally kept this data out of the manuscript, but given the reviewers' concerns, we have now included the data in the main text (lines 234-244) and as a main panel figure (Figure 4a) and Supplementary Table 10. Based on the data provided in the point below, we have now excluded our findings on the Kynurenine pathway from the text.

3. The kynurenine pathway is problematic in that several of these genes appear to be absent in Drosophila (searching with human KYNU, HAAO, QPRT). Drosophila is fairly small, and has a fairly small genome - do the authors think this is a data point that is consistent with their broader hypothesis? Why don't they mention it? What about other animals (small and large genomes and bodies)?

ACTION: We thank the reviewer for raising this important point. We have explored the presence/absence of the Kynurenine pathway across the 28 animal genomes used in our manuscript, which cover all major metazoan lineages (Table 1 below). We found four main instances with divergent Kynurenine pathways, one being *Dimorphilus*. The other three cases are *Drosophila* – as the reviewer points out – and the two other arthropod lineages (*Strigamia* and *Tribolium*), *Octopus* (Mollusca) and *Mnemiopsis* (Ctenophora). The case of cephalopods – and *O. bimaculoides* in particular – was surprising, as they do have large genomes. Employing MolluscDB, we confirmed that KYNU is absent from any of the cephalopods included in this genomic/transcriptomic database, and thus the absence of KYNU in *O. bimaculoides* is likely not a mistake during genome annotation. Moreover, it seems insects cannot generate NAD+ from tryptophan generally (https://doi.org/10.1016/S0065-2806(08)60130-7), as they lack QPRT. Given the broad range of genome sizes in insects, it is unlikely the lack of this metabolic resource has had an impact on genome size variation in this clade. Therefore, our suggestion that the loss of KYNU could promote genome instability/compaction is not supported, and we have removed this information from the manuscript.

Table 1 – Presence/absence of Kynurenine pathway across Metazoa

Species	Genome Size (Gb)	TDO	IDO	AFMID	KYNU	KMO	HAAO	QPRT
A. queenslandica	0.167	N	Y	Y	Y	Y	Y	Y
M. leidyi	0.15	Y	N	N	N	N	N	N

N. vectensis	0.34	Y	Y	Y	Y	Y	Y	Y
S. kowalevskii	0.758	Y	Y	Y	Y	Y	Y	Y
B. lanceolatum	0.59	Y	Y	Y	Y	Y	Y	Y
C. intestinalis	0.2	Y	N	Y	Y	Y	Y	Y
O. dioica	0.07	N	Y	Y	Y	Y	Y	N
L. oculatus	1.4	Y	Y	Y	Y	Y	Y	Y
H. sapiens	3.1	Y	Y	Y	Y	Y	Y	Y
C. elegans	0.103	Y	N	Y	Y	Y	Y	N
P. pacificus	0.17	Y	Y	Y	Y	Y	Y	N
R. varieornatus	0.055	Y	N	Y	Y	Y	Y	N
S. maritima	0.29	Y	N	N	Y	N	Y	Y
D. melanogaster	0.17	Y	N	Y	N*	Y	N	N
T. castaneum	0.21	Y	N	Y	N	Y	N	N
A. vaga	0.244	Y	Y	Y	Y	Y	Y	Y
M. lignano	0.742	Y	Y	Y	Y	Y	Y	N
S. mediterranea	0.72	Y	N	Y	Y	Y	Y	Y
O. bimaculoides	2.93	Y	N	Y	N	Y	N	Y
L. gigantea	0.43	Y	Y	Y	Y	Y	Y	Y
C. gigas	0.637	Y	Y	Y	Y	Y	Y	Y

M. yessoensis	0.988	Y	Y	Y	Y	Y	Y	Y
D. gyrociliatus	0.072	Y	N	Y	N	Y	N	N
C. teleta	0.324	Y	Y	Y	Y	Y	Y	Y
H. robusta	0.24	Y	N	Y	Y	Y	Y	Y
N. geniculatus	0.859	Y	Y	Y	Y	Y	Y	Y
P. australis	0.498	Y	Y	Y	Y	Y	Y	Y
L. anatina	0.41	Y	Y	Y	Y	Y	Y	Y

What about the status in other animals of the other genes and pathways that they mention?

ACTION: As we show on Figure 4c, the divergences in MBII transactivating domain of MYC are unique to Dinophilidae, while this domain is extremely conserved across Metazoa, with the exception of Ctenophora. Loss of either MNT or MAD is more common, but *Dimorphilus* and *Oikopleura* are the only two lineages of our extensive dataset lacking both genes (new Supplementary Table 11).

Other points:

L.51 - 'unlike animals with small genomes' - probably should be 'unlike other animals...'

ACTION: Changed as suggested.

L.140 - not clear what the authors mean by 'very short introns' when in the same sentence these introns are not shorter "than in Capitella and even the centipede".

ACTION: We now provide the median intron size of *Dimorphilus*, human, *Capitella* and *Strigamia* as comparison (lines 144-146).

L.141 - I don't see how a metric that measures % intron retention shows that one species or another had 'adapted' its splicing to deal with short introns. The authors seem to be arguing that the human splicing machinery is not adapted to removing human introns, or that Dimorphilus isn't adapted to removing its own long introns.

ACTION: We have clarified this sentence to describe that splicing occurs more efficiently with shorter introns than larger introns in *Dimorphilus*, as expected by its generally short intron sizes (whereas the opposite is found in humans; line 144).

L.151 "we first reconstructed gene families". It really isn't clear what this means. Were other species included? "80% of Dimorphilus genes are assigned to shared gene families" - what does 'shared' mean? The main text should at least have a basic outline of this.

ACTION: Shared gene families means multi-species orthogroups. We now clarify these points in the main text (lines 155-159).

L.157 "Capitella which is the most conservative sequenced annelid genome" ref 48 can't support that statement as most annelids have been sequenced since that was published. I'm not aware of any published work on this.

ACTION: We have rewritten this sentence to address the reviewer's concern (line 162-163).

L.160 'gene family streamlining' - as an observation, genes don't know what family they're in, so in itself this doesn't make sense from a mechanistic point of view.

ACTION: We now clarify this issue following the nomenclature used in the references suggested by reviewer #1 for this particular point (line 165-167).

L.205, Macrosynteny - legend to Figure 3b/S7f should explain what is being shown and why it supports the argument of the main text. For example, what does the color scheme represent? The resolution of these figures should be improved.

ACTION: Macrosynteny analyses are now in Figure 4c and Extended Data Figure 7f. This has allowed us to increase the size of the plots in the main Figure 4. In addition, we have expanded figure legends generally to describe all variables in the panels. Given space restrictions, we could not expand Extended Data Figure 7f panel.

L.293 'the first known mechanistic link between genome size reduction and organism miniaturisation'. I don't understand what the authors are claiming here. 'known' implies some sort of consensus or acceptance. Wouldn't 'a possible mechanistic link' be more appropriate? They are far away from being able to state that one single mechanism causes both genome size reduction and body size reduction. This needs to be rewritten making clearer what the results are and what is the authors' interpretation.

ACTION: Following reviewer's comments, we have toned down this sentence as per reviewer #2 suggestion (lines 320-324).

L.683 "1980'ies" -> "1980s"

ACTION: Corrected as suggested.

Decision Letter, first revision:

10th August 2020

*Please ensure you delete the link to your author homepage in this e-mail if you wish to forward it to your co-authors.

Dear Andreas,

Your revised manuscript entitled "Conservative route to extreme genome compaction in a miniature annelid" has now been seen by our reviewers, and in the light of their advice I am delighted to say

that we can in principle offer to publish it. First, however, we would like you to revise your paper to address the final points made by the reviewers, and to ensure that it is as brief as possible and complies with our Guide to Authors at http://www.nature.com/natecolevol/info/final-submission.

I should stress that we agree with Reviewers #1 and 2 that you must abandon the speculation linking genome reduction and body size miniaturisation.

TRANSPARENT PEER REVIEW
Nature Ecology & Evolution offers a transparent peer review option for new original research manuscripts submitted from 1st December 2019. We encourage increased transparency in peer review by publishing the reviewer comments, author rebuttal letters and editorial decision letters if the authors agree. Such peer review material is made available as a supplementary peer review file. Please state in the cover letter 'I wish to participate in transparent peer review' if you want to opt in, or 'I do not wish to participate in transparent peer review' if you don't. Failure to state your preference will result in delays in accepting your manuscript for publication.
Please note: we allow redactions to authors' rebuttal and reviewer comments in the interest of confidentiality. If you are concerned about the release of confidential data, please let us know specifically what information you would like to have removed. Please note that we cannot incorporate redactions for any other reasons. Reviewer names will be published in the peer review files if the reviewer signed the comments to authors, or if reviewers explicitly agree to release their name. For more information, please refer to our FAQ page.

SPECIFIC POINTS:
In particular, while checking through the manuscript and associated files, we noticed the following specific points which we will need you to address:

1. A one-sentence editorial summary of the paper will appear on the journal homepage with the link to the paper. This is our proposed summary: 'This study reports the genome of the miniature segmented annelid Dimorphilus gyrociliatus and reveals no drastic changes in genome architecture and regulation, unlike other cases of genome miniaturisation.' Please let us know of any factual inaccuracies.

2. Please include a full Methods section in the main article (max 3,000 words).

3. We try to avoid claims of novelty. Please remove 'novel' from the last sentence in the Abstract.

4. Please make custom code available in a public repository rather than it being available on request and change the code availability statement accordingly.

5. Please make the ENA accession code active now and ensure that you include raw reads and the assembled genome.

6. Please complete the attached Inventory of supporting information and upload it to our system.

7. Please could you revise the colour scheme in your figures so that it is readable by people who are red-green colourblind. See here for recommendations on Figure presentation: https://www.nature.com/natecolevol/info/final-submission. (for example, you could change Red to

Magenta).

8. Please include scale bars in the images of Figure 3.

9. Please complete the Editorial policy checklist and the Reporting Summary (links below) and upload them with your revised manuscript. We will publish the latter along with the paper. Please note that these forms are dynamic ‘smart pdfs’ and must therefore be downloaded and completed in Adobe Reader. Please also ensure that “Final Submission” box is checked.

Editorial policy checklist: https://www.nature.com/authors/policies/Policy.pdf

Reporting summary: https://www.nature.com/authors/policies/ReportingSummary.pdf

GENERAL POINTS:
We will also need you to check through all of the following general points when preparing the final version of your manuscript:

The main manuscript file should include the abstract, main text, methods, author contribution, data availability, code availability and competing interests statements, acknowledgements, references, and figure legends. Figures should be submitted separately as individual files. For details on other supporting material, please see below.

Figures:
Choosing the right electronic format for your figures at this stage will speed up the processing of your paper. We would like the figures to be supplied as vector files - EPS, PDF, AI or postscript (PS) file formats (not raster or bitmap files), preferably generated with vector-graphics software (Adobe Illustrator for example). Please try to ensure that all figures are non-flattened and fully editable. All images should be at least 300 dpi resolution (when figures are scaled to approximately the size that they are to be printed at) and in RGB colour format. Please do not submit Jpeg or flattened TIFF files. Please see our guidelines https://www.nature.com/documents/NRJs-guide-to-preparing-final-artwork.pdf for more details, and also our image policies http://www.nature.com/authors/editorial_policies/image.html.

We will edit your figures/tables electronically so they conform to Nature Ecology & Evolution style. If necessary, we will re-size figures to fit single or double column width. If your figures contain several parts, the parts should be labelled lower case a, b, and so on, and form a neat rectangle when assembled.

Figure legends must provide a brief description of the figure and the symbols used, within 350 words. This must include definitions of any error bars employed in the figures.

Should your Article contain any items (figures, tables, images, videos or text boxes) that are the same as (or are adaptations of) items that have previously been published elsewhere and/or are owned by a third party, please note that it is your responsibility to obtain the right to use such items and to give proper attribution to the copyright holder. This includes pictures taken by professional photographers and images downloaded from the internet. If you do not hold the copyright for any such item (in whole or part) that is included in your paper, please complete and return this <a

href="http://www.nature.com/documents/thirdpartyrights-origres.doc">Third Party Rights Table, and attach any grant of rights that you have collected.

Please check the PDF of the whole paper and figures (on our manuscript tracking system) VERY CAREFULLY when you submit the revised manuscript. This will be used as the 'reference copy' to make sure no details (such as Greek letters or symbols) have gone missing during file-transfer/conversion and re-drawing.

Supporting Information:
All Supporting Information must be submitted in accordance with the instructions in the attached Inventory of Supporting Information, and should fit into one of two categories:

1. EXTENDED DATA: Extended Data are an integral part of the paper and only data that directly contribute to the main message should be presented. These figures will be integrated into the full-text HTML version of your paper and will be appended to the online PDF. There is a limit of 10 Extended Data figures, and each must be referred to in the main text, cited as Extended Data 1, Extended Data 2, etc. Each Extended Data figure should be of the same quality as the main figures, and should be supplied at a size that will allow both the figure and legend to be presented on a single A4 page. Each figure should be submitted as an individual .jpg, .tif or .eps file with a maximum size of 10 MB each. All Extended Data figure legends must be provided in the attached Inventory of Supporting Information, not in the figure files themselves.

2. SUPPLEMENTARY INFORMATION: Supplementary Information is material that is essential background to the study but which is not practical to include in the printed version of the paper (for example, video files, large data sets and calculations). Each item must be detailed in the attached Inventory of Supplementary Information. Tables containing large data sets should be in Excel format, with the table number and title included within the body of the table. All textual information and any additional Supplementary Figures (which should be presented with the legends directly below each figure) should be provided as a single, combined PDF. Please note that we cannot accept resupplies of Supplementary Information after the paper has been formally accepted unless there has been a critical scientific error.

Additional Supplementary Figures and other items are not required to be referred to in your manuscript text (though they can be), but should be numbered as Supplementary Figure 1, not SI1, etc.

Methods & Notes:
Please include references for the Methods in the same list as those for the main text, following on sequentially after the main text references. Any citations in the Supplementary Information will need inclusion in a separate SI reference list.

Please include a data availability statement as a separate section after Methods but before references, under the heading "Data Availability”. This section should inform readers about the availability of the data used to support the conclusions of your study. This information includes accession codes to public repositories (data banks for protein, DNA or RNA sequences, microarray, proteomics data etc...), references to source data published alongside the paper, unique identifiers such as URLs to data repository entries, or data set DOIs, and any other statement about data availability. All data that support the findings of the study must be made available. If DOIs are provided, we also strongly

encourage including these in the Reference list (authors, title, publisher (repository name), identifier, year). For more guidance on how to write this section please see:
http://www.nature.com/authors/policies/data/data-availability-statements-data-citations.pdf

Nature Research policies (https://www.nature.com/authors/policies/availability.html#data) include a strong preference for research data to be archived in public repositories and in some cases this is mandatory. If you need help complying with this policy, or need help depositing and curating your research data (including raw and processed data, text, video, audio and images) you should consider:

Contacting Springer Nature’s Research Data Helpdesk (https://www.springernature.com/gp/authors/research-data-policy/helpdesk/12327114) for advice.
Finding a suitable data repository (https://www.springernature.com/gp/authors/research-data-policy/repositories/12327124) for your data.
Uploading your data to Springer Nature’s Research Data Support service (https://springernaturedata.typeform.com/to/UeGGKT). Please note there are fees (https://www.springernature.com/gp/authors/research-data-policy/pricing/15499842) for using Springer Nature’s Research Data Support service.

Finally, we require authors to include a statement of their individual contributions to the paper, such as experimental work, project planning, data analysis, etc., immediately after the acknowledgements. The statement should be short, and refer to authors by their initials. For details please see the Authorship section of our joint Editorial policies at
http://www.nature.com/authors/editorial_policies/authorship.html

Please resubmit electronically

* the final version of the text (not including the figures) in either Word or Latex.

* publication-quality figures. For more details, please refer to our Figure Guidelines, which is available here: https://www.nature.com/documents/NRJs-guide-to-preparing-final-artwork.pdf .

* any Extended Data and Supplementary Information, as per instructed, with
the associated Inventory document.

* copies of our reporting and editorial policy checklists even if they have not changed since the previous round of revision.

* a point-by-point response to any issues raised by our reviewers and to any editorial suggestions.

* any suggestions for cover illustrations, which should be provided at high resolution as electronic files. Please note that such pictures should be selected more for their aesthetic appeal than for their scientific content. I am sure you will understand that we cannot make any promise as to whether any of your suggestions might be selected for the cover of Nature Ecology & Evolution.

Please use the following link to access your home page:

[REDACTED]

*This url links to your confidential homepage and associated information about manuscripts you may have submitted or be reviewing for us. If you wish to forward this e-mail to co-authors, please delete this link to your homepage first.

Please also send the following forms as a hand-signed PDF by email to ecoevo@nature.com.

*Please sign and return the Licence to Publish form

Or, if the corresponding author is a Crown government employee (including Great Britain and Northern Ireland, Canada and Australia), please sign
and return the Licence to Publish form for Crown government employees , or the Licence to Publish form for US government employees

For more information on our licence policy, please consult http://npg.nature.com/authors.

AUTHORSHIP

CONSORTIA -- For papers containing one or more consortia, all members of the consortium who contributed to the paper must be listed in the paper (i.e., print/online PDF). If necessary, individual authors can be listed in both the main author list and as a member of a consortium listed at the end of the paper. When submitting your revised manuscript via the online submission system, the consortium name should be entered as an author, together with the contact details of a nominated consortium representative. See https://www.nature.com/authors/policies/authorship.html for our authorship policy and https://www.nature.com/documents/nr-consortia-formatting.pdf for further consortia formatting guidelines, which should be adhered to prior to acceptance.

Nature Research journals encourage authors to share their step-by-step experimental protocols on a protocol sharing platform of their choice. Nature Research's Protocol Exchange is a free-to-use and open resource for protocols; protocols deposited in Protocol Exchange are citable and can be linked from the published article. More details can found at www.nature.com/protocolexchange/about.

We hope to hear from you within two weeks; please let us know if the revision process is likely to take longer.

[REDACTED]

****

Reviewer Comments:

Reviewer #1 (Remarks to the Author):

General comments

The paper is much improved. The core claim, of the exceptionality of the Dimorphilus genome, and especially the fact that it is small next to other annelid genomes, remains foregrounded. The authors also maintain links to "explanations" or "mechanisms" for this miniaturisation that suffer from a critical n=1 issue common in analyses of unique or rare phenotypes: it is not possible to separate cause, consequence and chance association. What do they take as the "base" genome size for annelids? The mode of those with estimates is ~350 Mb (see histogram attached), and there are diverse other annelids with genomes in the <200 Mb range (Tharyx multibranchis, Parapionosyllis elegans, Sphaerosyllis pirifera, Polyophthalmus pictus). That other annelids have similarly (though not quite as) small genomes suggests that some careful modelling is required. It is true from the data that Dimorphilus has a relatively normal gene complement (albeit reduced in absolute size: most functions are present), has retained ancestral synteny and has retained a distinct subset of the genome regulatory systems present in animals with larger genomes. This is interesting and offers, as the authors point out, a counter to the miniaturisation / simplification / reorganisation pattern observed in some other taxa. The authors might like to consider Sydney Brenner's favourite model - fugu: a 600 Mb genome that is functionally equivalent to the 1.2 Mb genomes of other osteichtyhes, and possibly of other vertebrates (eg Homo at 3 Gb). There too "structure" and "regulation" are conserved...

Overall I think the richness of the data argues for publication, but the "exceptionalism" line of the core argument is not well contextualised/supported.

best
Mark Blaxter

Commentary details

Response to Reviewers
the k-mer profile for Dimorphilus is unusual
Yes it is, as expected from the kmer abundance plot. Can the authors affirm that the high coverage in GC-richer sequence really is an artifact of sequencing. The difference (going from an apparent GC% of 8/31 to 10/31 - 28% to 32%) is not particularly large, and if true of these data one would expect to see it in other similar datasets where the genome GC is ~305. Is that the case? The difference in coverage (100x to 350x) is higher than I have observed in analysing similarly ATrich genomes, and doesnt fit the published biases in Illumina technology. What if these high abundance AT-poorer reads really are high abundance repeat units? Is there a pattern of GCricher vs GC poorer isochores in the assembly? [it is entirely unsurprising that digital normalisation to 100x yields a coverage peak at ... 100x]

ABSTRACT
extreme lifestyles - well, a third of all described eukaryotic species are parasites: if this is what is meant, this is hardly extreme (or only extreme to long-lived, mesothermal, macrofaunal primates such as ourselves). To us being an infaunal meiobiont is extreme...
73.8 Mb long - the "long" is superfluous (also line 101)
morphologically miniature meiobenthic - well if you are in the meiobenthos you are by definition small enough to go through 1 mm sieves, so "morphologically miniature" is superfluous

line 45, 48, 50 - the definite article is required, and I would suggest the name should not just be the genetic epithet - so "the D. gyrociliatus..." in all cases through the MS
Unlike other animals with small genomes - well, unlike some other: no tSL/operons in Tardigrada
gene dense Dimorphilus genome presents a divergent Myc pathway - this is two themes conflated in one sentence: the gene density and likely retention of gene-proximal regulation, and the discovery of a myc pathway that may be associated with body size change
suggesting a possible mechanistic relationship between genome size reduction and morphological miniaturisation - as discussed by other reviewers, this is not really bourne out by the single instance of Dimorphilus and is countered by so many examples that the "mechanistic" claim is specious

MAIN TEXT
[[throughout - place space between value and unit]]
line 57: and gene content, which has been - Needs to be two sentences - and gene content. This has been
line 58: often increase in size mobilising their - this suggests an active "choice" to mobilise TEs, which is not the case; genomes can expand in size through expansion of TE that likely is accompanied by escape from genomic surveillance of said TEs; whether the change in genome size is adaptive per se and not just a neutral or nearly neutral consequence of TE escape is not known
line 60: e.g. in vertebrates and teleosts7-9, as well as in insects10 - teleosts are vertebrates,
line 71: nor the insect with arguably smallest body size has a compact genome - this is rather mangled... ands the insect with the smallest body size does not have a compact genome perhaps?
line 79: essential for drastic genomic streamlining or genomic specialisations unrelated to genome compaction. - is this a "necessary and sufficient" versus "predisposition" argument. The logic is quite obscure (it reads like a set of non-explicit assumptions is being lined up for rejection)
line 83: has a C-value - has been reported to have a C-value
line 85 - miniature free-living meiobenthic species44 - again, to be in the meiobenthos you are by definition miniature
line 88: no appendages, parapodia and chaetae - "or chaetae"
line 93: Although there are only a handful of genomes sequenced (Supp. Table 1), annelids have retained - this reads as if the retention is despite the low niumber of genomes; surely the low number of genomes have revealred that the features have retained - the although is misleading
line 104: concurring - agreeing
line 113: also exhibits a 92.47 Mb compact genome - also has a compact, 92.5 Mb genome
line 114: genome compaction - genome size reduction
line 115: the sterngth of this argument is not clear. From Figure 1, it would appear that the Errantia Parapionosyllis genome is only 20-30 Mb larger, and the Sedentaris Tharyx genome is 10-20 Mb larger, than Dimorphilus + Trilobodrilus. Can the authors formallyexclude the ancestral Errantia+Sedentaria+Dinophiliformia genome being of the order of 100 Mb? (ie the reduction is from 100 Mb or so to 78... hardly striking)
line 117: repetitive content - repeat content
line 118: we annotated the landscape of transposable elements - ? the transposable elements (what is a landscape) also line 594
line 127: [[similarity to cytomegaloviridae]] - its unclear why this is being reported. Is there biological significance to this in terms of genome size change or annelid development or...
line 130: supp tab 5: Fritillaria borealis genome is 143 Mb in INSDC; where does 91 Mb derive from?
line 143: However, although Dimorphilus shows overall very short introns (median 66 bp vs 1,545 bp in humans) and its splicing is thus more efficient at removing short intron sizes (Extended Data Fig. 4i), introns are not shorter on average than in Capitella (median 57 bp) and even similar to the

centipede Strigamia maritima (median 67 bp) - the comparison to the 3 Gb genome of H. sapiens is spurious. What is more interesting is that the reduced genome size is associated with reduction in repetitive content, reduction in intergenic content, but not (generally) in reduction in intron content, reduction in gene number and reduction in gene size (i.e.the number of introns in orthologous genes?) . Annelid introns are generally small, and it is likely that further size reduction would render them unspliceable. The statement of "efficency" of intron splicing is tautologous (if it couldnt splice it would be dead) and no data are adduced to show greater efficiency (than ? human, other annelids?).
line 156: reconstructed orthology gene - clusters of orthologous genes
line 160: Dimorphilus has the smallest average gene family size among annelids (1.63 genes/orthogroup - this is trivially derived from the reduced gene count; it is better to compare a stated set of clusters that have consistent numbers in other taxa, but reduced numbers in Dimorphilus ("for orthologous clusters where at least n species were represented, Dimorphilus had 0.x fold as many cluster members")
line 172: Besides, Dimorphilus shows - In addition, Dimorphilus shows
line 174: The GPCR complement is indeed dynamic - indeed is superfluous, maybe GPCR complement of genomes?
line 178: Thus, the conserved GPCR repertoire and the canonical neuropeptide complement (Extended Data Fig. 6c) and line 776: Contrary to other animals with compact genomes and miniaturised morphologies, such as tardigrades, nematodes and appendicularians, D. gyrociliatus has a conserved GPCR repertoire. - I do not see this from the presented data. Yes Oikopleura is odd, but nematodes, tardigrades? Where is the test of "conserved repertoire loss"? For example, Lingula (from the plot) looks ~= Ramazzottius, and Capitella has as many A as does Caenorhabditis... or is there something not plotted that suggests this is not the case..
line 185: Dimorphilus, and Dinophilidae generally, - this gets confusing, as now we are discussing presumably the other assembled genome as well, but previously all data are reported as if from the focal Dimorphilus only. If the authors have additional genomes to report and analyse this should be done explicitly; if features are shared by more than one species they are more likely to be biologically relevant, and the ongoing (?) genome size reduction in Dimorphilus compared to its sister surely involves additional ongoing reductive change?
line 227: but completely lost in Helobdella - does Helobdella have a "broken" `DSB system? If not the association between DSB function and genome order is very idiosyncratic, and hardly an argument with weight
line 235: Given the miniature body size and the compact genome of Dimorphilus - the major correlation is usually cited as being with cell size. What size are Dimorphilus cells compared to other annelid cells: has it fewer cells AND smaller cells, or just fewer cells?
line 255: our data suggest a link between gene loss, genome compaction and morphological miniaturisation in Dimorphilus. - in the text above the gene losses are not critically associated with the biology of the systems described: they are dispensible, or are regulators of systems. With other reviewers I find these findings interesting but not strong.
line 264: canonical metagene regulatory profile - I am not sure what this means (specifically "metagene" in this context)
line 265: splice leaders - spliced leaders
line 270: motives - motifs
line 273: has retained conserved genomic regulatory features - the previous text described what may be a novel mode of use of CTCF and so this phrase following immediately after is jarring: do the authors mean conserved regulatory systems deployed possibly in new ways?
line 283: with narrow promoters - short promoters? short upstream intergenic regions?
line 295: grounded in a derived phylogenomic position within Annelida - I am not sure what a derived

phylogenomic position is.
line 311: progenesis - I am unclear as to whether the authors accept progenesis as a prior or believe they have demonstrated progenesis. It is not explicitly discussed/addressed and the term only apperars in this paragraph.
line 317: nematodes - some nematodes (since nematode genomes range to 700 Mb)

line 778: PSI-BLAST cluster map of D. gyrociliatus pro-neuropeptides - what is this figure hoping to show? It is not meaningful as plotted: would a simple classification not deliver the same (uncomplicated) message?

Supp info
line 192-3: - this accuracy (to the base pair) is superfluous. I wonder whether the normalisation of the data (a 5000x repeat is mentioned) means that this size estimate from genomescope is an underestimate.
line 205: Illumina reads are bases not base pairs
line 238 and others: - space needed between value and units
line 256: To quantify transcript abundances - was this using the normalised reads (where apparent PCR dups were removed)? I note that for highly expressed loci, where the sheer abundance of transcripts means that read pairs with the same start and stop coordinates are expected by chance, this will suppress read counts and mask differences.
line 579: repeat of statement in the text

Reviewer #2 (Remarks to the Author):

The authors have addressed the majority of my concerns.

However, there are a few places where I still recommend some rewriting. The abstract states “suggesting a possible mechanistic relationship between genome size reduction and morphological miniaturisation”. The rest of the paper does not clearly address what such a mechanistic relationship might be. There is clearly an association of morphological miniaturization and genome compaction in this one species. However, there is no way to know whether one caused the other. The statement in the discussion that “we identified a reduced repertoire of extracellular signalling ligands, which could explain the limited elaboration of its segmental body” has no basis. This is a classic case of the correlation versus causation problem. Maybe the body became less elaborate for some other reason, and then the animal did away with ligands because there was less of a need for cell-cell communication. Then the authors say “it is tempting to speculate that the simplification or dismantlement of distal cis-regulation observed in Dimorphilus might be one of the factors underpinning morphological miniaturisation”. It is not at all obvious that gene loss or altered gene regulation should cause miniaturization. I urge the authors to resist this type of temptation.

This is a strong paper, and I think the authors should let the neat results shine on their own, without forcing conclusions or inferences that simply are not supported at this time.

Minor: in line 270, “motives” should be “motifs”

Reviewer #3 (Remarks to the Author):

Summary
--

I am generally happy that the authors have addressed my major concerns. In particular they have removed the section on the kynurenine pathway, which I think is the right choice. Equally they have moderated and made clearer the language regarding the link between molecular mechanisms and genome / body size. The ms as a whole contains much of interest and does not need less well supported stories.

Minor points
--

L.43 "we decode the 73.8 Mb long, compact genome" better rewritten as "we decode the compact 73.8 Mb genome ..." to avoid jarring juxtaposition of 'long' and 'compact'.

L.71 "nor the insect with arguably smallest body size has a compact genome" change to "[...], neither does the insect with arguably the smallest body size have a compact genome".

L.112 change to "a compact 92.47 Mb genome".

L.114 suggest changing to "our data suggest genome compaction and morphological miniaturisation both occurred in the lineage leading to [...]" to avoid ambiguity in interpretation of 'common' and ambiguity from singular use of 'event'.

L.149 'comparable' would be better than 'commensurable' unless the authors have intentionally chosen this.

L.172 delete 'Besides,'

L.270 change 'motives mostly close' to 'motifs, for the most part close [...]'

*******************END********************

Author Rebuttal, first revision:

Response to reviewers

We thank Prof Mark Blaxter and the two other anonymous reviewers for the positive evaluation of our revised work and their additional set of comments. We have addressed all the suggested text changes,

which we believe help to clarify particular points of the main text (reviewer #1–3) and adjust our conclusions to the data reported (reviewer #1 and #2).

Below we provide a detailed point-by-point response to each of the reviewers' concerns.

Reviewer #1 (Remarks to the Author):

General comments

The paper is much improved. The core claim, of the exceptionality of the Dimorphilus genome, and especially the fact that it is small next to other annelid genomes, remains foregrounded. The authors also maintain links to "explanations" or "mechanisms" for this miniaturisation that suffer from a critical n=1 issue common in analyses of unique or rare phenotypes: it is not possible to separate cause, consequence and chance association. What do they take as the "base" genome size for annelids? The mode of those with estimates is ~350 Mb (see histogram attached), and there are diverse other annelids with genomes in the <200 Mb range (Tharyx multibranchis, Parapionosyllis elegans, Sphaerosyllis pirifera, Polyophthalmus pictus). That other annelids have similarly (though not quite as) small genomes suggests that some careful modelling is required.

RESPONSE: We thank the reviewer for his positive appraisal of the revised version. We agree that separating cause, consequence and chance association is difficult, in particular without functional validation, and thus we have further toned down/removed any mechanistic speculation from the text (in response to reviewer #2 and the editorial comments, too). We also agree that further investigations that cover annelid diversity are essential to understand genome size evolution in this taxon. However, all studied lineages in Sipuncula, Amphinomidae, Chaetopteriformia and Palaeannelida (*Owenia fusiformis*, own unpublished data) have genomes sizes >500 Mb. Therefore, the most parsimonious working hypothesis is that the ancestral genome size for annelids (or at least for Sedentaria+Errantia+Dinophiliformia based on outgroup comparison) was in that range and that further expansions/contractions have happened in different annelid lineages.

It is true from the data that Dimorphilus has a relatively normal gene complement (albeit reduced in absolute size: most functions are present), has retained ancestral synteny and has retained a distinct subset of the genome regulatory systems present in animals with larger genomes. This is interesting and offers, as the authors point out, a counter to the miniaturisation / simplification / reorganisation pattern observed in some other taxa. The authors might like to consider Sydney Brenner's favourite model - fugu: a 600 Mb genome that is functionally equivalent to the 1.2 Mb genomes of other osteichthyes, and possibly of other vertebrates (eg Homo at 3 Gb). There too "structure" and "regulation" are conserved...

Overall, I think the richness of the data argues for publication, but the "exceptionalism" line of the core argument is not well contextualised/supported.

ACTION: We thank the reviewer for the suggestion to compare *Dimorphilus* with fugu. To contextualise better our findings, we have added this comparison in the last sentence of the abstract and in the

discussion (lines 313-314) and removed the mention to the possible mechanistic connection between genome compaction and morphological miniaturisation (also reviewer #2 suggestion).

best

Mark Blaxter

Commentary details

Response to Reviewers

the k-mer profile for Dimorphilus is unusual

Yes, it is, as expected from the k-mer abundance plot. Can the authors affirm that the high coverage in GC-richer sequence really is an artifact of sequencing? The difference (going from an apparent GC% of 8/31 to 10/31 - 28% to 32%) is not particularly large, and if true of these data one would expect to see it in other similar datasets where the genome GC is ~305. Is that the case? The difference in coverage (100x to 350x) is higher than I have observed in analysing similarly AT-rich genomes and doesn't fit the published biases in Illumina technology. What if these high abundance AT-poorer reads really are high abundance repeat units? Is there a pattern of GC-richer vs GC poorer isochores in the assembly? [it is entirely unsurprising that digital normalisation to 100x yields a coverage peak at ... 100x]

RESPONSE: We thank the reviewer for these comments and suggestions. We believe it is unlikely that the high abundance AT-poor reads are in fact high abundance repeat units, since the annotation of repeat content of our PacBio-based assembly (which is thus independent from the Illumina data) shows repeats are low (2.69% of the genome). Furthermore, the k-mer re-analysis in the previous revision showed a canonical profile and a genome size estimation well in accordance with flow cytometry, the PacBio assembly and previous literature.

ABSTRACT

extreme lifestyles - well, a third of all described eukaryotic species are parasites: if this is what is meant, this is hardly extreme (or only extreme to long-lived, mesothermal, macrofaunal primates such as ourselves). To us being an infaunal meiobiont is extreme...

ACTION: We have removed "extreme lifestyles" from the abstract.

73.8 Mb long - the "long" is superfluous (also line 101)

morphologically miniature meiobenthic - well if you are in the meiobenthos you are by definition small enough to go through 1 mm sieves, so "morphologically miniature" is superfluous

ACTION: Both points changed as suggested.

line 45, 48, 50 - the definite article is required, and I would suggest the name should not just be the genetic epithet - so "the D. gyrociliatus..." in all cases through the MS

ACTION: All instances changed as suggested.

Unlike other animals with small genomes - well, unlike some other: no tSL/operons in Tardigrada

ACTION: Changed to "Unlike some other animals with small genomes…"

gene dense Dimorphilus genome presents a divergent Myc pathway - this is two themes conflated in one sentence: the gene density and likely retention of gene-proximal regulation, and the discovery of a myc pathway that may be associated with body size change suggesting a possible mechanistic relationship between genome size reduction and morphological miniaturisation - as discussed by other reviewers, this is not really bourne out by the single instance of Dimorphilus and is countered by so many examples that the "mechanistic" claim is specious

ACTION: We have removed that last part of the sentence.

MAIN TEXT

[[throughout - place space between value and unit]]

ACTION: All instances changed as suggested.

line 57: and gene content, which has been - Needs to be two sentences - and gene content. This has been

ACTION: Changed as suggested.

line 58: often increase in size mobilising their - this suggests an active "choice" to mobilise TEs, which is not the case; genomes can expand in size through expansion of TE that likely is accompanied by escape from genomic surveillance of said TEs; whether the change in genome size is adaptive per se and not just a neutral or nearly neutral consequence of TE escape is not known

ACTION: Changed to "Animal genomes often increase in size due to the expansion of transposable elements (TE)…"

line 60: e.g. in vertebrates and teleosts7-9, as well as in insects10 - teleosts are vertebrates,

ACTION: Changed to "(e.g. in vertebrates[7-9] and insects[10])"

line 71: nor the insect with arguably smallest body size has a compact genome - this is rather mangled... and the insect with the smallest body size does not have a compact genome perhaps?

ACTION: Changed as reviewer #3 suggested.

line 79: essential for drastic genomic streamlining or genomic specialisations unrelated to genome compaction. - is this a "necessary and sufficient" versus "predisposition" argument. The logic is quite obscure (it reads like a set of non-explicit assumptions is being lined up for rejection)

ACTION: We have clarified this sentence: “Therefore, it is unclear whether these are genomic changes required for genomic streamlining or lineage specialisations unrelated to genome compaction”.

line 83: has a C-value - has been reported to have a C-value

line 85 - miniature free-living meiobenthic species44 - again, to be in the meiobenthos you are by definition miniature

line 88: no appendages, parapodia and chaetae - "or chaetae"

ACTION: We changed these three points as the reviewer suggested.

line 93: Although there are only a handful of genomes sequenced (Supp. Table 1), annelids have retained - this reads as if the retention is despite the low number of genomes; surely the low number of genomes have revealed that the features have retained - the although is misleading

ACTION: We changed the sentence to “With only a handful of genomes sequenced (Supp. Table 1), annelids have retained…”

line 104: concurring - agreeing

line 113: also exhibits a 92.47 Mb compact genome - also has a compact, 92.5 Mb genome

line 114: genome compaction - genome size reduction

ACTION: We changed these three points as the reviewer suggested.

line 115: the strength of this argument is not clear. From Figure 1, it would appear that the Errantia Parapionosyllis genome is only 20-30 Mb larger, and the Sedentaria Tharyx genome is 10-20 Mb larger, than Dimorphilus + Trilobodrilus. Can the authors formally exclude the ancestral Errantia+Sedentaria+Dinophiliformia genome being of the order of 100 Mb? (ie the reduction is from 100 Mb or so to 78... hardly striking)

RESPONSE: Applying outgroup comparison for the clade Errantia+Sedentaria+Dinophiliformia, the most probably evolutionary scenario is that the ancestral genome for the clade was large rather than small, since the known genome sizes for Sipuncula, Amphinomidae, Chaetopteriformia and Palaeannelida (own data) are >500 Mb and usually > 1 Gb.

line 117: repetitive content - repeat content

ACTION: Changed as suggested.

line 118: we annotated the landscape of transposable elements - ? the transposable elements (what is a landscape) also line 594

ACTION: We changed “landscape” to “complement”.

line 127: [[similarity to cytomegaloviridae]] – it is unclear why this is being reported. Is there biological significance to this in terms of genome size change or annelid development or...

RESPONSE: This was included in response to a point raised in the previous round of revisions.

line 130: supp tab 5: Fritillaria borealis genome is 143 Mb in INSDC; where does 91 Mb derive from?

RESPONSE: The genome size comes from DOI: 10.1016/j.cub.2019.01.080. They provide a genome size estimation of 91 Mb (Fig 1A), yet the reported assembly size is 143 Mb.

line 143: However, although Dimorphilus shows overall very short introns (median 66 bp vs 1,545 bp in humans) and its splicing is thus more efficient at removing short intron sizes (Extended Data Fig. 4i), introns are not shorter on average than in Capitella (median 57 bp) and even similar to the centipede Strigamia maritima (median 67 bp) - the comparison to the 3 Gb genome of H. sapiens is spurious.

ACTION: We have removed the comparison to human.

What is more interesting is that the reduced genome size is associated with reduction in repetitive content, reduction in intergenic content, but not (generally) in reduction in intron content, reduction in gene number and reduction in gene size (i.e. the number of introns in orthologous genes?). Annelid introns are generally small, and it is likely that further size reduction would render them unspliceable. The statement of "efficency" of intron splicing is tautologous (if it couldnt splice it would be dead) and no data are adduced to show greater efficiency (than ? human, other annelids?).

RESPONSE: Extended Data Figure 4i shows efficiency in splicing introns according to size (% of intron retention) in two species with short introns (*Dimorphilus* and *Strigamia*) and one species with large introns (human). The data demonstrate that *Dimorphilus* and *Strigamia* tend to retain (i.e. not properly splice) large introns, as the majority of introns are short, but human tend to retain shorter introns, as the majority of introns are large. We have thus kept the text as it was.

line 156: reconstructed orthology gene - clusters of orthologous genes

ACTION: Changed as suggested.

line 160: Dimorphilus has the smallest average gene family size among annelids (1.63 genes/orthogroup - this is trivially derived from the reduced gene count; it is better to compare a stated set of clusters that have consistent numbers in other taxa, but reduced numbers in Dimorphilus ("for orthologous clusters where at least n species were represented, Dimorphilus had 0.x fold as many cluster members")

ACTION: We have amended this sentence to indicate that 38.9% of the genes in *Dimorphilus* are assigned to gene families where there is only one *Dimorphilus* gene, while in the annelids *Capitella* and *Helobdella* this percentage halves (19% and 19.1% respectively). We have added this data to Supp Table 7.

line 172: Besides, Dimorphilus shows - In addition, Dimorphilus shows

line 174: The GPCR complement is indeed dynamic - indeed is superfluous, maybe GPCR complement of genomes?

ACTION: Both points changed as suggested. We removed "indeed" from that sentence.

line 178: Thus, the conserved GPCR repertoire and the canonical neuropeptide complement (Extended Data Fig. 6c) and line 776: Contrary to other animals with compact genomes and miniaturised morphologies, such as tardigrades, nematodes and appendicularians, D. gyrociliatus has a conserved GPCR repertoire. - I do not see this from the presented data. Yes Oikopleura is odd, but nematodes, tardigrades? Where is the test of "conserved repertoire loss"? For example, Lingula (from the plot) looks ~= Ramazzottius, and Capitella has as many A as does Caenorhabditis... or is there something not plotted that suggests this is not the case...

ACTION: We have added references to GPCR complements of in the text to support claims of expansions/losses. In addition, Supplementary Table 8 provides the numbers of GPCRs detected in the lineages mentioned in the text, complementing Extended Data Figure 6c.

line 185: Dimorphilus, and Dinophilidae generally, - this gets confusing, as now we are discussing presumably the other assembled genome as well, but previously all data are reported as if from the focal Dimorphilus only. If the authors have additional genomes to report and analyse this should be done explicitly; if features are shared by more than one species, they are more likely to be biologically relevant, and the ongoing (?) genome size reduction in Dimorphilus compared to its sister surely involves additional ongoing reductive change?

ACTION: This is based on the transcriptomic data used for phylogenetic analyses. We now clarify this point in Extended Data Figure 5 legend: "(dotted lines in *T. axi* and *D. vorticoides* indicate the reconstructed complements are based on transcriptomic data)".

line 227: but completely lost in Helobdella - does Helobdella have a "broken" `DSB system? If not the association between DSB function and genome order is very idiosyncratic, and hardly an argument with weight.

RESPONSE: The association between loss of DSB function, loss of linkage blocks and genome compaction comes from work on *Oikopleura dioica* (DOI: 10.1016/j.cub.2018.08.048). Our manuscript shows that genome compaction can occur while still retaining linkage blocks and DSB function. How the loss of linkage groups occurred in *Helobdella* and whether this loss is connected to an impaired DSB system is an exciting question, which however extends beyond the remit of this manuscript.

line 235: Given the miniature body size and the compact genome of Dimorphilus - the major correlation is usually cited as being with cell size. What size are Dimorphilus cells compared to other annelid cells: has it fewer cells AND smaller cells, or just fewer cells?

RESPONSE: To the best of our knowledge, there is no study comparing cell size in *Dimorphilus* versus other annelid species. This is an exciting topic for future research.

line 255: our data suggest a link between gene loss, genome compaction and morphological miniaturisation in Dimorphilus. - in the text above the gene losses are not critically associated with the biology of the systems described: they are dispensable or are regulators of systems. With other reviewers I find these findings interesting but not strong.

ACTION: We have removed that part of the sentence and moved the sentence "provide a substrate for studying whether there is a mechanistic link between genome size reduction and organism miniaturisation in *Dimorphilus*" (proposed by reviewer #2 in the previous revision) from the discussion to this point of the text.

line 264: canonical metagene regulatory profile - I am not sure what this means (specifically "metagene" in this context)

ACTION: We removed the word "metagene".

line 265: splice leaders - spliced leaders

line 270: motives - motifs

ACTION: Both points were changed as suggested.

line 273: has retained conserved genomic regulatory features - the previous text described what may be a novel mode of use of CTCF and so this phrase following immediately after is jarring: do the authors mean conserved regulatory systems deployed possibly in new ways?

ACTION: This is an interesting point. To the best of our knowledge, the ancestral role of CTCF in genome architecture in animals is still debated, and thus the condition observed in *Dimorphilus* might not necessarily be new. We now clarify the sentence in line 273: "Thus, our data indicate that *Dimorphilus* has retained conserved genomic regulatory features (e.g. lack of operons and trans-splicing, presence of CTCF) but streamlined…"

line 283: with narrow promoters - short promoters? short upstream intergenic regions?

ACTION: Changed to "short promoters".

line 295: grounded in a derived phylogenomic position within Annelida - I am not sure what a derived phylogenomic position is.

ACTION: Changed to "nested phylogenetic position".

line 311: progenesis - I am unclear as to whether the authors accept progenesis as a prior or believe they have demonstrated progenesis. It is not explicitly discussed/addressed and the term only appears in this paragraph.

RESPONSE: As stated in line 299, morphological miniaturisation in *Dimorphilus* has been classically considered a case of progenesis (e.g. DOIs: 10.1186/s12983-016-0181-x and 10.1016/j.cub.2015.06.007). We did not attempt to demonstrate progenesis, but to hypothesise that if progenesis occurred, simplification of distal *cis*-regulation might have been a driving factor. We have however removed the last sentence from this paragraph.

line 317: nematodes - some nematodes (since nematode genomes range to 700 Mb)

ACTION: Changed as suggested.

line 778: PSI-BLAST cluster map of D. gyrociliatus pro-neuropeptides - what is this figure hoping to show? It is not meaningful as plotted: would a simple classification not deliver the same (uncomplicated) message?

RESPONSE: The use of cluster maps to assign neuropeptide orthology is common in the field of neuropeptide research (see e.g. 10.1186/1471-2164-14-906 and 10.1098/rsob.170136). An annotated list (with sequences) of the neuropeptides found in *D. gyrociliatus* based on this cluster map is provided in Supp Data File 9.

Supp info

line 192-3: - this accuracy (to the base pair) is superfluous. I wonder whether the normalisation of the data (a 5000x repeat is mentioned) means that this size estimate from genomescope is an underestimate.

ACTION: We now express genome size in terms of Mb. GenomeScope2.0 size estimate is probably slightly underestimated, which is the reason why we adopt the genome size estimated by flow cytometry throughout the manuscript.

line 205: Illumina reads are bases not base pairs

line 238 and others: - space needed between value and units

ACTION: Both points changed as suggested.

line 256: To quantify transcript abundances - was this using the normalised reads (where apparent PCR dups were removed)? I note that for highly expressed loci, where the sheer abundance of transcripts means that read pairs with the same start and stop coordinates are expected by chance, this will suppress read counts and mask differences.

RESPONSE: We did not normalised RNA-seq reads. Normalisation was performed for short reads coming from DNA sequencing and only for genome size estimations.

line 579: repeat of statement in the text

RESPONSE: We could not find a repeat statement of line 579 in Supplementary Materials and main text. We left this section as originally submitted.

Reviewer #2 (Remarks to the Author):

The authors have addressed the majority of my concerns.

However, there are a few places where I still recommend some rewriting. The abstract states "suggesting a possible mechanistic relationship between genome size reduction and morphological miniaturisation". The rest of the paper does not clearly address what such a mechanistic relationship might be. There is clearly an association of morphological miniaturization and genome compaction in this one species. However, there is no way to know whether one caused the other.

ACTION: We removed that sentence from the abstract (see point above for reviewer #1).

The statement in the discussion that "we identified a reduced repertoire of extracellular signalling ligands, which could explain the limited elaboration of its segmental body" has no basis. This is a classic case of the correlation versus causation problem. Maybe the body became less elaborate for some other reason, and then the animal did away with ligands because there was less of a need for cell-cell communication.

ACTION: We removed the phrase "which could explain the limited elaboration of its segmental body" from the text.

Then the authors say, "it is tempting to speculate that the simplification or dismantlement of distal cis-regulation observed in Dimorphilus might be one of the factors underpinning morphological miniaturisation". It is not at all obvious that gene loss or altered gene regulation should cause miniaturization. I urge the authors to resist this type of temptation. **ACTION:** We have removed this sentence and changed the end of this paragraph accordingly (lines 304-307).

This is a strong paper, and I think the authors should let the neat results shine on their own, without forcing conclusions or inferences that simply are not supported at this time.

Minor: in line 270, "motives" should be "motifs"

ACTION: Changed as suggested.

Reviewer #3 (Remarks to the Author):

Summary

--

I am generally happy that the authors have addressed my major concerns. In particular they have removed the section on the kynurenine pathway, which I think is the right choice. Equally they have moderated and made clearer the language regarding the link between molecular mechanisms and genome / body size. The ms as a whole contains much of interest and does not need less well supported stories.

ACTION: We thank the reviewer for the positive appraisal of the revised version of the manuscript.

Minor points

--

L.43 "we decode the 73.8 Mb long, compact genome" better rewritten as "we decode the compact 73.8 Mb genome ..." to avoid jarring juxtaposition of 'long' and 'compact'.

L.71 "nor the insect with arguably smallest body size has a compact genome" change to "[...], neither does the insect with arguably the smallest body size have a compact genome".

L.112 change to "a compact 92.47 Mb genome".

L.114 suggest changing to "our data suggest genome compaction and morphological miniaturisation both occurred in the lineage leading to [...]" to avoid ambiguity in interpretation of 'common' and ambiguity from singular use of 'event'.

L.149 'comparable' would be better than 'commensurable' unless the authors have intentionally chosen this.

L.172 delete 'Besides,'

L.270 change 'motives mostly close' to 'motifs, for the most part close [...]'

ACTION: We included all text changes as suggested.

Final Decision Letter:

15th September 2020

Dear Andi,

We are pleased to inform you that your Article entitled "Conservative route to extreme genome compaction in a miniature annelid", has now been accepted for publication in Nature Ecology & Evolution.

Before your manuscript is typeset, we will edit the text to ensure it is intelligible to our wide readership and conforms to house style. We look particularly carefully at the titles of all papers to ensure that they are relatively brief and understandable.

The subeditor may send you the edited text for your approval. Once your manuscript is typeset you will receive a link to your electronic proof via email within 20 working days, with a request to make any corrections within 48 hours. If you have queries at any point during the production process then please contact the production team at rjsproduction@springernature.com. Once your paper has been scheduled for online publication, the Nature press office will be in touch to confirm the details.

Acceptance of your manuscript is conditional on all authors' agreement with our publication policies

(see www.nature.com/authors/policies/index.html). In particular your manuscript must not be published elsewhere and there must be no announcement of the work to any media outlet until the publication date (the day on which it is uploaded onto our web site).

The Author's Accepted Manuscript (the accepted version of the manuscript as submitted by the author) may only be posted 6 months after the paper is published, consistent with our self-archiving embargo. Please note that the Author’s Accepted Manuscript may not be released under a Creative Commons license. For Nature Research Terms of Reuse of archived manuscripts please see: http://www.nature.com/authors/policies/license.html#terms
If you have posted a preprint on any preprint server, please ensure that the preprint details are updated with a publication reference, including the DOI and a URL to the published version of the article on the journal website.

An online order form for reprints of your paper is available at https://www.nature.com/reprints/author-reprints.html. All co-authors, authors' institutions and authors' funding agencies can order reprints using the form appropriate to their geographical region.

We welcome the submission of potential cover material (including a short caption of around 40 words) related to your manuscript; suggestions should be sent to Nature Ecology & Evolution as electronic files (the image should be 300 dpi at 210 x 297 mm in either TIFF or JPEG format). Please note that such pictures should be selected more for their aesthetic appeal than for their scientific content, and that colour images work better than black and white or grayscale images. Please do not try to design a cover with the Nature Ecology & Evolution logo etc., and please do not submit composites of images related to your work. I am sure you will understand that we cannot make any promise as to whether any of your suggestions might be selected for the cover of the journal.

You can now use a single sign-on for all your accounts, view the status of all your manuscript submissions and reviews, access usage statistics for your published articles and download a record of your refereeing activity for the Nature journals.

To assist our authors in disseminating their research to the broader community, our SharedIt initiative provides you with a unique shareable link that will allow anyone (with or without a subscription) to read the published article. Recipients of the link with a subscription will also be able to download and print the PDF.

You can generate the link yourself when you receive your article DOI by entering it here: <a href="http://authors.springernature.com/share">http://authors.springernature.com/share<a>.

[REDACTED]

P.S. Click on the following link if you would like to recommend Nature Ecology & Evolution to your librarian http://www.nature.com/subscriptions/recommend.html#forms

** Visit the Springer Nature Editorial and Publishing website at www.springernature.com/editorial-and-publishing-jobs for more information about our career opportunities. If you have any questions please click here.**
